# Design, Synthesis and Hepatoprotective Activity of Analogs of the Natural Product Goodyeroside A

**DOI:** 10.3390/molecules18021933

**Published:** 2013-02-01

**Authors:** Feng Zhang, Bei Han, Peng Li, Ziyun Lin, Dali Yin, Yan Li, Jialiang Zhong, Haihong Huang

**Affiliations:** 1 State Key Laboratory of Bioactive Substance and Function of Natural Medicines & Beijing Key Laboratory of Active Substances Discovery and Druggability Evaluation, Institute of Materia Medica, Peking Union Medical College and Chinese Academy of Medical Sciences, Beijing 100050, China; E-Mails: zfbj89@gmail.com (F.Z.); terryjordan@163.com (B.H.); qwerlik@imm.ac.cn (P.L.); linziyun@imm.ac.cn (Z.L.); yindali@imm.ac.cn (D.Y.); yanli@imm.ac.cn (Y.L); 2 Shanghai Institute of Pharmaceutical Industry, Shanghai 200040, China; E-Mail: jialiang.zhong@yahoo.com.cn

**Keywords:** goodyeroside A, structure-activity relationships, stereoselective, single crystal X-ray

## Abstract

Goodyeroside A, a natural product isolated from the *Goodyera* species, possesses significant hepatoprotective activity and has a novel skeleton not previously observed in other synthetic drugs used for the treatment of hepatitis. Herein, we report a highly stereoselective synthesis of goodyeroside A and related analogs with varying substituents at the α position of the carbonyl group to explore the structure-activity relationships of goodyeroside A. The absolute configuration of analog **5d** was confirmed by single crystal X-ray analysis. The results from *in vitro* and *in vivo* studies indicate that **5a**, the fully acetylated compound of goodyeroside A, is worthy of further investigation as a lead to identify novel hepatoprotective agents.

## 1. Introduction

**Figure 1:**
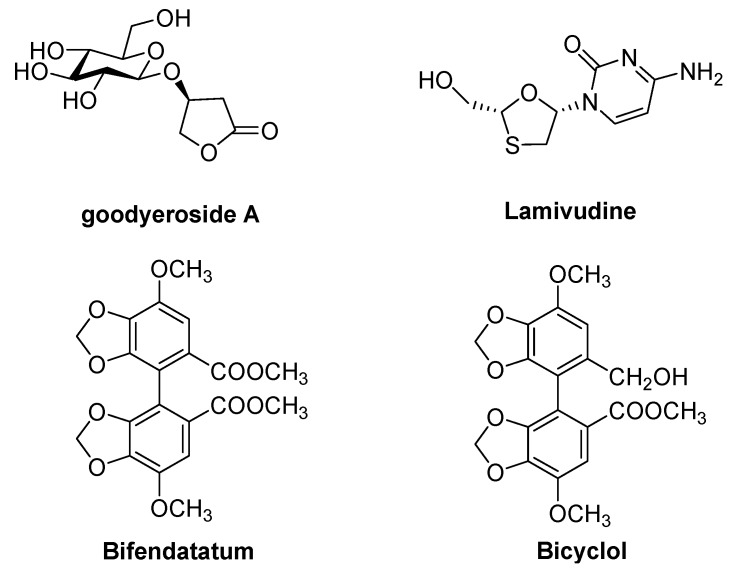
Goodyeroside A and some known drugs for hepatitis treatment.

The *Goodyera* species are orchidaceous perennial herbs grown natively in Japan and Southeast Asia. Since ancient times, the whole plant has been used as a folk medicine for the treatment of fever, pain, snake-bites and lung disease [1,2]. In 1999 [3], Gao and coworkers reported the isolation of a simple aliphatic glucoside, which was characterized as (3*S*)-3-(β-D-glucopyranosyloxy)butanolide (goodyeroside A), as a major active compound from the sprouts of *Crocus sativus* and later from the plants of three *Goodyera* species [4]. Preliminary biological tests showed that goodyeroside A had a significant hepatoprotective effect on primary cultured rat hepatocytes injured by CCl_4_ [4]. In comparison to other synthetic drugs used for hepatitis treatment such as bifendatatum [5], bicyclol [6,7] and lamivudine [8], goodyeroside A has a unique glycoside skeleton (Figure 1). This unique and rather simple structure evoked our interest in to modifying the structure in order to find new hepatoprotective agents.

Recently, the total synthesis of goodyeroside A was reported by Zhang and coworkers [9]. The development of this synthetic pathway provides opportunities to generate goodyeroside A analogs to explore their structure-activity relationships as hepatoprotective agents. This paper presents the details of initial structural modification of goodyeroside A and determination of the absolute configuration of analogs.

There are several positions in the structure of goodyeroside A that can be modified. We focused on introducing different substituents at the α position of the carbonyl in the initial stage of the synthesis. By stereoselectively introducing a series of substituents at the α position of the carbonyl, the conformation of the molecule can be altered, potentially influencing its hepatoprotective activity. In order to increase the compound’s lipophilicity, which may be beneficial in improving natural product pharmacokinetic properties, the fully acetylated products of goodyeroside A and its analogs were also synthesized.

## 2. Results and Discussion

### 2.1. The Synthesis of Goodyeroside A and Its Analogs

**Scheme 1:**
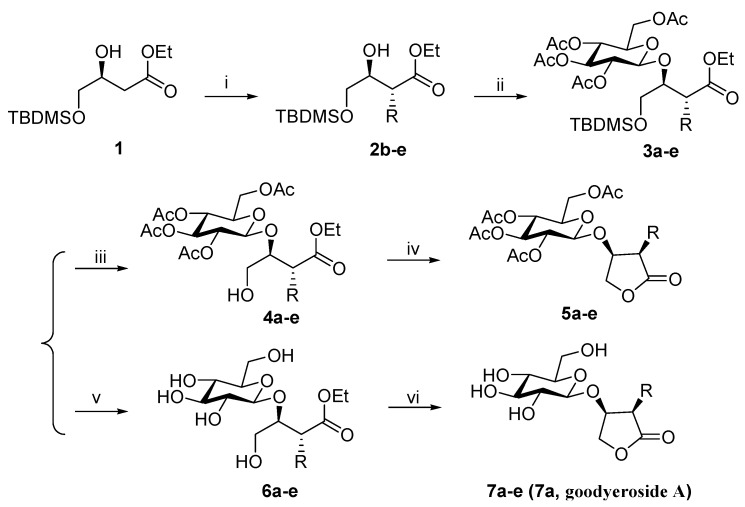
The synthetic route for goodyeroside A and its analogs.

The synthetic route for goodyeroside A and its derivatives is shown in Scheme 1. The chiral compound ethyl (*S*)-4-(*tert*-butyldimethylsilyloxy)-3-hydroxybutanoate (**1**) is the key synthon in the methodology described here and was prepared from (*S*)-malic acid as described in the literature [9]. The introduction of an alkyl group at the α position of carbonyl in **1** was improved by reinvestigation of the reported method [10]. Thus, the β-hydroxy ester was treated with lithium hexamethyldisilazide (LiHMDS, 2.0 equiv.) in the presence of 1,3-dimethyl-2-imidazolidinone (DMI, 2.5 equiv.) in THF at −45 °C, and the alkylating agent was then reacted with the dianion generated at the same temperature to produce the alkylated products **2**. Under these reaction conditions, α-alkylation proceeded in a highly stereoselective manner to give a single diastereoisomer and no stereoisomer was detected.

Construction of the β-glucoside was accomplished using the imidate method [11]. The use of 2,3,4,6-tetra-*O*-acetyl-α-D-glucopyranosyl trichloroacetimidate [12] as glycosyl donor led to the corresponding β-glucoside **3** in moderate yields. The newly formed glycosidic bonds were proved to be exclusively 1,2-*trans* on the basis of the ^1^H-NMR spectra. Removal of the *t*-butyldimethylsilyl (TBDMS) protecting group under acidic conditions such as *p*-toluenesulfonic acid (PTSA) [13] or 10-camphorsulfonic acid (CSA), generated desired compounds **4** and the lactonized products **5**. Even under neutral conditions such as the use of ceric ammonium nitrate (CAN) [14], a similar mixture of products **4** and **5** were produced. Only with tetrabutylammonium fluoride (TBAF) [15], were compounds **4** formed cleanly. Treatment of alcohols **4** with PTSA [16] (1.0 equiv.) produced the optically pure cyclization products (2*R*,3*S*)-**5** and with no detection of epimeric lactones. The absolute configuration of lactone **5d** was determined by X-ray crystallographic analysis.

Initially, we attempted to synthesize compounds **6** from **4** under NaOEt/EtOH conditions, but the reactions failed to give the desired products. We then inverted the sequence of the silyl and acetyl deprotections, treated compounds **3** with NaOEt/EtOH to remove the four acetyls, and then treated with TBAF to achieve desilylation, resulting in pure products **6** from **3** in good yields. The subsequent cyclization of **6** to obtain **7** was accomplished by PTSA treatment. The spectral data of the acetylated derivative obtained from **7d** under Ac_2_O/pyridine conditions was identical with that of the corresponding acetylated compound **5d**, which showed that the absolute configuration of deacetylated lactone **7** was consistent with that of acetylated lactone **5**.

### 2.2. Absolute Configuration of Compound ***5d*** by X-ray Analysis

**Figure 2:**
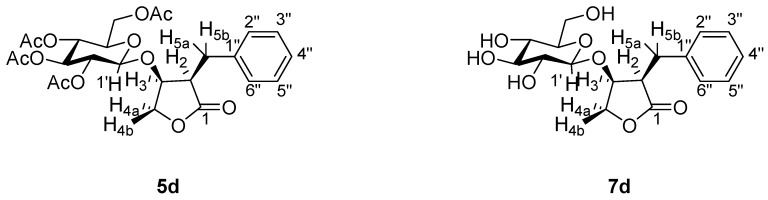
Structures of compounds **5d** and **7d**.

All target compounds were characterized by ^1^H-NMR, ^13^C-NMR and HRMS spectra. A one-dimensional NOE study performed on compound **5d** and **7d** showed that the configuration of the newly generated chiral center C-2 is the *R*-configuration, deduced from the observation of a positive NOE at H-3 and H-4a upon irradiation H-2, a positive NOE at H-2, H-4a and H-4b upon irradiation H-3, and that the extent of positive NOE at H-4a is higher than that of H-4b (Figure 2).

In order to confirm the exact structure of compounds **5**, X-ray crystallographic analysis of the corresponding (2*R*,3*S*)-2-benzyl-3-(2,3,4,6-tetra-*O*-acetyl-β-D-glucopyranosyloxy)butanolide (**5d**) was investigated. After various efforts, single crystals of **5d** suitable for X-ray analysis were obtained by slow evaporation of a petroleum ether/EtOAc solution over a period of two weeks. X-ray data were collected on a Bruker SMART APEX II CCD diffractometer with an Oxford Cryostream low-temperature device using graphite monochromatic Cu Kα (λ = 1.54178 Ǻ) radiation, operating at 153(2)K, over the *θ* range 4.42°–66.00°. The compound **5d** crystallized in orthorhombic, space group P2_1_2_1_2_1_. Crystal data of **5d**: C_25_H_30_O_12_, molecular weight 522.49, D_c_ = 1.282g/cm^3^, a = 1.06142(2) nm, b = 1.10984(2) nm, c = 2.29714(3) nm, v = 2.70605(8) nm^3^, z = 4, μ = 0.874, F(000) = 1104. The absolute configuration was confirmed by the Flack parameter 0.05 (17) and the Hooft parameter 0.04 (6). Full crystallographic data could be obtained from *Acta Crystallographica Section E* [17].

**Figure 3:**
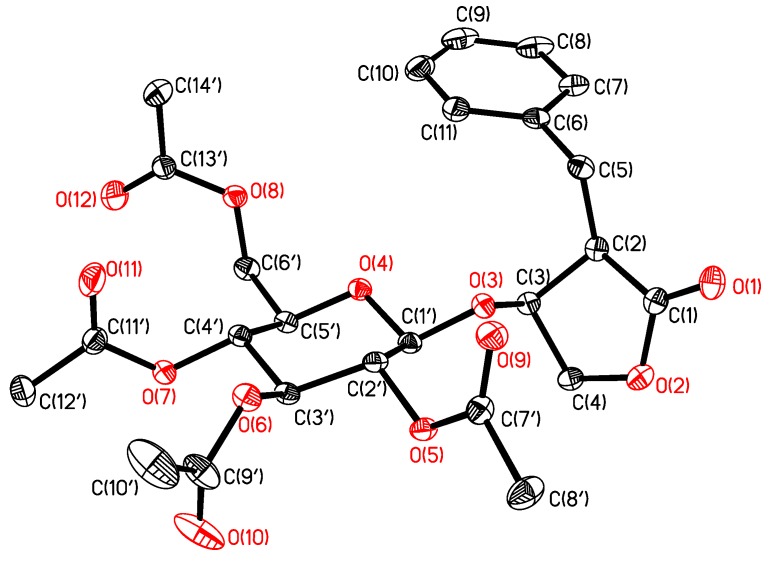
Perspective view of the crystal structure of compound **5d**.

The result of the X-ray analysis showed that the bond lengths and angles were in agreement with literature values [18]. The benzene ring (C6-C11) (ring A) was essentially planar, the five-membered ring (C1-C4/O2) (ring B) had an envelope conformation, and the six-membered ring (C1’-C5’/O4) (ring C) was in its chair conformation. The dihedral angels between the various rings were as follows: A/B 112.9(1)°; B/C 110.1(1)°; A/C 13.1(1)°. From the projection of molecular structure, the absolute configuration was confirmed to be the 2*R*,3*S*-configurations (Figure 3).

### 2.3. Biological Results and Discussion

**Figure 4:**
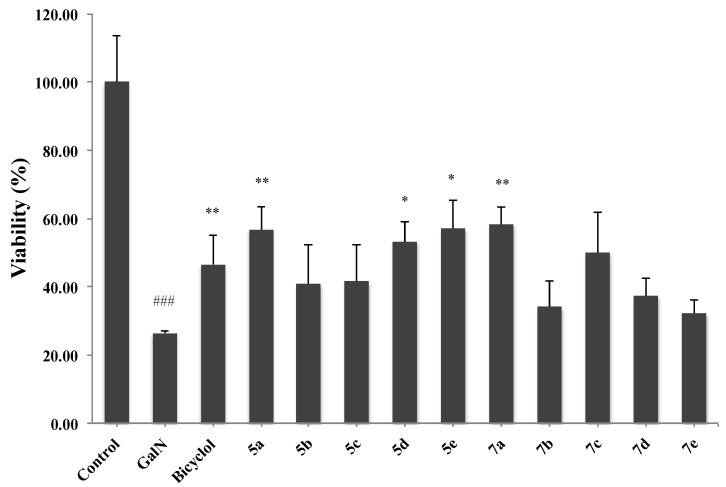
Hepatoprotective activity of goodyeroside A and its analogs on GalN-induced rat hepatocyte injury.

To assess the biological activity of the newly synthesized goodyeroside A analogs as hepatoprotective agents, a rat hepatic oval cell injury model induced by D-galactosamine (GalN) was adopted as the induction of liver injuries by GalN is a suitable experimental model of human liver failure [19]. The reduction in the intracellular level of uridylate is responsible for GalN-induced apoptosis and necrosis in primary cultured rat hepatocytes [20,21]. Goodyeroside A and its nine analogs were evaluated for their ability to inhibit GalN-induced hepatocyte injury, using the clinical hepatoprotective drug Bicyclol as a positive control. Results are shown in Figure 4. At a concentration of 10^−4^ M, all the analogs exhibited hepatoprotective activity to certain extents. The fully acetylated products **5** showed relatively higher activity compared with compounds **7** although the difference was not significant. Among the fully acetylated products, analogs **5d**,**e** with aromatic substituents at the α-carbonyl position showed higher activity than analogs **5b**,**c** with small aliphatic substituents at the α-carbonyl. Goodyeroside A (**7a**) and its fully acetylated product **5a** showed strong hepatoprotective activity but did not have a significant difference in comparison to bicyclol.

ConA-induced acute liver injury models were used in the present study to investigate the *in vivo* activity of goodyeroside A (**7a**) and its fully acetylated product **5a**. As shown in Table 1, compound **5a** exhibited significant hepatoprotective effects as indicated by decreasing ALT and AST levels, while the natural product goodyeroside A (**7a**) offered no protection. These results further confirmed our hypothesis that the full-acetylation may improve pharmacokinetic properties by increasing the lipophilicity of the compound.

## 3. Experimental

### 3.1. General

Melting points were determined on a Yanaco micrometer and are uncorrected. NMR spectra were taken on a Mercury-300, Mercury-400, INOVA-500 or INOVA-600 spectrometers. HRMS spectra were recorded on an Agilent LC-MSD TOF instrument. The optical rotation was obtained on a PerkinElmer 341 LC Polarimeter. Flash chromatography was accomplished by using silica gel H and column chromatography was run on silica gel (200–300 mesh). EtOH was dried by distillation from Mg. THF was distilled from Na/benzophenone under a N_2_ atmosphere. CH_2_Cl_2_ was dried by distillation from P_2_O_5_.

### 3.2. General Method for Preparation of Compounds ***2***

*n*-Butyllithium (2.5 M, 2.2 equiv.) was added dropwise to a solution of HMDS (2.4 equiv.) in THF at −10 °C under nitrogen. Then the solution was stirred at rt for 30 min and then cooled to −45 °C. A solution of the ethyl (*S*)-4-(*tert*-butyldimethylsilyloxy)-3-hydroxybutanoate (**1**, 1.0 equiv.) in THF was added and the residual ester was washed with further THF to the above mixture. After stirring for 30 min at the same temperature, alkylating agent (1.2 equiv.) and 1,3-dimethyl-2-imidazolidinone (DMI, 2.5 equiv.) in THF were added dropwise. After stirring for 30 min at the same temperature, saturated aqueous ammonium chloride was added to the reaction mixture and the mixture was extracted with ethyl acetate. The organic layer was washed with water and saturated brine, dried over magnesium sulfate, and concentrated *in vacuo*. The residue was purified by silica gel chromatography to give the products **2**.

*Ethyl (2R,3S)-4-(tert-butyldimethylsilyloxy)-3-hydroxy-2-methylbutanoate* (**2b**) [22,23]. Yellow oil, yield 59%. [α${]}_{D}^{20}$ −12.49 (c 1.14, CHCl_3_). ^1^H-NMR (300 MHz, CDCl_3_): δ = 0.10 (s, 6 H, Si(CH_3_)_2_), 0.94 (s, 9 H, SiC(CH_3_)_3_), 1.19 (d, *J* = 6.9 Hz, 3 H, CHCH_3_), 1.28 (t, *J* = 7.2 Hz, 3 H, CH_2_CH_3_), 2.60~2.73 (m, 1 H, H-2), 3.60~3.68 (m, 2 H, H-4), 3.71~3.75 (m, 1 H, H-3), 4.18 (q, *J* = 7.2 Hz, 2 H, CH_2_CH_3_). HRMS (ESI-TOF^+^): *m/z* [M+H]^+^ calcd for C_13_H_29_O_4_Si: 277.1835; found: 277.1807; [M+Na]^+^ calcd for C_13_H_28_O_4_SiNa: 299.1655; found: 299.1634.

*Ethyl (2R,3S)-2-allyl-4-(tert-butyldimethylsilyloxy)-3-hydroxybutanoate* (**2c**). Pale yellow oil, yield 59%. [α${]}_{D}^{20}$ −10.28 (c 1.19, CHCl_3_). ^1^H-NMR (300 MHz, CDCl_3_): δ = 0.06 (s, 6 H, Si(CH_3_)_2_), 0.89 (s, 9 H, SiC(CH_3_)_3_), 1.26 (t, *J* = 7.2 Hz, 3 H, CH_2_CH_3_), 2.31~2.48 (m, 2 H, CH_2_CH=CH_2_), 2.63~2.70 (m, 1 H, H-2), 3.60~3.68 (m, 2 H, H-4), 3.74~3.80 (m, 1 H, H-3), 4.08~4.20 (m, 2 H, CH_2_CH_3_), 5.07 (dd, *J* = 10.5, 20.4 Hz, 2 H, CH_2_CH=CH_2_), 5.68~5.82 (m, 1 H, CH_2_CH=CH_2_). HRMS (ESI-TOF^+^): *m/z* [M+H]^+^ calcd for C_15_H_31_O_4_Si: 303.1992; found: 303.1963.

*Ethyl (2R,3S)-2-benzyl-4-(tert-butyldimethylsilyloxy)-3-hydroxybutanoate* (**2d**). Pale yellow oil, yield 54%. [α${]}_{D}^{20}$ +17.43 (c 1.00, CHCl_3_). ^1^H-NMR (300 MHz, CDCl_3_): δ = 0.04 (s, 6 H, Si(CH_3_)_2_), 0.89 (s, 9 H, SiC(CH_3_)_3_), 1.10 (t, *J* = 7.2 Hz, 3 H, CH_2_CH_3_), 2.85~3.00 (m, 2 H, CH_2_Ph), 3.09 (br s, 1 H, H-2), 3.65 (d, *J* = 5.1 Hz, 2 H, H-4), 3.75 (br s, 1 H, H-3), 3.99~4.10 (m, 2 H, CH_2_CH_3_), 7.18~7.30 (m, 5 H, Ar-H). HRMS (ESI-TOF^+^): *m/z* [M+Na]^+^ calcd for C_19_H_32_O_4_SiNa: 375.1968; found: 375.2007.

*Ethyl (2R,3S)-4-(tert-butyldimethylsilyloxy)-3-hydroxy-2-(4-methoxybenzyl)butanoate* (**2e**). Pale yellow oil, yield 62%. [α${]}_{D}^{20}$ +16.91 (c 1.45, CHCl_3_). ^1^H-NMR (300 MHz, CDCl_3_): δ = 0.05 (s, 6 H, Si(CH_3_)_2_), 0.89 (s, 9 H, SiC(CH_3_)_3_), 1.12 (t, *J* = 7.2 Hz, 3H, CH_2_CH_3_), 2.79~2.94 (m, 3 H, H-2, CH_2_Ph), 3.64 (d, *J* = 4.8 Hz, 2 H, H-4), 3.71~3.74 (m, 1 H, H-3), 3.78 (s, 3 H, OCH_3_), 4.01~4.10 (m, 2 H, CH_2_CH_3_), 6.81 (d, *J* = 6.6 Hz, 2 H, Ar-H), 7.11 (d, *J* = 6.6 Hz, 2 H, Ar-H). HRMS (ESI-TOF^+^): *m/z* [M+H]^+^ calcd for C_20_H_35_O_5_Si: 383.2254; found: 383.2288.

### 3.3. General Method for Preparation of Compounds ***3***

A suspension of **2** (1.0 equiv), 2,3,4,6-tetra-*O*-acetyl-α-D-glucopyranosyl trichloroacetimidate (1.5 equiv.), and activated 4 Å powdered molecular sieves in freshly distilled anhydrous CH_2_Cl_2_ was vigorously stirred for 1 h at rt under nitrogen. To this suspension was added TMSOTf (0.2 equiv.) at −20 °C. Then the mixture was allowed to warm to rt and stirred at rt for 30 min, the reaction was quenched with the addition of Et_3_N. The mixture was filtered and concentrated *in vacuo*. The residue was purified by column chromatography to give the products **3**.

*Ethyl (S)-4-(tert-Butyldimethylsilyloxy)-3-(2,3,4,6-tetra-O-acetyl-β-molecules-18-01933D-glucopyranosyloxy)butanoate* (**3a**). Pale yellow syrup, yield 60%. [α${]}_{D}^{20}$ −10.1 (c 1.06, CHCl_3_). ^1^H-NMR (300 MHz, CDCl_3_): δ = 0.04 (s, 6 H, Si(CH_3_)_2_), 0.89 (s, 9 H, SiC(CH_3_)_3_), 1.25 (t, *J* = 7.2 Hz, 3 H, CH_2_CH_3_), 1.99~2.08 (4s, 12 H, 4 × CH_3_CO), 2.59 (d, *J* = 6.3 Hz, 2 H, H-2), 3.56 (dd, *J* = 5.4, 10.5 Hz, 1 H, H-4a), 3.62~3.67 (m, 2 H, H-4b, H-5’), 4.07 (dd, *J* = 2.7, 12.3 Hz, 1 H, H-6’a), 4.12 (q, *J* = 7.2 Hz, 2 H, CH_2_CH_3_), 4.10~4.18 (m, 1 H, H-3), 4.27 (dd, *J* = 4.5, 12.3 Hz, 1 H, H-6’b), 4.75 (d, *J* = 8.1 Hz, 1 H, H-1’), 4.94 (dd, *J* = 8.1, 9.3 Hz, 1 H, H-2’), 5.07 (t, *J* = 9.6 Hz, 1 H, H-4’), 5.16 (t, *J* = 9.3 Hz, 1 H, H-3’). HRMS (ESI-TOF^+^): *m/z* [M+Na]^+^ calcd for C_26_H_44_O_13_SiNa: 615.2449; found: 615.2444.

*Ethyl (2R,3S)-4-(tert-butyldimethylsilyloxy)-2-methyl-3-(2,3,4,6-tetra-O-acetyl-β-molecules-18-01933D-glucopyranosyloxy)butanoate* (**3b**). Colorless syrup, yield 65%. [α${]}_{D}^{20}$ +13.59 (c 1.03, CHCl_3_). ^1^H-NMR (300 MHz, CDCl_3_): δ = 0.05 (s, 6 H, Si(CH_3_)_2_), 0.89 (s, 9 H, SiC(CH_3_)_3_), 1.12 (d, *J* = 6.9 Hz, 3 H, CHCH_3_), 1.25 (t, *J* = 7.5 Hz, 3 H, CH_2_CH_3_), 1.99~2.07 (4s, 12 H, 4 × CH_3_CO), 2.78~2.82 (m, 1 H, H-2), 3.62~3.72 (m, 3 H, H-4a, H-4b, H-5’), 4.01~4.15 (m, 4 H, H-3, H-6’a, CH_2_CH_3_), 4.18 (dd, *J* = 5.1, 12.3 Hz, 1 H, H-6’b), 4.80 (d, *J* = 7.8 Hz, 1 H, H-1’), 4.95 (dd, *J* = 7.8, 9.0 Hz, 1 H, H-2’), 5.05 (dd, *J* = 9.0, 9.6 Hz, 1 H, H-4’), 5.16 (dd, *J* = 9.0, 9.6 Hz, 1 H, H-3’). HRMS (ESI-TOF^+^): *m/z* [M+Na]^+^ calcd for C_27_H_46_O_13_SiNa: 629.2605; found: 629.2576.

*Ethyl (2R,3S)-2-allyl-4-(tert-butyldimethylsilyloxy)-3-(2,3,4,6-tetra-O-acetyl-β-molecules-18-01933D-glucopyranosyloxy)butanoate* (**3c**). Pale yellow syrup, yield 68%. [α${]}_{D}^{20}$ −11.55 (c 1.17, CHCl_3_). ^1^H-NMR (300 MHz, CDCl_3_): δ = 0.06 (s, 6 H, Si(CH_3_)_2_), 0.91 (s, 9 H, SiC(CH_3_)_3_), 1.24 (t, *J* = 7.2 Hz, 3 H, CH_2_CH_3_), 2.00~2.08 (4s, 12 H, 4 × CH_3_CO), 2.23~2.46 (m, 2 H, CH_2_CH=CH_2_), 2.70~2.76 (m, 1 H, H-2), 3.62~3.66 (m, 1 H, H-5’), 3.68 (dd, *J* = 5.7, 10.5 Hz, 1 H, H-4a), 3.75 (dd, *J* = 4.2, 10.5 Hz, 1 H, H-4b), 3.96~4.01 (m, 1 H, H-3), 4.04~4.17 (m, 3 H, CH_2_CH_3_, H-6’a), 4.25 (dd, *J* = 4.8, 12.3 Hz, 1 H, H-6’b), 4.80 (d, *J* = 8.1 Hz, 1 H, H-1’), 4.95 (dd, *J* = 8.1, 9.6 Hz, 1 H, H-2’), 5.02~5.09 (m, 2 H, CH_2_CH=CH_2_), 5.06 (dd, *J* = 9.3, 9.6 Hz, 1 H, H-4’), 5.16 (dd, *J* = 9.3, 9.6 Hz, 1 H, H-3’), 5.67~5.81 (m, 1 H, CH_2_CH=CH_2_). HRMS (ESI-TOF^+^): *m/z* [M+Na]^+^ calcd for C_29_H_48_O_13_SiNa: 655.2762; found: 655.2755.

*Ethyl (2R,3S)-2-molecules-18-01933benzyl-4-(tert-butyldimethylsilyloxy)-3-(2,3,4,6-tetra-O-acetyl-β-molecules-18-01933D-glucopyranosyloxy)butanoate* (**3d**). Pale yellow syrup, yield 76%. [α${]}_{D}^{20}$ −11.9 (c 1.03, CHCl_3_). ^1^H-NMR (300 MHz, CDCl_3_): δ = 0.04 (s, 6 H, Si(CH_3_)_2_), 0.88 (s, 9 H, SiC(CH_3_)_3_), 1.14 (t, *J* = 7.2 Hz, 3 H, CH_2_CH_3_), 2.00~2.08 (4s, 12 H, 4 × CH_3_CO), 2.93 (m, 2 H, H-2, CH_2_Ph), 3.00 (dd, *J* = 4.2, 7.5 Hz, 1 H, CH_2_Ph), 3.64~3.68 (m, 1 H, H-5’), 3.72 (d, *J* = 11.1 Hz, 1 H, H-4a), 3.77 (dd, *J* = 4.5, 11.1 Hz, 1 H, H-4b), 3.96~4.08 (m, 3 H, H-3, CH_2_CH_3_), 4.13 (dd, *J* = 2.1, 12.3 Hz, 1 H, H-6’a), 4.28 (dd, *J* = 4.5, 12.3 Hz, 1 H, H-6’b), 4.82 (d, *J* = 7.8 Hz, 1 H, H-1’), 5.00 (dd, *J* = 7.8, 9.0 Hz, 1 H, H-2’), 5.10 (dd, *J* = 9.3 Hz, 1 H, H-4’), 5.18 (dd, *J* = 9.0, 9.3 Hz, 1 H, H-3’), 7.14~7.24 (m, 5 H, Ar-H). HRMS (ESI-TOF^+^): *m/z* [M+Na]^+^ calcd for C_33_H_50_O_13_SiNa: 705.2918; found: 705.2938.

*Ethyl (2R,3S)-4-(tert-butyldimethylsilyloxy)-2-(4-methoxybenzyl)-3-(2,3,4,6-tetra-O-acetyl-β-molecules-18-01933D-gluco-pyranosyloxy)butanoate* (**3e**). Pale yellow syrup, yield 67%. [α${]}_{D}^{20}$ −10.70 (c 1.39, CHCl_3_). ^1^H-NMR (300 MHz, CDCl_3_): δ = 0.04 (s, 6 H, Si(CH_3_)_2_), 0.89 (s, 9 H, SiC(CH_3_)_3_), 1.16 (t, *J* = 7.2 Hz, 3 H, CH_2_CH_3_), 2.00~2.06 (4s, 12 H, 4 × CH_3_CO), 2.85~2.88 (m, 2 H, H-2, CH_2_Ph), 2.95 (dd, *J* = 3.9, 7.2 Hz, 1 H, CH_2_Ph), 3.64~3.68 (m, 1 H, H-5’), 3.77 (s, 3 H, OCH_3_), 3.79~3.80 (m, 2 H, H-4a, H-4b), 3.95~4.00 (m, 1 H, H-3), 4.04 (q, *J* = 7.2 Hz, 2 H, CH_2_CH_3_), 4.09~4.16 (m, 1 H, H-6’a), 4.28 (dd, *J* = 4.5, 12.0 Hz, 1 H, H-6’b), 4.83 (d, *J* = 7.8 Hz, 1 H, H-1’), 5.00 (dd, *J* = 7.8, 8.7 Hz, 1 H, H-2’), 5.10 (t, *J* = 9.3 Hz, 1 H, H-4’), 5.18 (dd, *J* = 9.0, 9.3 Hz, 1 H, H-3’), 6.78 (d, *J* = 8.4 Hz, 2 H, Ar-H), 7.11 (d, *J* = 8.4 Hz, 2 H, Ar-H). HRMS (ESI-TOF^+^): *m/z* [M+Na]^+^ calcd for C_34_H_52_O_14_SiNa: 735.3024; found: 735.3067.

### 3.4. General Method for Preparation of Compounds ***4***

To a solution of **3** (1.0 equiv) in anhydrous THF was added TBAF (1.0 M in THF, 0.5 equiv). The mixture was stirred at rt until TLC showed no starting material. Then the mixture was concentrated *in vacuo*. The residue was purified by column chromatography to give the products **4**.

*Ethyl (S)-4-molecules-18-01933hydroxy-3-(2,3,4,6-tetra-O-acetyl-β-molecules-18-01933D-glucopyranosyloxy)butanoate* (**4a**). Yellow syrup, yield 60%. [α${]}_{D}^{20}$ −10.87 (c 1.01, CHCl_3_). ^1^H-NMR (300 MHz, CDCl_3_): δ = 1.27 (t, *J* = 7.2 Hz, 3 H, CH_2_CH_3_), 2.01~2.09 (4s, 12 H, 4 × CH_3_CO), 2.54 (dd, *J* = 6.6, 16.5 Hz, 1 H, H-2a), 2.74 (dd, *J* = 3.0, 16.5 Hz, 1 H, H-2b), 3.58 (dd, *J* = 6.6, 12.0 Hz, 1 H, H-4a), 3.66 (dd, *J* = 3.0, 12.0 Hz, 1 H, H-4b), 3.68~3.72 (m, 1 H, H-5’), 4.06~4.22 (m, 4 H, H-6’a, CH_2_CH_3,_ H-3), 4.26 (dd, *J* = 4.5, 12.3 Hz, 1 H, H-6’b), 4.74 (d, *J* = 8.1 Hz, 1 H, H-1’), 4.97 (dd, *J* = 8.1, 9.3 Hz, 1 H, H-2’), 5.07 (dd, *J* = 9.6, 9.9 Hz, 1 H, H-4’), 5.21 (dd, *J* = 9.3, 9.6 Hz, 1 H, H-3’). HRMS (ESI-TOF^+^): *m/z* [M+Na]^+^ calcd for C_20_H_30_O_13_Na: 501.1584; found: 501.1599.

*Ethyl (2R,3S)-4-hydroxy-2-methyl-3-(2,3,4,6-tetra-O-acetyl-β-molecules-18-01933D-glucopyranosyloxy)butanoate* (**4b**). Yellow syrup, yield 50%. [α${]}_{D}^{20}$ −14.39 (c 3.21, CHCl_3_). ^1^H-NMR (300 MHz, CDCl_3_): δ = 1.14 (d, *J* = 9.0 Hz, 3 H, CHCH_3_), 1.26 (t, *J* = 7.2 Hz, 3 H, CH_2_CH_3_), 2.00~2.09 (4s, 12 H, 4 × CH_3_CO), 2.89 (m, 1 H, H-2), 3.67~3.80 (m, 3 H, H-4a, H-4b, H-5’), 4.01~4.41 (m, 4 H, H-3, H-6’a, CH_2_CH_3_), 4.63 (dd, *J* = 8.4, 16.8 Hz, 1 H, H-6’b), 4.74 (d, *J* = 8.4 Hz, 1 H, H-1’), 5.02 (dd, *J* = 8.4, 9.0 Hz, 1 H, H-2’), 5.09 (dd, *J* = 9.9, 10.8 Hz, 1 H, H-4’), 5.17 (dd, *J* = 9.0, 9.9 Hz, 1 H, H-3’). HRMS (ESI-TOF^+^): *m/z* [2M+Na]^+^ calcd for C_42_H_64_O_26_Na: 1007.3584; found: 1007.3549.

*Ethyl (2R,3S)-2-allyl-4-hydroxy-3-(2,3,4,6-tetra-O-acetyl-β-molecules-18-01933D-glucopyranosyloxy)butanoate* (**4c**). Pale yellow syrup, yield 75%. [α${]}_{D}^{20}$ −20.45 (c 2.92, CHCl_3_). ^1^H-NMR (300 MHz, CDCl_3_): δ = 1.24 (t, *J* = 7.5 Hz, 3 H, CH_2_CH_3_), 1.98~2.06 (4s, 12 H, 4 × CH_3_CO), 2.33 (dd, *J* = 6.9, 7.2 Hz, 2 H, CH_2_CH=CH_2_), 2.82 (m, 1 H, H-2), 3.61~3.72 (m, 3 H, H-4a, H-4b, H-5’), 3.98 (q, *J* = 4.2, 5.7 Hz, 1 H, H-3), 4.08~4.20 (m, 3 H, CH_2_CH_3_, H-6’a), 4.21 (dd, *J* = 5.4, 12.3 Hz, 1 H, H-6’b), 4.69 (d, *J* = 7.8 Hz, 1 H, H-1’), 4.93~5.08 (m, 4 H, H-2’, H-4’, CH_2_CH=CH_2_), 5.18 (dd, *J* = 9.3, 9.6 Hz, 1 H, H-3’), 5.64~5.78 (m, 1 H, CH_2_CH=CH_2_). HRMS (ESI-TOF^+^): *m/z* [M+Na]^+^ calcd for C_23_H_34_O_13_Na: 541.1897; found: 541.1866.

*Ethyl (2R,3S)-2-benzyl-4-hydroxy-3-(2,3,4,6-tetra-O-acetyl-β-molecules-18-01933D-glucopyranosyloxy)butanoate* (**4d**). Colorless syrup, yield 74%. [α${]}_{D}^{20}$ −5.81 (c 1.15, CHCl_3_). ^1^H-NMR (300 MHz, CDCl_3_): δ = 1.11 (t, *J* = 7.2 Hz, 3 H, CH_2_CH_3_), 2.01~2.19 (4s, 12 H, 4 × CH_3_CO), 2.87 (dd, *J* = 10.5, 13.5 Hz, 1 H, CH_2_Ph), 2.96 (dd, *J* = 4.8, 13.5 Hz, 1 H, CH_2_Ph), 3.10~3.16 (m, 1 H, H-2), 3.73~3.77 (m, 3 H, H-4a, H-4b, H-5’), 3.96~4.06 (m, 3 H, H-3, CH_2_CH_3_), 4.13 (dd, *J* = 2.4, 12.3 Hz, 1 H, H-6’a), 4.26 (dd, *J* = 5.1, 12.3 Hz, 1 H, H-6’b), 4.75 (d, *J* = 7.8 Hz, 1 H, H-1’), 5.04 (dd, *J* = 7.8, 9.6 Hz, 1 H, H-2’), 5.10 (dd, *J* = 9.6, 9.9 Hz, 1 H, H-4’), 5.23 (dd, *J* = 9.3, 9.6 Hz, 1 H, H-3’), 7.17~7.24 (m, 5 H, Ar-H). HRMS (ESI-TOF^+^): *m/z* [M+Na]^+^ calcd for C_27_H_36_O_13_Na: 591.2054; found: 591.2021.

*Ethyl (2R,3S)-4-hydroxy-2-(4-methoxybenzyl)-3-(2,3,4,6-tetra-O-acetyl-βmolecules-18-01933-molecules-18-01933Dmolecules-18-01933-glucopyranosyloxy)butanoate* (**4e**). Colorless syrup, yield 67%. [α${]}_{D}^{20}$ −0.77 (c 1.29, CHCl_3_). ^1^H-NMR (300 MHz, CDCl_3_): δ = 1.12 (t, *J* = 7.2 Hz, 3 H, CH_2_CH_3_), 2.00~2.12 (4s, 12 H, 4 × CH_3_CO), 2.80 (dd, *J* = 10.2, 13.5 Hz, 1 H, CH_2_Ph), 2.89 (dd, *J* = 4.5, 13.5 Hz, 1 H, CH_2_Ph), 3.03~3.10 (m, 1 H, H-2), 3.72~3.73 (m, 3 H, H-4a, H-4b, H-5’), 3.76 (s, 3 H, OCH_3_), 3.99~4.06 (m, 3 H, CH_2_CH_3_, H-3), 4.13 (dd, *J* = 1.8, 12.3 Hz, 1 H, H-6’a), 4.25 (dd, *J* = 5.1, 12.3 Hz, 1 H, H-6’b), 4.74 (d, *J* = 7.8 Hz, 1 H, H-1’), 5.02 (dd, *J* = 7.8, 9.6 Hz, 1 H, H-2’), 5.09 (m, 1 H, H-4’), 5.21 (m, 1 H, H-3’), 6.77 (d, *J* = 8.7 Hz, 2 H, Ar-H), 7.09 (d, *J* = 8.7 Hz, 2 H, Ar-H). HRMS (ESI-TOF^+^): *m/z* [M+Na]^+^ calcd for C_28_H_38_O_14_Na: 621.2159; found: 621.2183.

### 3.5. General Method for Preparation of Compounds ***5***

To a solution of **4** (1.0 equiv.) in 1,4-dioxane was added PTSA·H_2_O (1.0 equiv.). The mixture was stirred at rt until TLC showed no starting material. Then the mixture was concentrated *in vacuo*. The residue was purified by column chromatography to give the products **5**.

*(3S)-3-(2,3,4,6-molecules-18-01933Tmolecules-18-01933etra-O-acetyl-βmolecules-18-01933-molecules-18-01933Dmolecules-18-01933-glucopyranosyloxy)butanolide* (**5a**). White solid, yield 58%. Mp 126.5–127.5 °C (lit. [24] 126–127.5 °C); [α${]}_{D}^{20}$ −43.7 (c 1.01, CHCl_3_) (lit. [24] [α${]}_{D}^{15}$ −47.4 (c 0.09, CHCl_3_)). ^1^H-NMR (300 MHz, CDCl_3_): δ = 2.01~2.10 (4s, 12 H, 4 × CH_3_CO), 2.74~2.75 (m, 2 H, H-2), 3.69~3.74 (m, 1 H, H-5’), 4.15 (dd, *J* = 2.1, 12.3 Hz, 1 H, H-6’a), 4.24 (dd, *J* = 2.1, 12.3 Hz, 1 H, H-6’b), 4.31 (dd, *J* = 2.1, 10.5 Hz, 1 H, H-4a), 4.39 (dd, *J* = 1.8, 10.5 Hz, 1 H, H-4b), 4.63 (m, 2 H, H-1’, H-3), 4.99 (dd, *J* = 7.8, 9.6 Hz, 1 H, H-2’), 5.08 (dd, *J* = 9.6, 9.9 Hz, 1 H, H-4’), 5.21 (dd, *J* = 9.3, 9.9 Hz, 1 H, H-3’). HRMS (ESI-TOF^+^): *m/z* [M+Na]^+^ calcd for C_18_H_24_O_12_Na: 455.1165; found: 455.1131.

*(2R,3S)-2-molecules-18-01933Methylmolecules-18-01933-3-(2,3,4,6-tetra-O-acetyl-βmolecules-18-01933-molecules-18-01933Dmolecules-18-01933-glucopyranosyloxy)butanolide* (**5b**). White solid, yield 61%. Mp 150–151 °C; [α${]}_{D}^{20}$ −69.45 (c 0.53, CHCl_3_). ^1^H-NMR (300 MHz, CDCl_3_): δ = 1.24 (d, *J* = 7.0 Hz, 3 H, CHCH_3_), 2.01~2.09 (m, 12 H, 4 × CH_3_CO), 2.63~2.73 (m, 1 H, H-2), 3.66~3.72 (m, 1 H, H-5’), 4.14~4.24 (m, 3 H, H-6’a, H-6’b, H-4a), 4.30 (d, *J* = 5.1 Hz, 1 H, H-4b), 4.57 (dd, *J* = 3.0, 5.1 Hz, 1 H, H-3), 4.63(d, *J* = 8.1 Hz, 1 H, H-1’), 4.99 (dd, *J* = 8.1, 9.3 Hz, 1 H, H-2’), 5.10 (dd, *J* = 9.3, 9.6 Hz, 1 H, H-4’), 5.21 (dd, *J* = 9.3, 9.3 Hz, 1 H, H-3’). ^13^C-NMR (125 MHz, CDCl_3_): δ = 13.2, 18.4, 20.5, 20.7, 41.2, 58.4, 61.9, 68.2, 69.9, 70.8, 72.2, 72.5, 80.8, 99.9, 169.1, 169.3, 170.2, 170.5, 177.3. HRMS (ESI-TOF^+^): *m/z* [M+Na]^+^ calcd for C_19_H_26_O_12_Na: 469.1322; found: 469.1287.

*(2R,3S)-2-molecules-18-01933Allyl-3-(2,3,4,6-tetra-O-acetyl-β-molecules-18-01933D-glucopyranosyloxy)butanolide* (**5c**). White solid, yield 38%. Mp 143–144 °C; [α${]}_{D}^{20}$ −80.3 (c 0.76, CHCl_3_). ^1^H-NMR (300 MHz, CDCl_3_): δ = 2.00~2.08 (m, 12 H, 4 × CH_3_CO), 2.42~2.50 (m, 2 H, CH_2_CH=CH_2_), 2.56~2.61 (m, 1 H, H-2), 3.67~3.70 (d, *J* = 9.3 Hz, 1 H, H-5’), 4.18 (m, 3 H, H-6’a, H-6’b, H-4a), 4.30 (d, *J* = 10.8 Hz, 1 H, H-4b), 4.61 (m, 2 H, H-3, H-1’), 4.98 (dd, *J* = 8.1, 9.0 Hz, 1 H, H-2’), 5.07 (dd, *J* = 9.3, 9.6 Hz, 1 H, H-4’), 5.14~5.24 (m, 3 H, H-3’, CH_2_CH=CH_2_), 5.79~5.93 (m, 1 H, CH_2_CH=CH_2_). ^13^C-NMR(125 MHz, CDCl_3_): δ = 18.4, 20.5, 20.6, 20.7, 27.7, 44.3, 61.7, 68.3, 70.4, 70.9, 72.0, 72.4, 74.1, 98.1, 117.4, 134.5, 169.1, 169.3, 170.3, 170.5, 176.0. HRMS (ESI-TOF^+^): *m/z* [M+Na]^+^ calcd for C_21_H_28_O_12_Na: 495.1478; found: 495.1437.

*(2R,3S)-2-molecules-18-01933Bmolecules-18-01933enzyl-3-(2,3,4,6-tetra-O-acetyl-βmolecules-18-01933-molecules-18-01933Dmolecules-18-01933-glucopyranosyloxy)butanolide* (**5d**). White solid, yield 64%. Mp 202–203 °C; [α${]}_{D}^{20}$ −108.0 (c 1.07, CHCl_3_). ^1^H-NMR (300 MHz, CDCl_3_): δ = 2.03~2.09 (4s, 12 H, 4×CH_3_CO), 2.71~2.78 (m, 1 H, H-2), 3.01 (dd, *J* = 10.5, 13.8 Hz, 1 H, CH_2_Ph), 3.10 (dd, *J* = 3.6, 13.8 Hz, 1 H, CH_2_Ph), 3.63~3.71 (m, 1 H, H-5’), 4.10~4.16 (m, 2 H, H-4a, H-6’a), 4.24 (dd, *J* = 4.5, 12.3 Hz, 1 H, H-6’b), 4.32 (d, *J* = 10.8 Hz, 1 H, H-4b), 4.46~4.51 (m, 1 H, H-3), 4.62 (d, *J* = 8.1 Hz, 1 H, H-1’), 5.08 (dd, *J* = 8.1, 9.0 Hz, 1 H, H-2’), 5.16 (m, 1 H, H-4’), 5.24 (m, 1 H, H-3’), 7.20~7.34 (m, 5 H, Ar-H). ^13^C-NMR (125 MHz, CDCl_3_): δ = 20.4, 20.5, 20.6, 20.7, 29.1, 47.1, 61.9, 68.2, 70.1, 71.0, 72.1, 72.4, 73.9, 98.0, 126.6, 128.4, 128.4, 129.1, 129.1, 138.9, 169.2, 169.3, 170.3, 170.6, 175.7. HRMS (ESI-TOF^+^): *m/z* [M+Na]^+^ calcd for C_25_H_30_O_12_Na: 545.1635; found: 545.1639.

*(2R,3S)-2-(4-molecules-18-01933Mmolecules-18-01933ethoxybenzyl)-3-(2,3,4,6-tetra-O-acetyl-βmolecules-18-01933-molecules-18-01933Dmolecules-18-01933-glucopyranosyloxy)butanolide* (**5e**). White solid, yield 58%. Mp 169–170°C; [α${]}_{D}^{20}$ −116.4 (c 0.87, CHCl_3_). ^1^H-NMR (300 MHz, CDCl_3_): δ = 2.02~2.09 (4s, 12 H, 4 × CH_3_CO), 2.67~2.72 (m, 1 H, H-2), 2.95 (dd, *J* = 10.2, 14.1 Hz, 1 H, CH_2_Ph), 3.52 (dd, *J* = 4.2, 14.1 Hz, 1 H, CH_2_Ph), 3.70 (m, 1 H, H-5’), 3.77 (s, 3 H, OCH_3_), 4.10~4.16 (m, 2 H, H-4a, H-6’a), 4.24 (dd, *J* = 4.2, 12.3 Hz, 1 H, H-6’b), 4.29 (d, *J* = 11.1 Hz, 1 H, H-4b), 4.48 (m, 1 H, H-3), 4.62 (d, *J* = 8.1 Hz, 1 H, H-1’), 5.08 (dd, *J* = 8.1, 9.0 Hz, 1 H, H-2’), 5.15 (t, *J* = 9.3 Hz, 1 H, H-4’), 5.24 (dd, *J* = 9.0, 9.3 Hz, 1 H, H-3’), 6.79 (d, *J* = 8.1 Hz, 2 H, Ar-H), 7.24 (d, *J* = 8.1 Hz, 2 H, Ar-H). ^13^C-NMR (125 MHz, CDCl_3_): δ = 20.5. 20.5, 20.6, 20.7, 32.5, 48.0, 55.2, 61.9, 68.0, 70.9, 71.0, 72.2, 72.4, 76.8, 100.0, 114.2, 114.2, 128.7, 130.3, 130.3, 158.8, 169.0, 169.3, 170.2, 170.5, 176.7. HRMS (ESI-TOF^+^): *m/z* [M+Na]^+^ calcd for C_26_H_32_O_13_Na: 575.1741; found: 575.1750.

### 3.6. General Method for Preparation of Compounds ***6***

To a solution of **3** (1.0 equiv) in absolute EtOH was added NaOEt (1.0 M in EtOH, 0.2 equiv). The mixture was stirred at rt until TLC showed no starting material. Then the solution was neutralized with DOWEX^®^ 50WX-4. The resin was filtered off and the solvent was removed *in vacuo*. The residue and TBAF (1.0 M in THF, 0.5 equiv) were dissolved in THF and stirred at rt until TLC showed no starting material. After removal of the solvent *in vacuo*, the residue was purified by column chromatography to give the products **6**.

*Ethyl (S)-3-(β-molecules-18-01933D-glucopyranosyloxy)-4-hydroxybutanoate* (**6a**). Pale yellow solid, yield 52%. Mp 96–97 °C; [α${]}_{D}^{20}$ −26.32 (c 0.46, CH_3_OH). ^1^H-NMR (300 MHz, CD_3_OD): δ = 1.17 (t, *J* = 7.2 Hz, 3 H, CH_2_CH_3_), 2.50 (dd, *J* = 6.0, 15.9 Hz, 1 H, H-2a), 2.63 (dd, *J* = 7.2, 15.9 Hz, 1 H, H-2b), 3.09 (dd, *J* = 7.6, 9.0 Hz, 1 H, H-2’), 3.16~3.27 (m, 3 H, H-5’, H-4’, H-3’), 3.48~3.60 (m, 3 H, H-4a, H-6’a, H-6’b), 3.74(d, *J* = 10.8 Hz, 1 H, H-4b), 4.05 (q, *J* = 7.2 Hz, 2 H, CH_2_CH_3_), 4.11 (m, 1 H, H-3), 4.34 (d, *J* = 7.5 Hz, 1 H, H-1’). HRMS (ESI-TOF^+^): *m/z* [M+Na]^+^ calcd for C_12_H_22_O_9_Na: 333.1162; found: 333.1132.

*Ethyl (2R,3S)-3-(β-molecules-18-01933D-glucopyranosyloxy)-4-hydroxy-2-methylbutanoate* (**6b**). White solid, yield 77%. Mp 104–106°C; [α${]}_{D}^{20}$ −38.0 (c 0.96, CH_3_OH). ^1^H-NMR (300 MHz, pyridine-*d*_5_): δ = 1.11 (t, *J* = 7.5 Hz, 3 H, CH_2_CH_3_), 1.29 (d, *J* = 6.9 Hz, 3 H, CHCH_3_), 3.15~3.24 (m, 1 H, H-2), 3.90 (br s, 1 H, H-5’), 4.01~4.06 (m, 2 H, H-2’, H-4a), 4.10~4.22 (m, 5 H, H-4b, CH_2_CH_3_, H-4’, H-3’), 4.32 (dd, *J* = 6.0, 12.0 Hz, 1 H, H-6’a), 4.48~4.51 (m, 2 H, H-6’b, H-3), 5.14 (d, *J* = 7.8 Hz, 1 H, H-1’), 5.70 (br s, 5 H, 5×OH). HRMS (ESI-TOF^+^): *m/z* [M+Na]^+^ calcd for C_13_H_24_O_9_Na: 347.1318; found: 347.1305.

*Ethyl (2R,3S)-2-allyl-3-(β-molecules-18-01933D-glucopyranosyloxy)-4-hydroxybutanoate* (**6c**). Pale yellow syrup, yield 69%. [α${]}_{D}^{20}$ −19.96 (c 0.89, EtOH). ^1^H-NMR (300 MHz, CD_3_OD): δ = 1.19 (t, *J* = 7.2 Hz, 3 H, CH_2_CH_3_), 2.24~2.33 (m, 2 H, CH_2_CH=CH_2_), 2.77~2.85 (m, 1 H, H-2), 3.01~3.32 (m, 4 H, H-2’, H-5’, H-4’, H-3’), 3.51~3.61 (m, 2 H, H-4a, H-6’a), 3.70~3.80 (m, 2 H, H-4b, H-6’b), 3.87~3.92 (m, 1 H, H-3), 4.02~4.13 (m, 2 H, CH_2_CH_3_), 4.33 (d, *J* = 7.8 Hz, 1 H, H-1’), 4.98 (dd, *J* = 10.2, 17.1 Hz, 2 H, CH_2_CH=CH_2_), 5.63~5.77 (m, 1 H, CH_2_CH=CH_2_). HRMS (ESI-TOF^+^): *m/z* [M+Na]^+^ calcd for C_15_H_26_O_9_Na: 373.1475; found: 373.1439.

*Ethyl (2R,3S)-2-benzyl-3-(β-molecules-18-01933D-glucopyranosyloxy)-4-hydroxybutanoate* (**6d**). White solid, yield 77%. Mp 140–141°C; [α${]}_{D}^{20}$ +11.13 (c 0.82, CH_3_OH). ^1^H-NMR (300 MHz, pyridine-*d*_5_): δ = 0.96 (t, *J* = 7.2 Hz, 3 H, CH_2_CH_3_), 3.20 (dd, *J* = 10.5, 13.5 Hz, 1 H, CH_2_Ph), 3.31 (dd, *J* = 4.8, 13.5 Hz, 1 H, CH_2_Ph), 3.47~3.54 (m, 1 H, H-2), 3.84~3.89 (m, 1 H, H-5’), 3.95~4.13 (m, 3 H, H-4a, CH_2_CH_3_), 4.16~4.26 (m, 4 H, H-2’, H-4b, H-4’, H-3’), 4.34 (dd, *J* = 5.4, 11.7 Hz, 1 H, H-6’a), 4.49~4.57 (m, 2 H, H-6’b, H-3), 5.15 (d, *J* = 7.8 Hz, 1 H, H-1’), 6.31 (br s, 5 H, 5 × OH), 7.19~7.45 (m, 5 H, Ar-H). HRMS (ESI-TOF^+^): *m/z* [M+Na]^+^ calcd for C_19_H_28_O_9_Na: 423.1631; found: 423.1607.

*Ethyl (2R,3S)-3-(β-molecules-18-01933D-glucopyranosyloxy)-4-hydroxy-2-(4-methoxybenzyl)butanoate* (**6e**). White solid, yield 75%. Mp 150–151°C; [α${]}_{D}^{20}$ +8.0 (c 0.21, CH_3_OH). ^1^H-NMR (300 MHz, CD_3_OD): δ = 0.98 (t, *J* = 7.2 Hz, 3 H, CH_2_CH_3_), 2.65 (dd, *J* = 11.4, 13.2 Hz, 1 H, CH_2_Ph), 2.81 (dd, *J* = 4.8, 13.2 Hz, 1 H, CH_2_Ph), 2.95~3.06 (m, 1 H, H-2), 3.10~3.30 (m, 4 H, H-5’, H-2’, H-4’, H-3’), 3.55 (dd, *J* = 5.1, 11.4 Hz, 1 H, H-4a), 3.61~3.66 (m, 1 H, H-6’a), 3.66 (s, 3 H, OCH_3_), 3.73~3.79 (m, 2 H, H-6’b, H-4b), 3.87~3.94 (m, 3 H, CH_2_CH_3_, H-3), 4.32 (d, *J* = 8.1 Hz, 1 H, H-1’), 6.72 (d, *J* = 8.4 Hz, 2 H, Ar-H), 7.03 (d, *J* = 8.1 Hz, 2 H, Ar-H). HRMS (ESI-TOF^+^): *m/z* [M+Na]^+^ calcd for C_20_H_30_O_10_Na: 453.1737; found: 453.1745.

### 3.7. General Method for Preparation of Compounds ***7***

To a solution of **6** (1.0 equiv.) in 1,4-dioxane was added PTSA·H_2_O (1.0 equiv.). The mixture was stirred at rt until TLC showed no starting material. Then the mixture was concentrated *in vacuo*. The residue was purified by column chromatography to give the products **7**.

*(3S)-3-(βmolecules-18-01933-molecules-18-01933Dmolecules-18-01933-molecules-18-01933Gmolecules-18-01933lucopyranosyloxy)butanolide* (goodyeroside A, **7a**). White solid, yield 63%. Mp 154–155 °C (lit. [4] 156–157°C); [α${]}_{D}^{20}$ −72.7 (c 0.59, H_2_O) (lit. [4] [α${]}_{D}^{20}$ −71.2 (c 0.55, H_2_O)). ^1^H-NMR (500 MHz, pyridine-*d*_5_): δ = 2.86 (dd, *J* = 5.5, 18.0 Hz, 1 H, H-2a), 2.91 (d, *J* = 18.0 Hz, 1 H, H-2b), 3.93 (br s, 1 H, H-5’), 3.97 (m, 1 H, H-2’), 4.18~4.23 (m, 2 H, H-3’, H-4’), 4.34 (m, 1 H, H-6’a), 4.35 (dd, *J* = 4.5, 10.0 Hz, 1 H, H-4a), 4.54 (dd, *J* = 1.5, 11.5 Hz, 1 H, H-6’b), 4.63 (d, *J* = 10.0 Hz, 1 H, H-4b), 4.86 (m, 1 H, H-3), 4.92 (d, *J* = 7.5 Hz, 1 H, H-1’). ^13^C-NMR (125 MHz, pyridine-*d*_5_): δ = 36.4, 62.7, 71.4, 74.0, 74.6, 74.7, 78.3, 78.7, 103.6, 176.3. HRMS (ESI-TOF^+^): *m/z* [M+Na]^+^ calcd for C_10_H_16_O_8_Na: 287.0743; found: 287.0750.

*(2R,3S)-3-(βmolecules-18-01933-molecules-18-01933Dmolecules-18-01933-Glucopyranosyloxy)-2-methylbutanolide* (**7b**). White solid, yield 77%. Mp 126–127 °C; [α${]}_{D}^{20}$ −68.69 (c 0.98, CH_3_OH). ^1^H-NMR (300 MHz, pyridine-*d*_5_): δ = 1.30 (d, *J* = 6.9 Hz, 3 H, CHCH_3_), 2.78~2.90 (m, 1 H, H-2), 3.94~3.99 (m, 2 H, H-5’, H-2’), 4.15~4.27 (m, 3 H, H-3’, H-4’, H-4a), 4.36 (dd, *J* = 8.7, 12.0 Hz, 1 H, H-6’a), 4.58 (d, *J* = 12.0 Hz, 1 H, H-6’b), 4.72 (d, *J* = 10.5 Hz, 1 H, H-4b), 4.84 (m, 1 H, H-3), 4.91 (d, *J* = 7.5 Hz, 1 H, H-1’), 6.13 (br s, 4 H, 4 × OH). ^13^C-NMR (100 MHz, pyridine-*d*_5_): δ = 9.1, 39.3, 62.8, 71.4, 72.1, 74.5, 76.0, 78.4, 78.8, 102.7, 178.9. HRMS (ESI-TOF^+^): *m/z* [M+Na]^+^ calcd for C_11_H_18_O_8_Na: 301.0899; found: 301.0866.

*(2R,3S)-2-molecules-18-01933Allyl-3-(β-molecules-18-01933D-glucopyranosyloxy)butanolide* (**7c**). Colorless oil, yield 58%. [α${]}_{D}^{20}$ −67.84 (c 3.36, CH_3_OH). ^1^H-NMR (300 MHz, CD_3_OD): δ = 2.25~2.40 (m, 2 H, CH_2_CH=CH_2_), 2.68 (m, 1 H, H-2), 3.06~3.22 (m, 4 H, H-5’, H-2’, H-4’, H-3’), 3.51~3.58 (m, 1 H, H-6’a), 3.80 (d, *J* = 11.1 Hz, 1 H, H-6’b), 4.18~4.22 (m, 1 H, H-4a), 4.29 (d, *J* = 7.2 Hz, 1 H, H-1’), 4.39 (d, *J* = 10.2 Hz, 1 H, H-4b), 4.68 (m, 1 H, H-3), 5.02 (dd, *J* = 10.8, 16.8 Hz, 2 H, CH_2_CH=CH_2_), 5.94 (m, 1 H, CH_2_CH=CH_2_). ^13^C-NMR(125 MHz, pyridine-*d*_5_): δ = 28.5, 44.6, 62.9, 71.6, 72.4, 74.6, 75.2, 78.4, 78.7, 102.8, 116.7, 136.5, 177.6. HRMS (ESI-TOF^+^): *m/z* [M+Na]^+^ calcd for C_13_H_20_O_8_Na: 327.1050; found: 327.1057.

*(2R,3S)-2-molecules-18-01933Benzyl-3-(β-molecules-18-01933D-glucopyranosyloxy)butanolide* (**7d**). White solid, yield 85%. Mp 150–151 °C; [α${]}_{D}^{20}$ −120.44 (c 0.36, CH_3_OH). ^1^H-NMR (600 MHz, pyridine-*d*_5_): δ = 3.02~3.06 (m, 1 H, H-2), 3.26 (dd, *J* = 3.6, 13.8 Hz, 1 H, CH_2_Ph), 3.34 (dd, *J* = 10.2, 13.8 Hz, 1 H, CH_2_Ph), 3.91~3.93 (m, 1 H, H-5’), 4.02 (dd, *J* = 7.8, 8.4 Hz, 1 H, H-2’), 4.20~4.25 (m, 3 H, H-4’, H-3’, H-4a), 4.38 (dd, *J* = 5.4, 12.0 Hz, 1 H, H-6’a), 4.56 (dd, *J* = 2.4, 12.0 Hz, 1 H, H-6’b), 4.73~4.74 (m, 1 H, H-3), 4.82 (d, *J* = 10.2 Hz, 1 H, H-4b), 4.90 (d, *J* = 7.2 Hz, 1 H, H-1’), 6.32 (br s, 4 H, 4 × OH), 7.24 (m, 1 H, Ar-H), 7.41 (m, 2 H, Ar-H), 7.64 (m, 2 H, Ar-H). ^13^C-NMR (125 MHz, pyridine-*d*_5_): δ = 30.0, 47.4, 63.1, 64.5, 71.6, 72.3, 74.6, 75.1, 78.4, 78.6, 103.0, 126.7, 128.8, 128.8, 129.9, 140.4, 177.4. HRMS (ESI-TOF^+^): *m/z* [M+Na]^+^ calcd for C_17_H_22_O_8_Na: 377.1212; found: 377.1214.

*(2R,3S)-3-(β-molecules-18-01933D-Glucopyranosyloxy)-2-(4-methoxybenzyl)butanolide* (**7e**). White solid, yield 94%. Mp 186–187 °C; [α${]}_{D}^{20}$ −110.64 (c 0.38, CH_3_OH). ^1^H-NMR (300 MHz, pyridine-*d*_5_): δ = 3.00~3.07 (m, 1 H, H-2), 3.23 (dd, *J* = 3.9, 14.1 Hz, 1 H, CH_2_Ph), 3.32 (dd, *J* = 9.6, 14.1 Hz, 1 H, CH_2_Ph), 3.64 (s, 3 H, OCH_3_), 3.94 (m, 1 H, H-5’), 3.98~4.03 (m, 1 H, H-2’), 4.19~4.24 (m, 3 H, H-4’, H-3’, H-4a), 4.39 (dd, *J* = 5.1, 11.4 Hz, 1 H, H-6’a), 4.57 (d, *J* = 11.4 Hz, 1 H, H-6’b), 4.77~4.80 (m, 1 H, H-3), 4.83 (d, *J* = 10.2 Hz, 1 H, H-4b), 4.92 (d, *J* = 7.5 Hz, 1 H, H-1’), 7.07 (d, *J* = 8.1 Hz, 2 H, Ar-H), 7.61 (d, *J* = 8.7 Hz, 2 H, Ar-H). ^13^C-NMR (125 MHz, pyridine-*d*_5_): δ = 29.2, 47.6, 55.1, 63.1, 64.5, 71.7, 72.3, 74.7, 75.2, 78.4, 78.6, 103.0, 109.7, 114.4, 131.0, 132.4, 158.8, 177.4. HRMS (ESI-TOF^+^): *m/z* [M+Na]^+^ calcd for C_18_H_24_O_9_Na: 407.1318; found: 407.1357.

### 3.8. General Method for Preparation of Compound ***5d*** from Compound ***7d***

A solution of **7d** in Ac_2_O and dry pyridine was stirred at rt overnight. Then the reaction was quenched with the aqueous CuSO_4_ and extracted with ethyl acetate. The combined organic layer was washed with 0.05 M HCl and saturated aqueous NaCl, dried over Na_2_SO_4_. The solution was filtered and concentrated *in vacuo*. The residue was purified by column chromatography to give the product **5d** as a white solid in 95% yield.

### 3.9. Bioassay Methods

#### 3.9.1. Hepatoprotective Activities against D-Galactosamine-Induced Cytotoxicity in WB-F344 Cells

The hepatoprotective activities were determined using a 3-(4,5-dimethylthiazol-2-yl)-2,5-diphenyltetrazolium bromide (MTT) colorimetric assay in WB-F344 cells. Each cell suspension of 1 × 10^4^ cells in 200 μL of Dulbecco’s modified Eagle’s medium containing fetal calf serum (3%), penicillin (100 units/mL), and streptomycin (100 μg/mL) was planted in a 96-well microplate and precultured for 24 h at 37 °C under a 5% CO_2_ atmosphere. Fresh medium (200 μL) containing the positive control bicyclol (purity >99%, Beijing Union Pharmaceutical Factory, Beijing, China) or test samples were added. After incubation for 1 h, the cells were exposed to 40 mM D-galactosamine for further 24 h. The cytotoxic effects of test samples were simultaneously measured in the absence of D-galactosamine. The medium was replaced by 0.5 mg/mL MTT. After 3.5 h incubation, the medium was removed, and 150 μL of DMSO was added to dissolve the formazan crystals. The optical density (OD) of the formazan solution was measured at 492 nm using a microplate reader.

#### 3.9.2. Determination of Serum ALT and AST levels

Male ICR mice weighing 18–20 g were purchased from the Beijing Weitonglihua Experimental Animal Co., Ltd. (Beijing, China). All mice were maintained under controlled conditions and had free access to standard laboratory chow and water. Animal care and all experimental procedures were conducted in accordance with the health criteria for care of laboratory animals enacted by the Beijing municipal government.

The mice were randomly divided into five groups (seven mice/group). The test compounds suspended in 0.5% CMC-Na were administered orally at a dose of 158 mg/kg (**5a**), 110 mg/kg (**7a**) and 200 mg/kg (bicyclol) at 1, 8 and 24 h before ConA injection, while control mice received an equal volume of vehicle alone. ConA at a dose of 26 mg/kg was injected intravenously to mice except normal group. Blood samples were collected at 16 h after ConA treatment. The serum was obtained by centrifugation at 4000 rpm for 5 min. The levels of ALT and AST were determined using the appropriate commercial kits (Beijing BHKT Clinical Reagent, Beijing, China).

## 4. Conclusions

A highly stereoselective synthetic route of goodyeroside A and its analogs starting from (*S*)-malic acid has been developed. The absolute configuration of analogs **5** and **7** were confirmed to be the 2*R*, 3*S*-configuration via X-ray crystal analysis. Many of the analogs demonstrated hepatoprotective activity in the *in vitro* GalN-induced hepatocyte injury model while compounds **5a** and **7a** exhibited protection comparable to bicyclol. Further *in vivo* studies demonstrated that the fully acetylated product **5a** significantly decreased the serum levels of ALT and AST in ConA-induced acute liver injury in mice. Preliminary results from the SAR studies on goodyeroside A and its analogs establish compound **5a** as our new lead for future molecular design and development of a novel hepato-protective agent.

## Figures and Tables

**a**: R = H; **b**: R = CH_3_-; **c**: R = CH_2_=CHCH_2_-; **d**: R = C_6_H_5_CH_2_-; **e**: R = *p*-MeOC_6_H_4_CH_2_-.

*Rmolecules-18-01933eagents and Conditions*: (i) LiHMDS, DMI, RX, THF, −45 °C; (ii) 2,3,4,6-tetra-*O*-acetyl-α-D-glucopyranosyl trichloroacetimidate, 4Å MS, TMSOTf, CH_2_Cl_2_, −20 °C~rt; (iii) TBAF/THF, rt; (iv) PTSA·H_2_O, 1,4-dioxane, rt; (v) (a): NaOEt/EtOH, rt; (b):TBAF/THF, rt; (vi) PTSA·H_2_O, 1, 4-dioxane, rt.

Compared with Control, ^###^ *p* < 0.001; Compared with GalN, * *p* < 0.05, ** *p* < 0.01.

**Table 1:** Effects of Bicyclol, **5a** and **7a** on ConA-induced liver injury in mice

Group	ALT(U/dL)	AST(U/dL)
Normal	21.0 ± 4.1 **	89.8 ± 4.9 **
Untreated	612.1 ± 263.4	510.7 ± 120.9
Bicyclol	214.2 ± 123.1 **	334.7 ± 122.5 *
**5a**	164.2 ± 75.1 **	243.7 ± 30.7 **
**7a**	615.4 ± 412.1	nt

* *p* < 0.05, ** *p* < 0.01 *vs*. controls. Bicyclol 200 mg/Kg, **5a** 158 mg/Kg, **7a** 110 mg/Kg orally. nt: not tested.

## Supplementary Materials

Supplementary materials can be accessed at: http://www.mdpi.com/1420-3049/18/2/1933/s1.
