# Supplementary material for: Design, Synthesis and Hepatoprotective Activity of Analogs of the Natural Product Goodyeroside A
Source: Molecules. 2013 Feb 1;18(2):1933–48. doi: 10.3390/molecules18021933 (PMC6269934; doi:10.3390/molecules18021933)

Supplementary Materials

Figure 1. ^{1}H-NMR of compound **2b**.

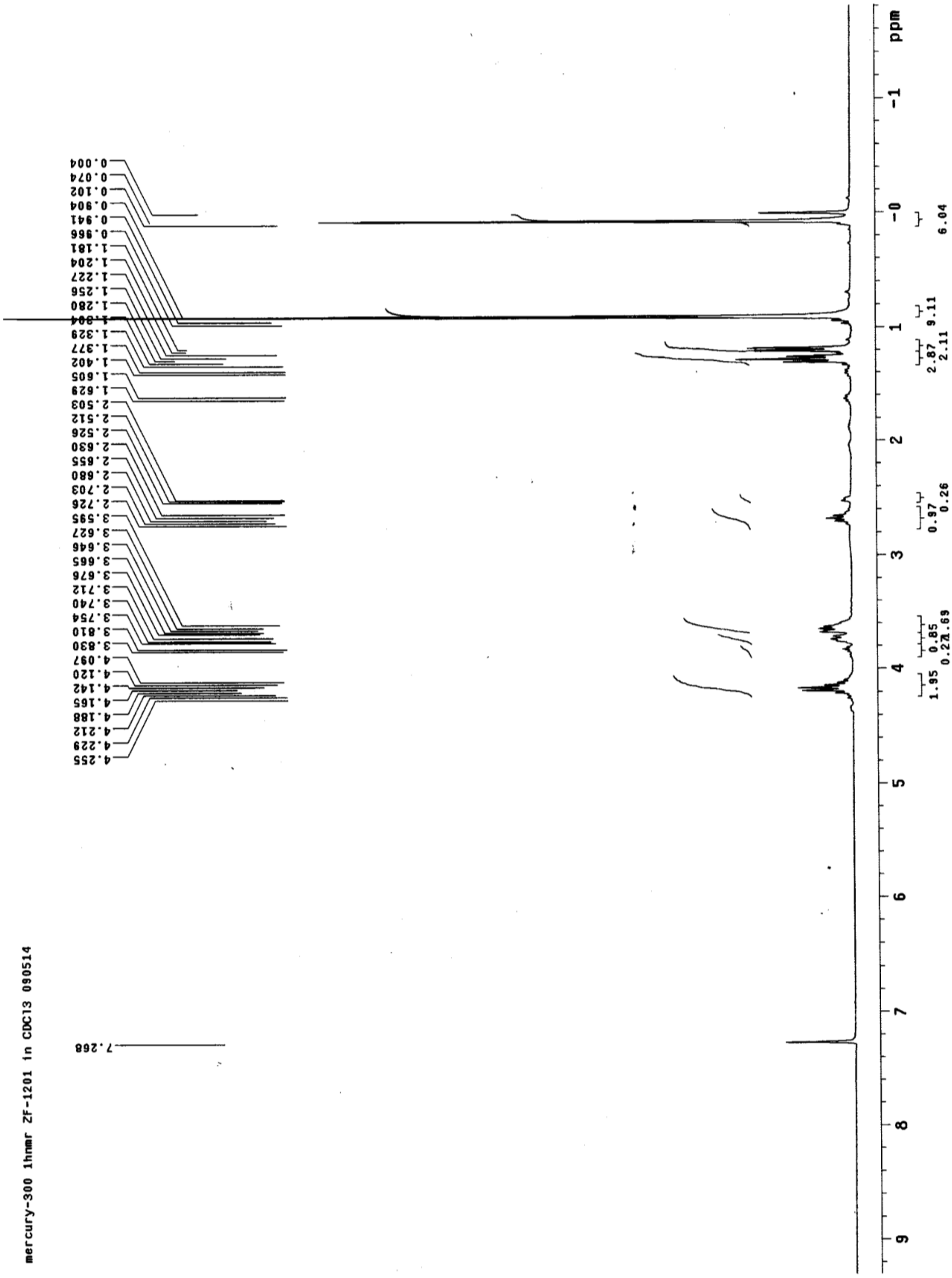

Figure 2. HRMS of compound **2b**.

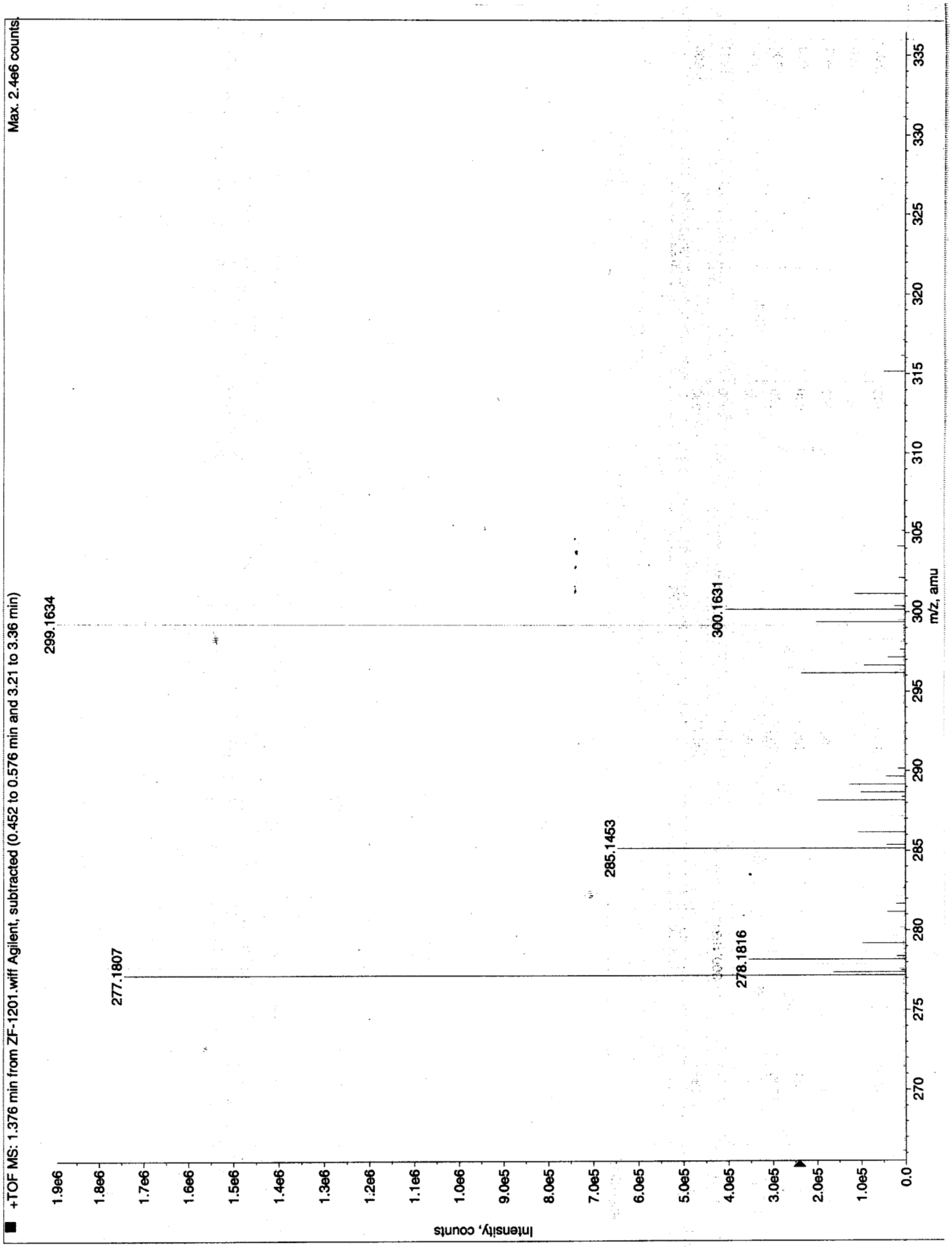

Figure 3. ¹H-NMR of compound **2c**.

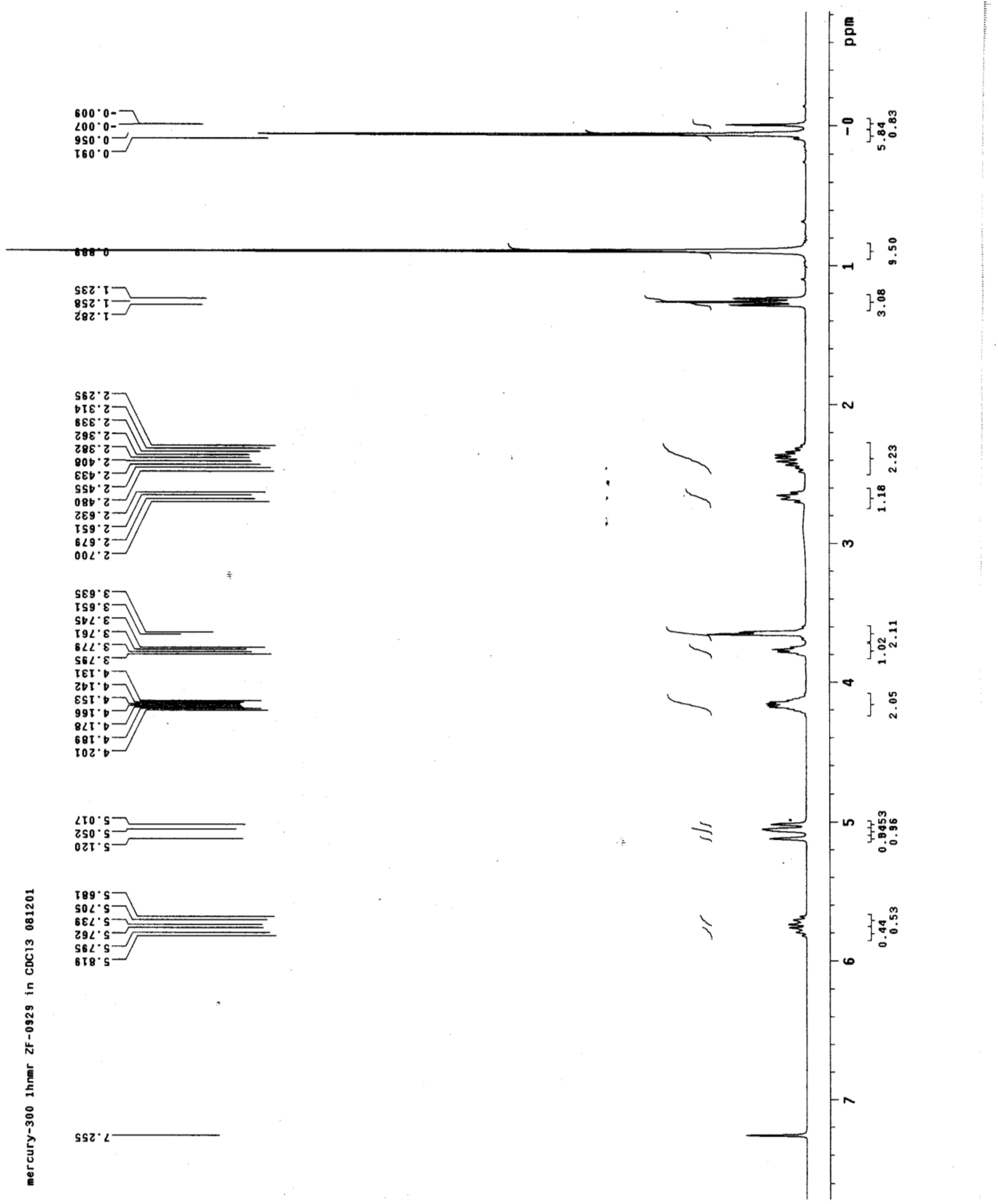

Figure 4. HRMS of compound **2c**.

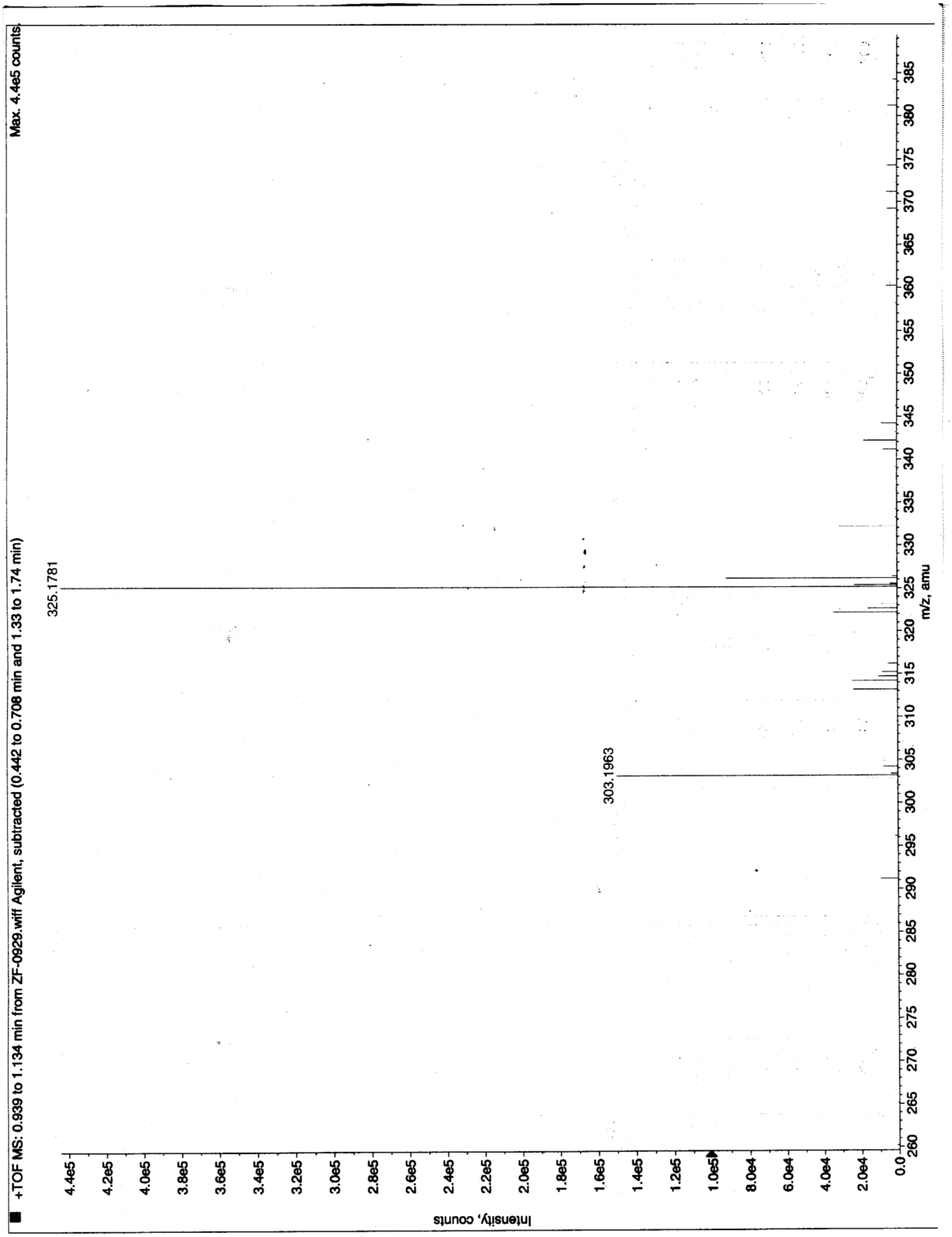

Figure 5. ^{1}H-NMR of compound **2d**.

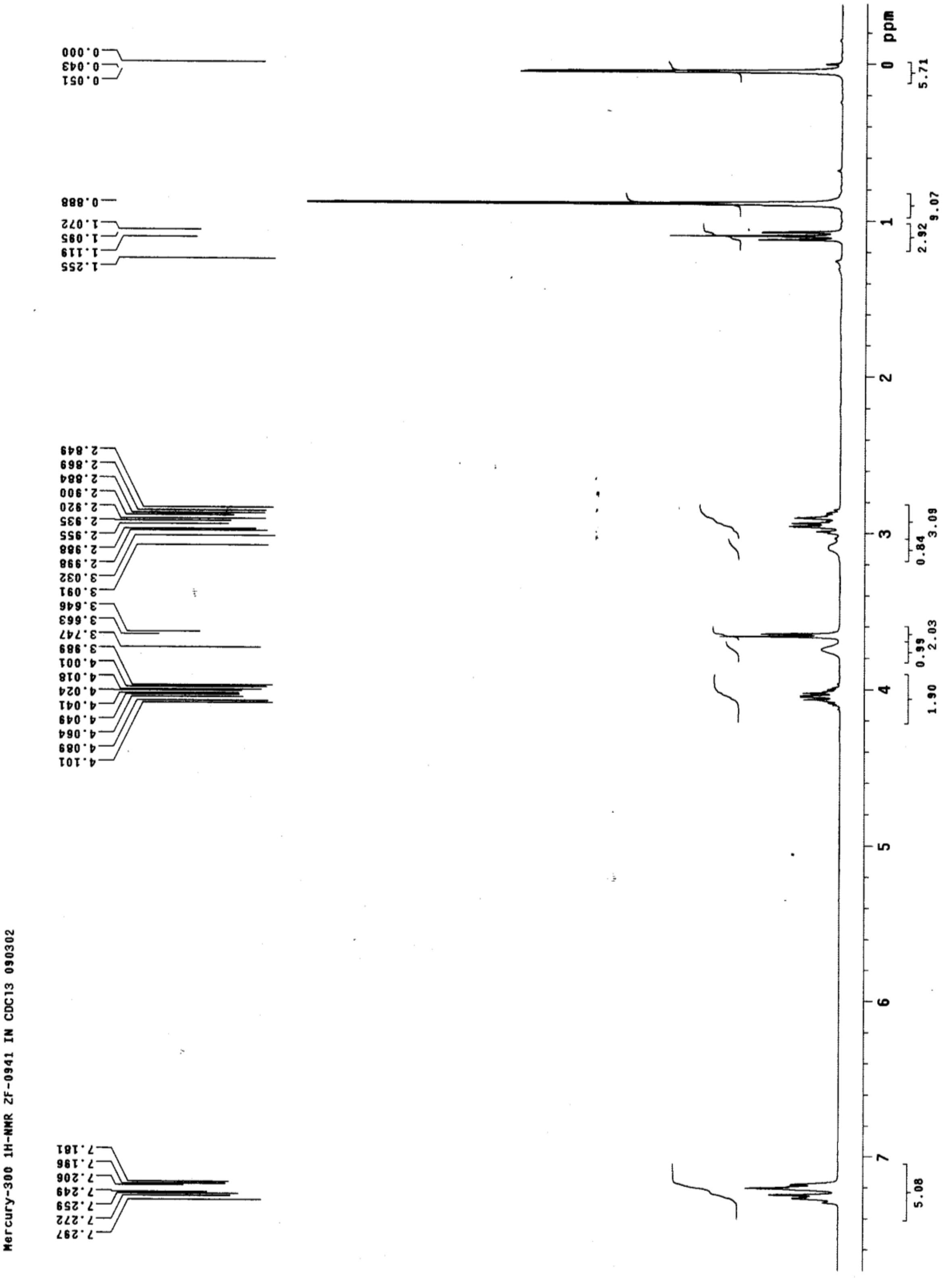

Figure 6. HRMS of compound **2d**.

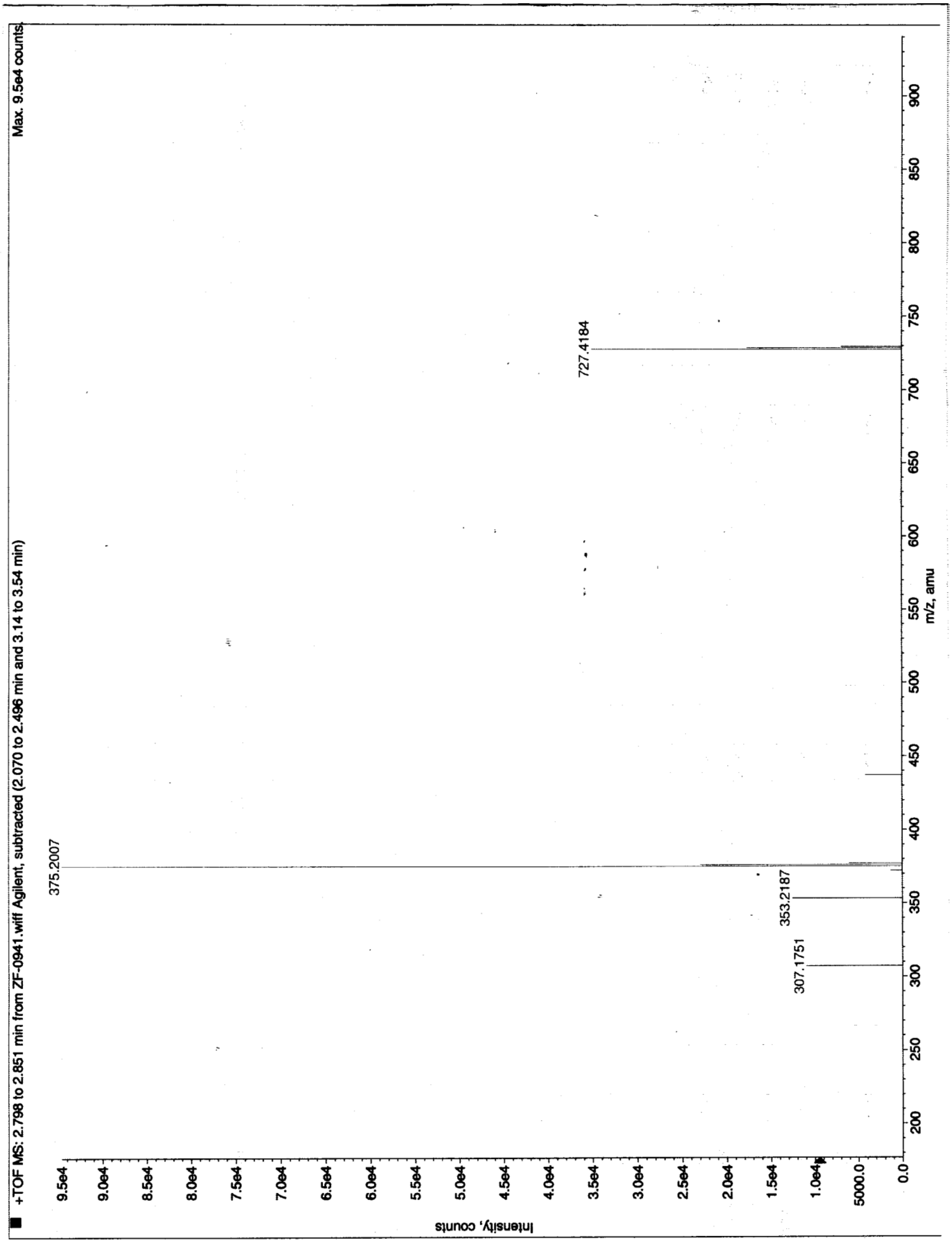

Figure 7. ^{1}H-NMR of compound **2e**.

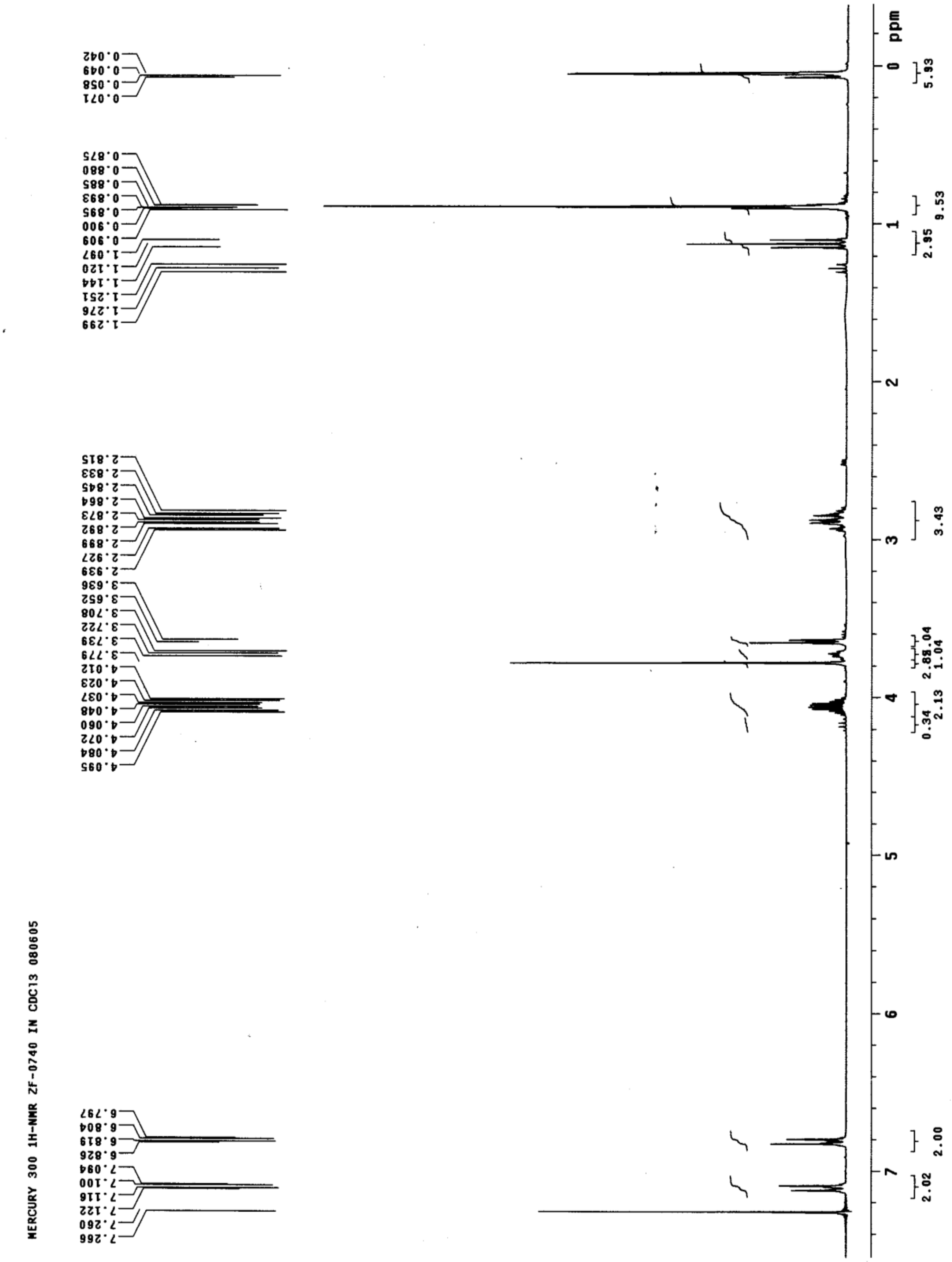

Figure 8. HRMS of compound **2e**.

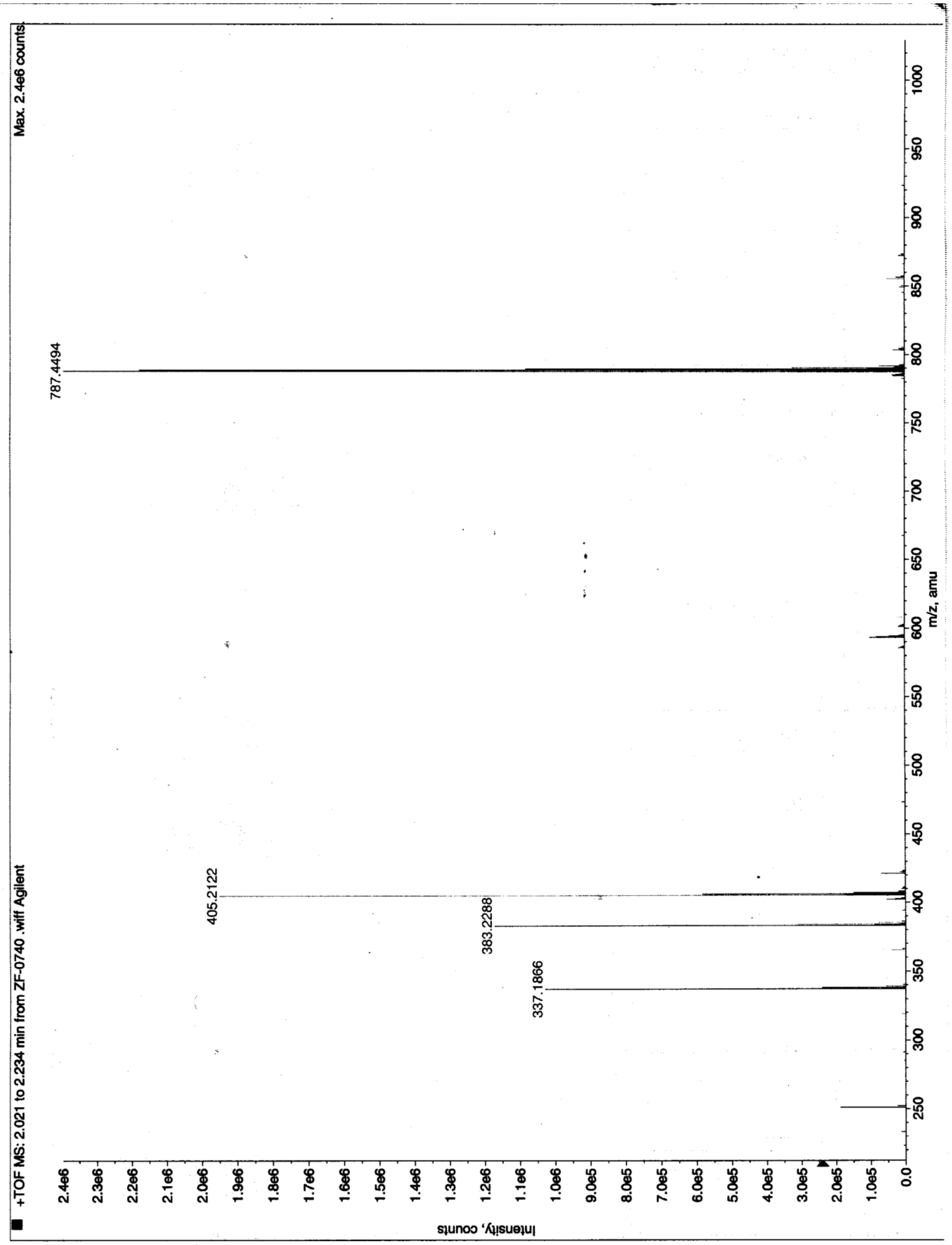

Figure 9. ^{1}H-NMR of compound **3a**.

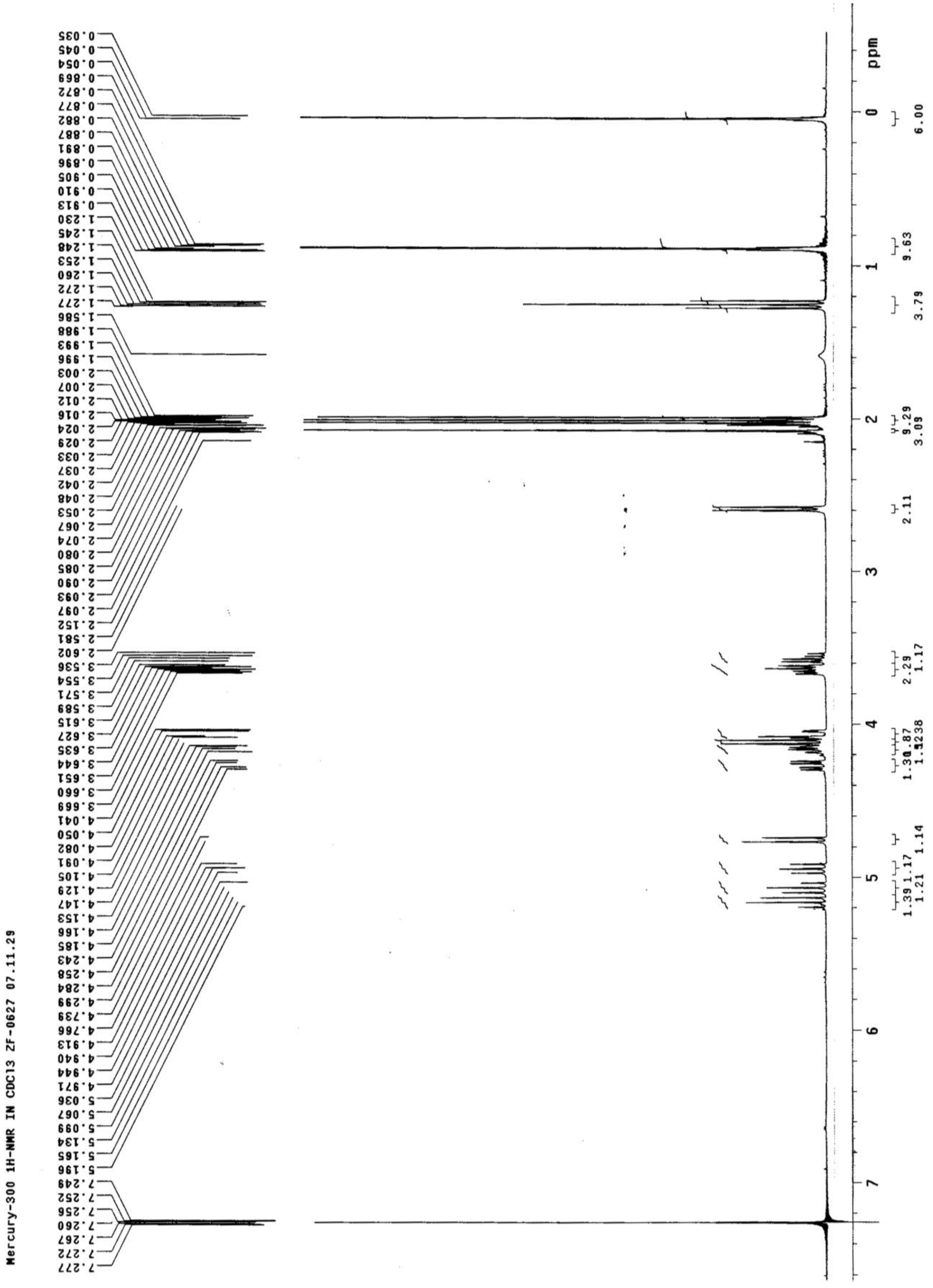

Figure 10. HRMS of compound **3a**.

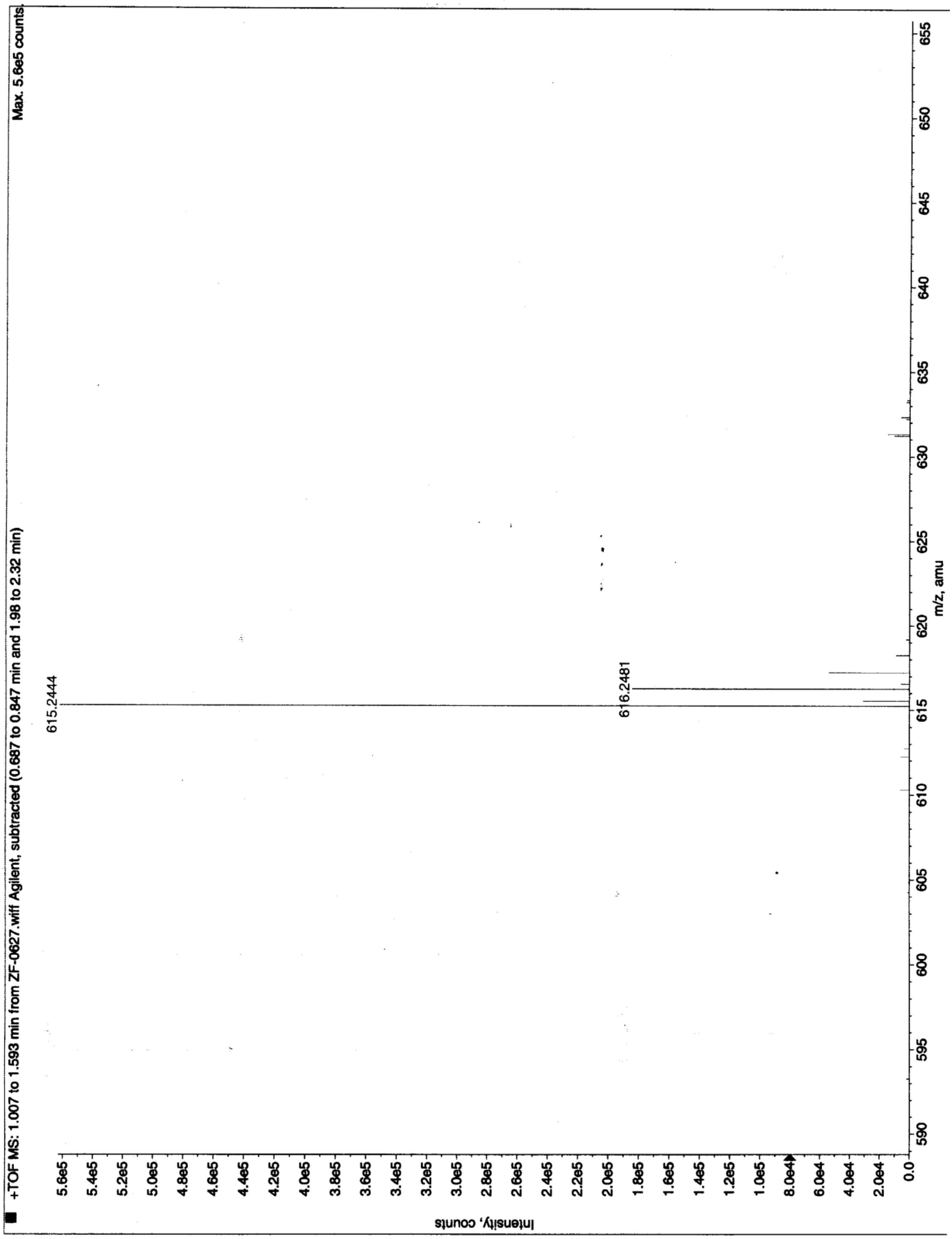

Figure 11. ^{1}H-NMR of compound **3b**.

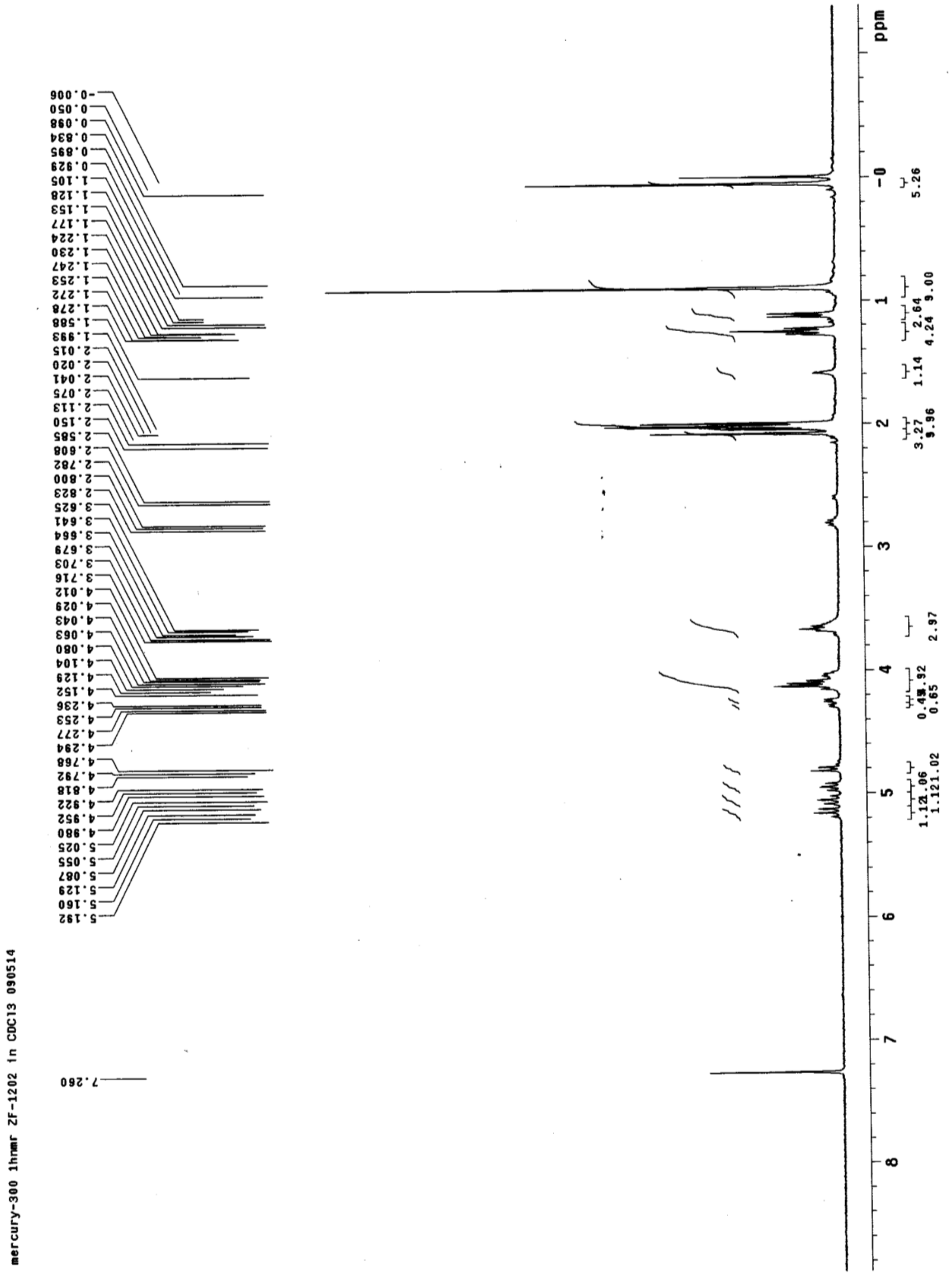

Figure 12. HRMS of compound **3b**.

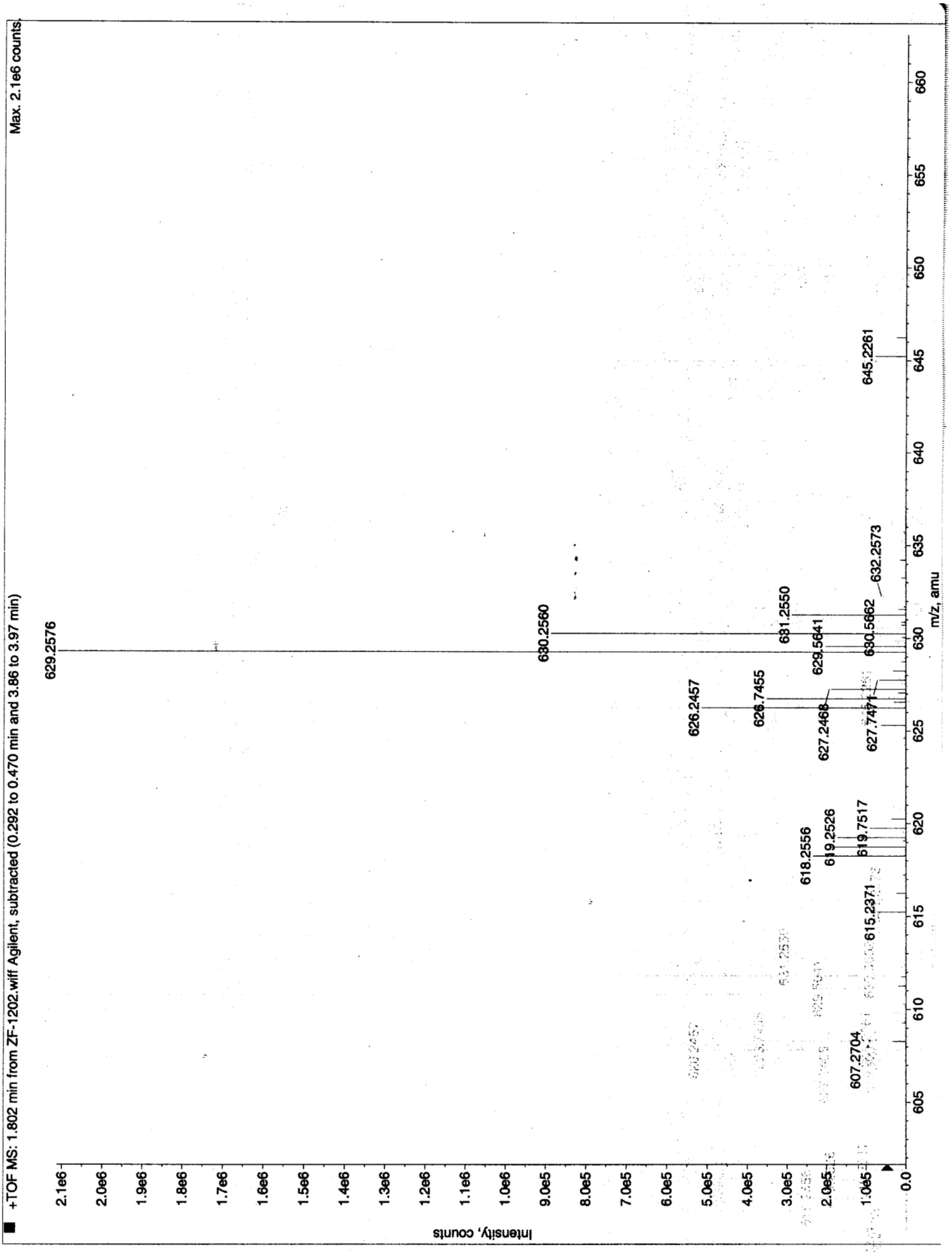

Figure 13. ^{1}H-NMR of compound **3c**.

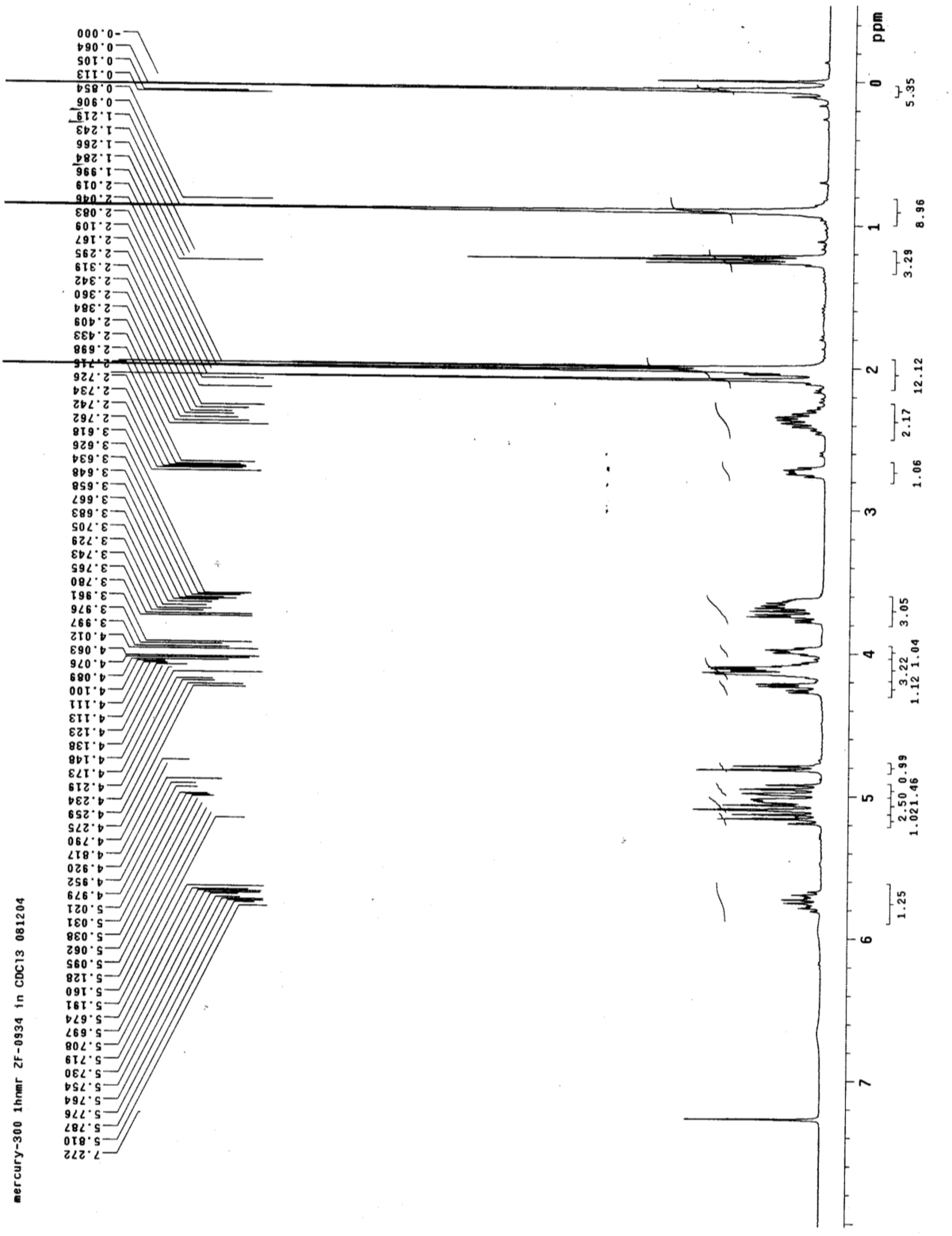

Figure 14. HRMS of compound **3c**.

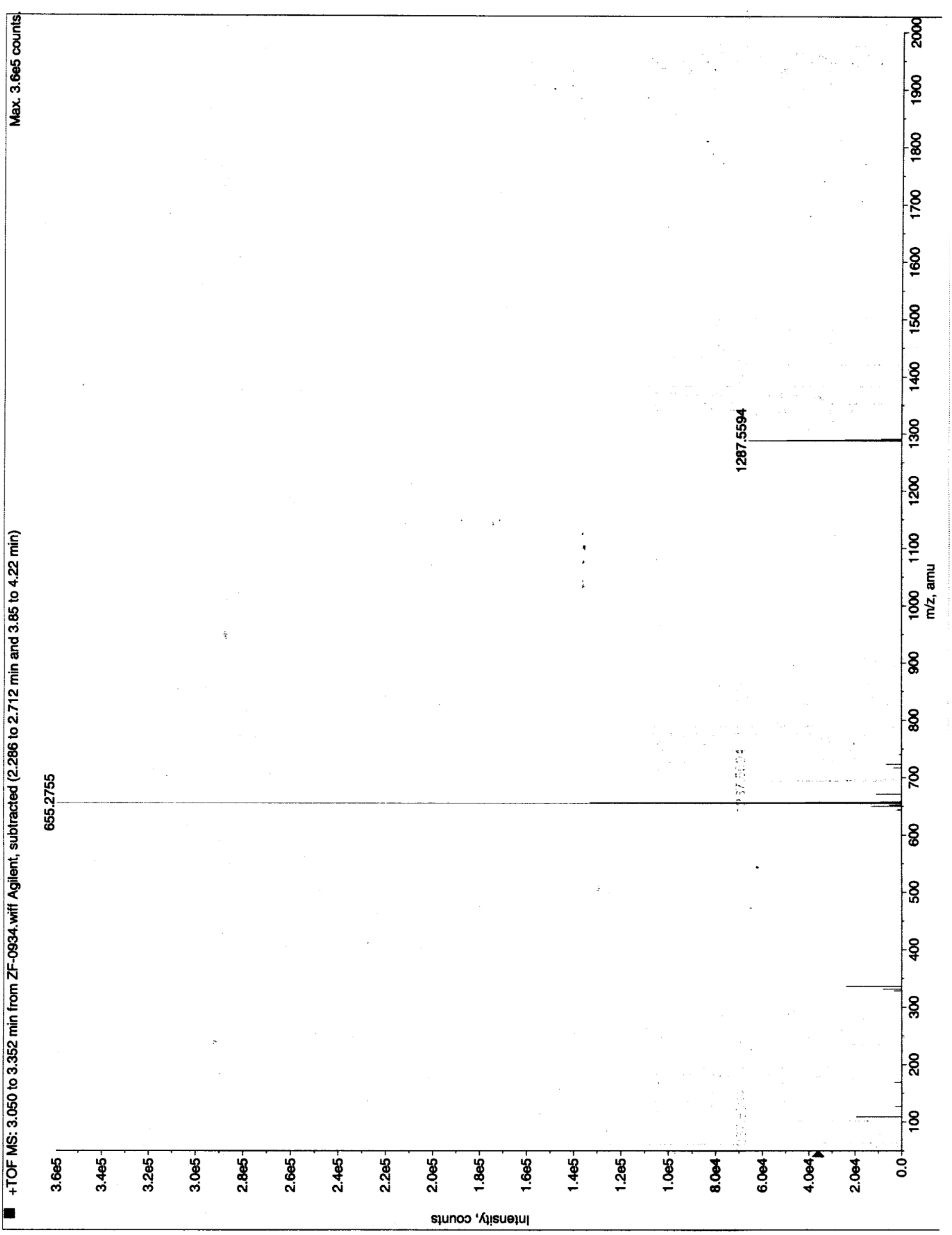

Figure 15. ^{1}H-NMR of compound **3d**.

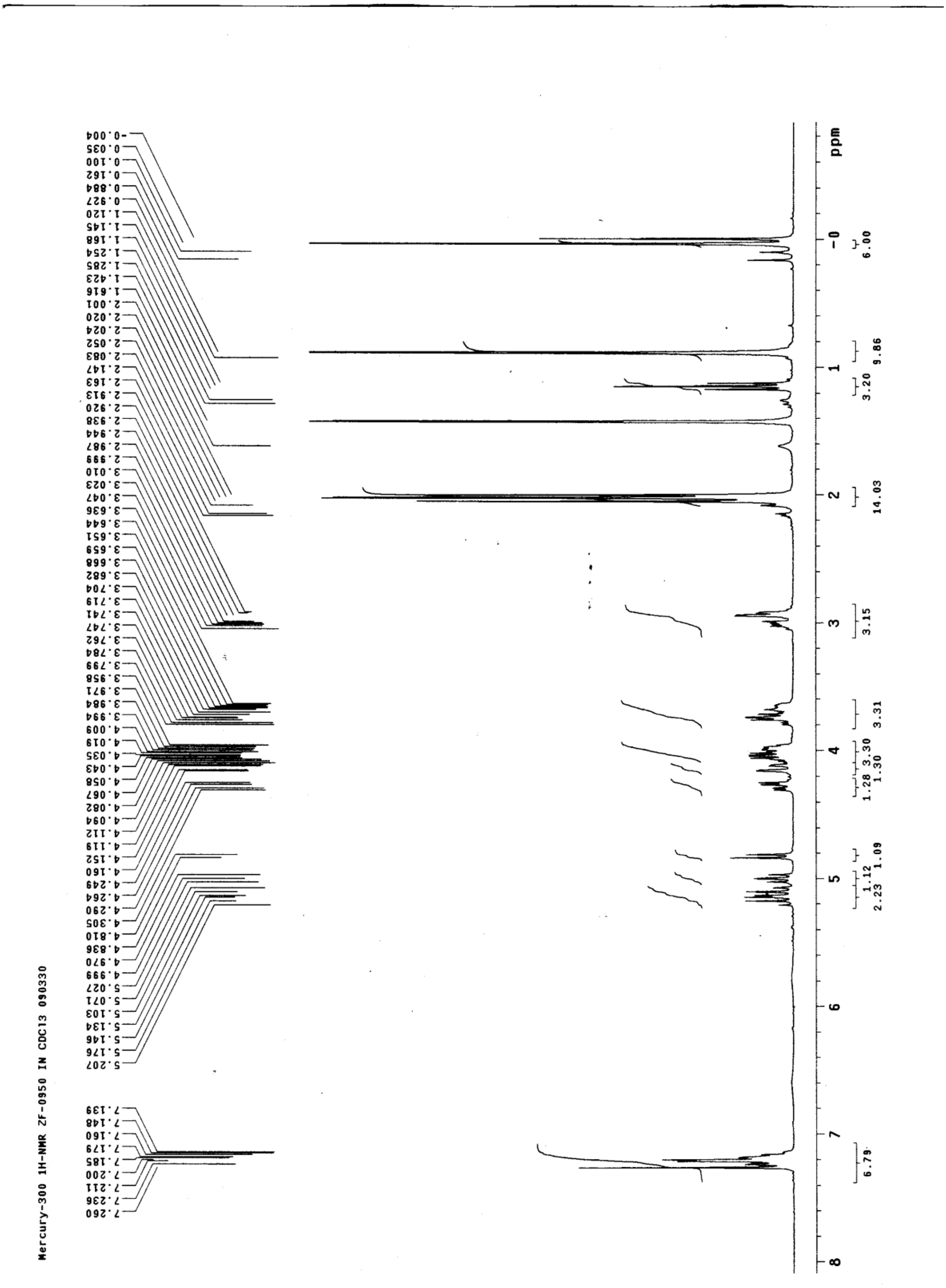

Figure 16. HRMS of compound 3d.

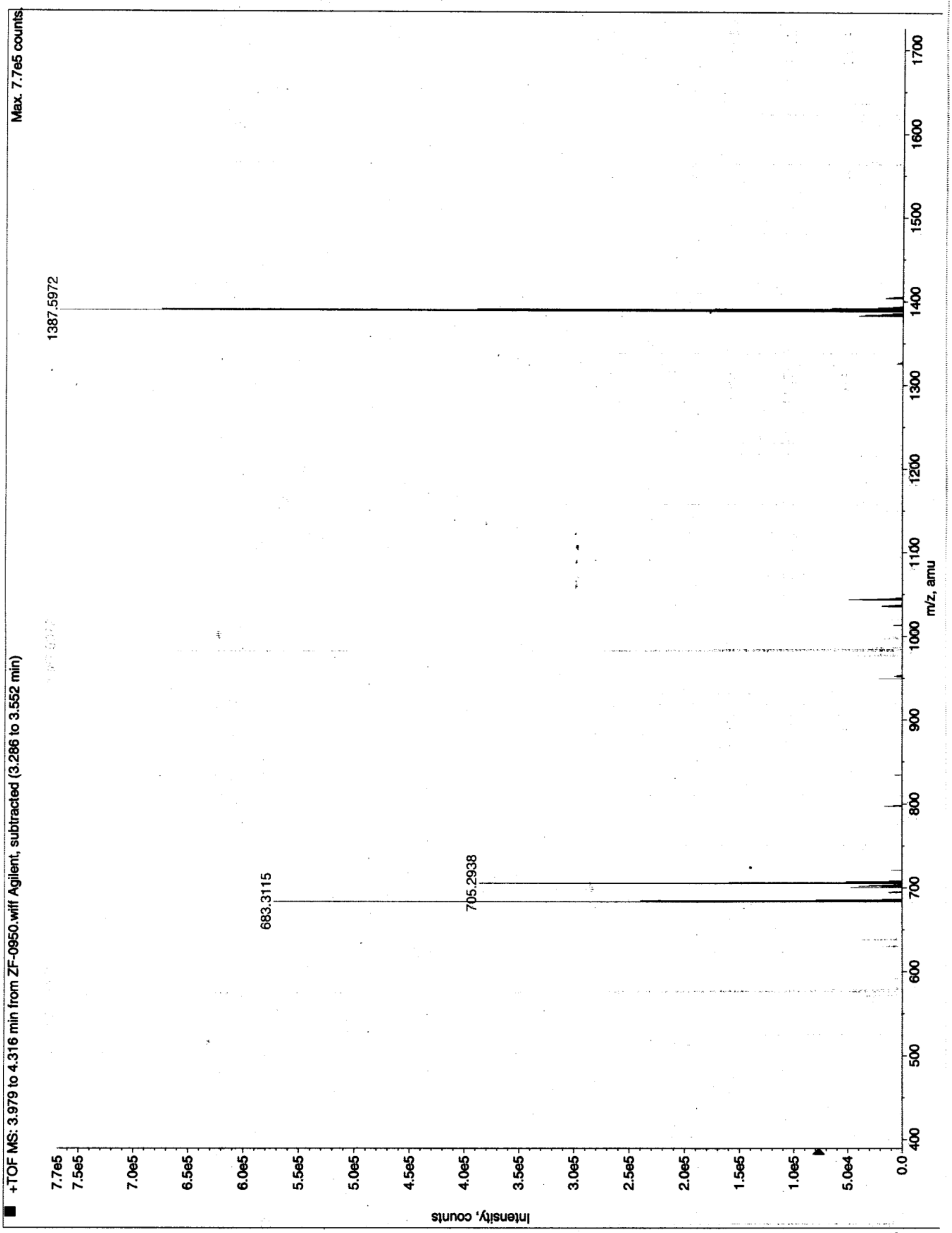
+TOF MS: 3.979 to 4.316 min from ZF-0950.wiff Agilent, subtracted (3.286 to 3.552 min)
Max. 7.7e5 counts.
1387.5972
705.2938
683.3115
Intensity, counts
m/z, amu

Figure 17. ^{1}H-NMR of compound **3e**.

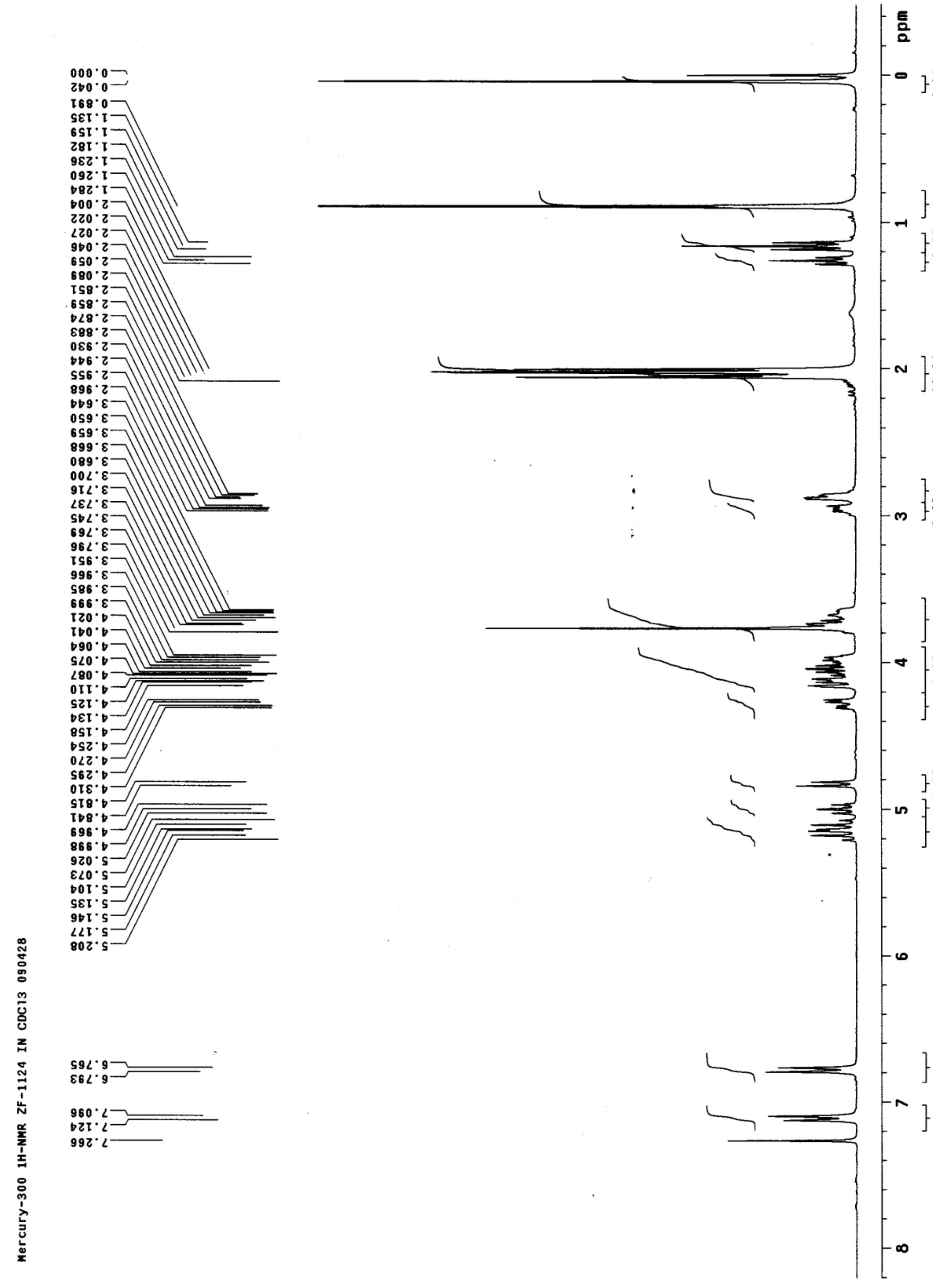

Figure 18. HRMS of compound **3e**.

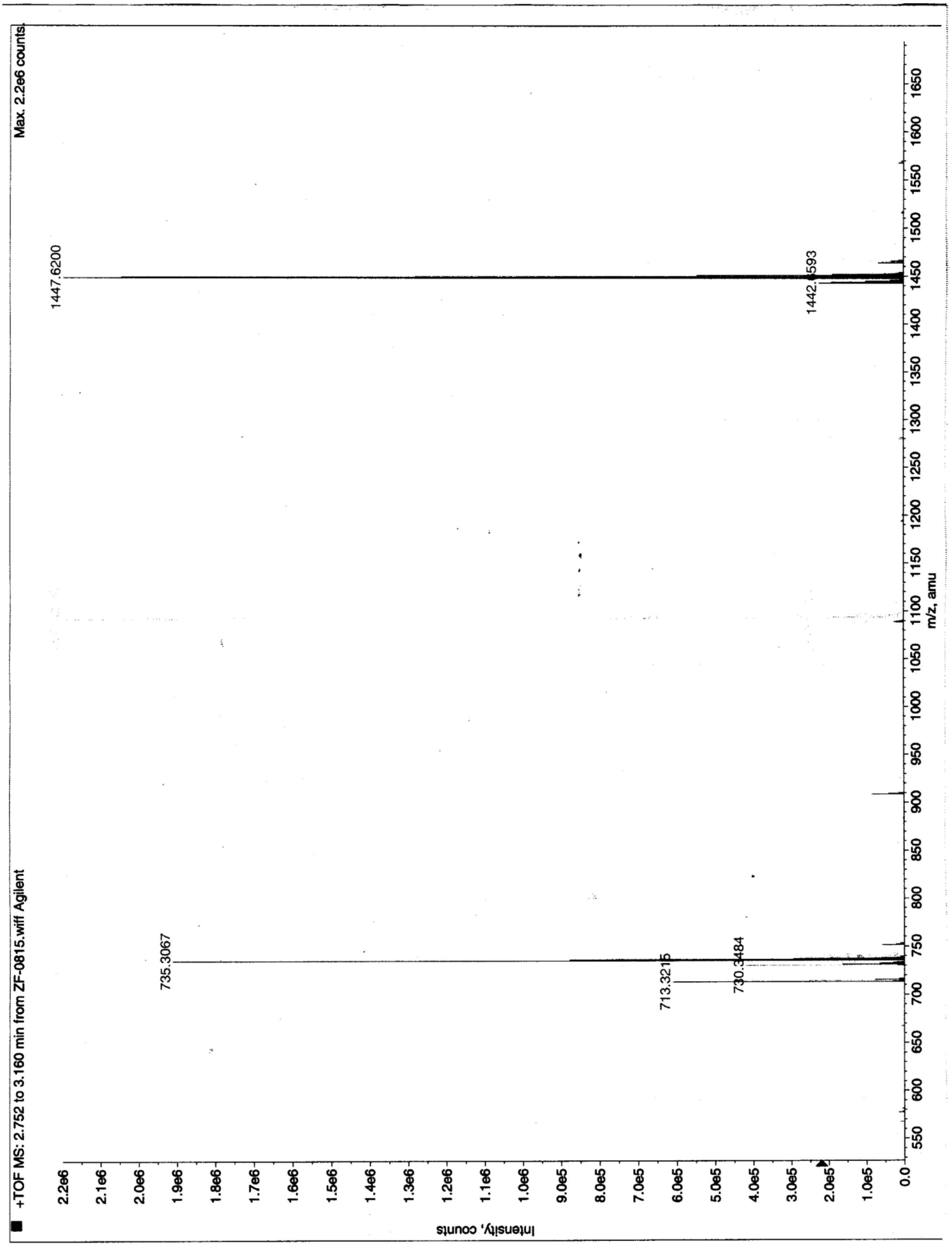

Figure 19. ^{1}H-NMR of compound **4a**.

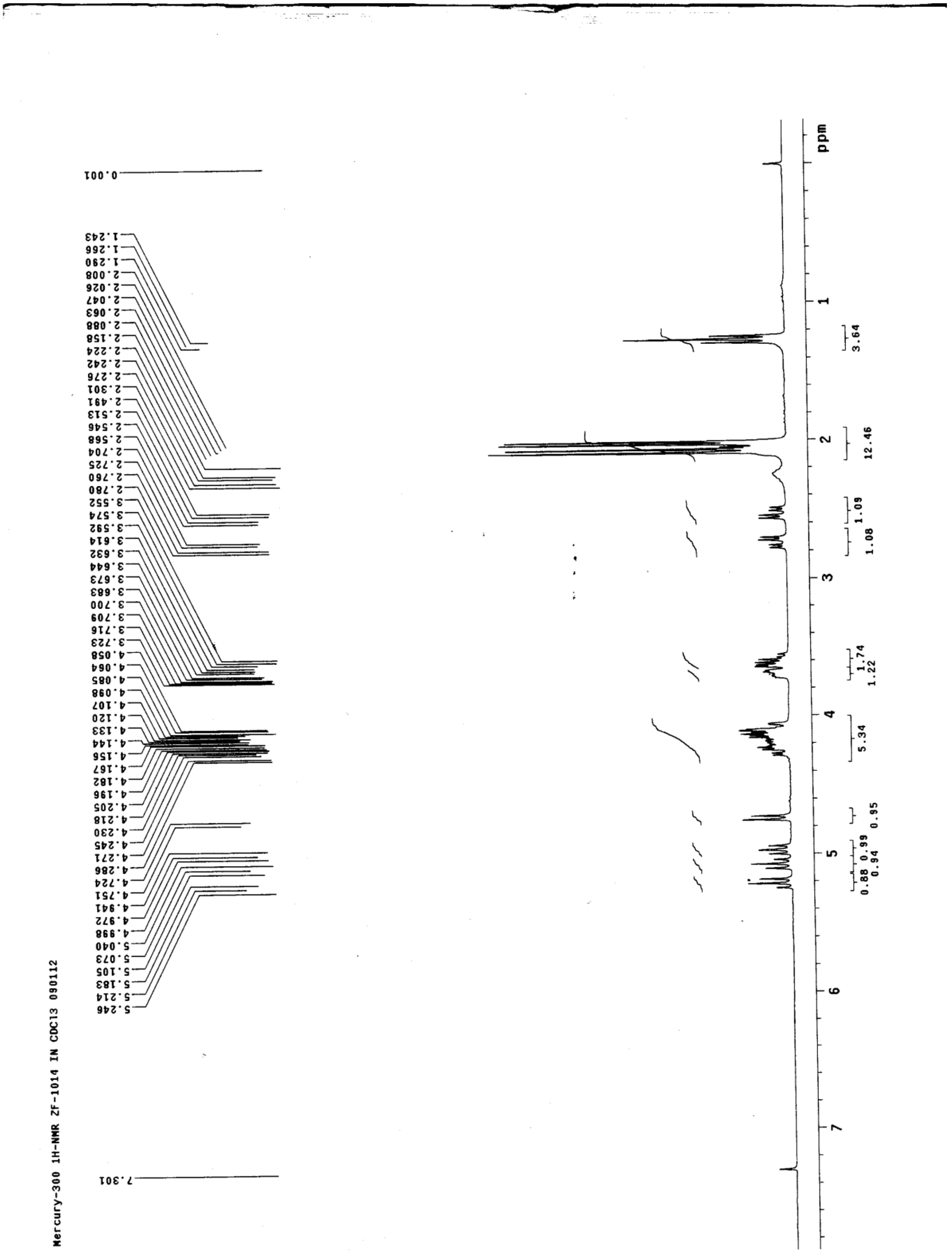

Figure 20. HRMS of compound **4a**.

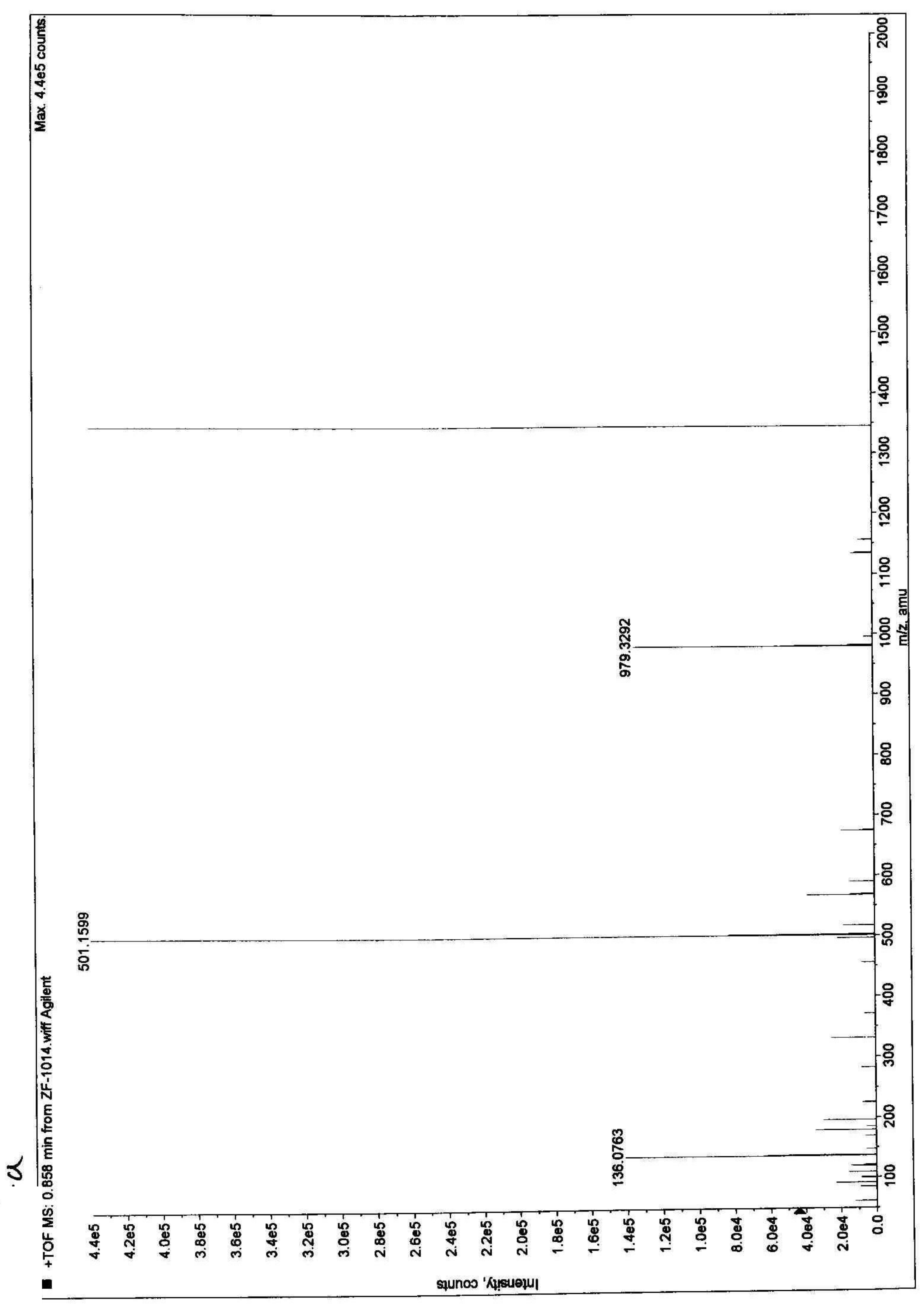

Figure 21. ^{1}H-NMR of compound **4b**.

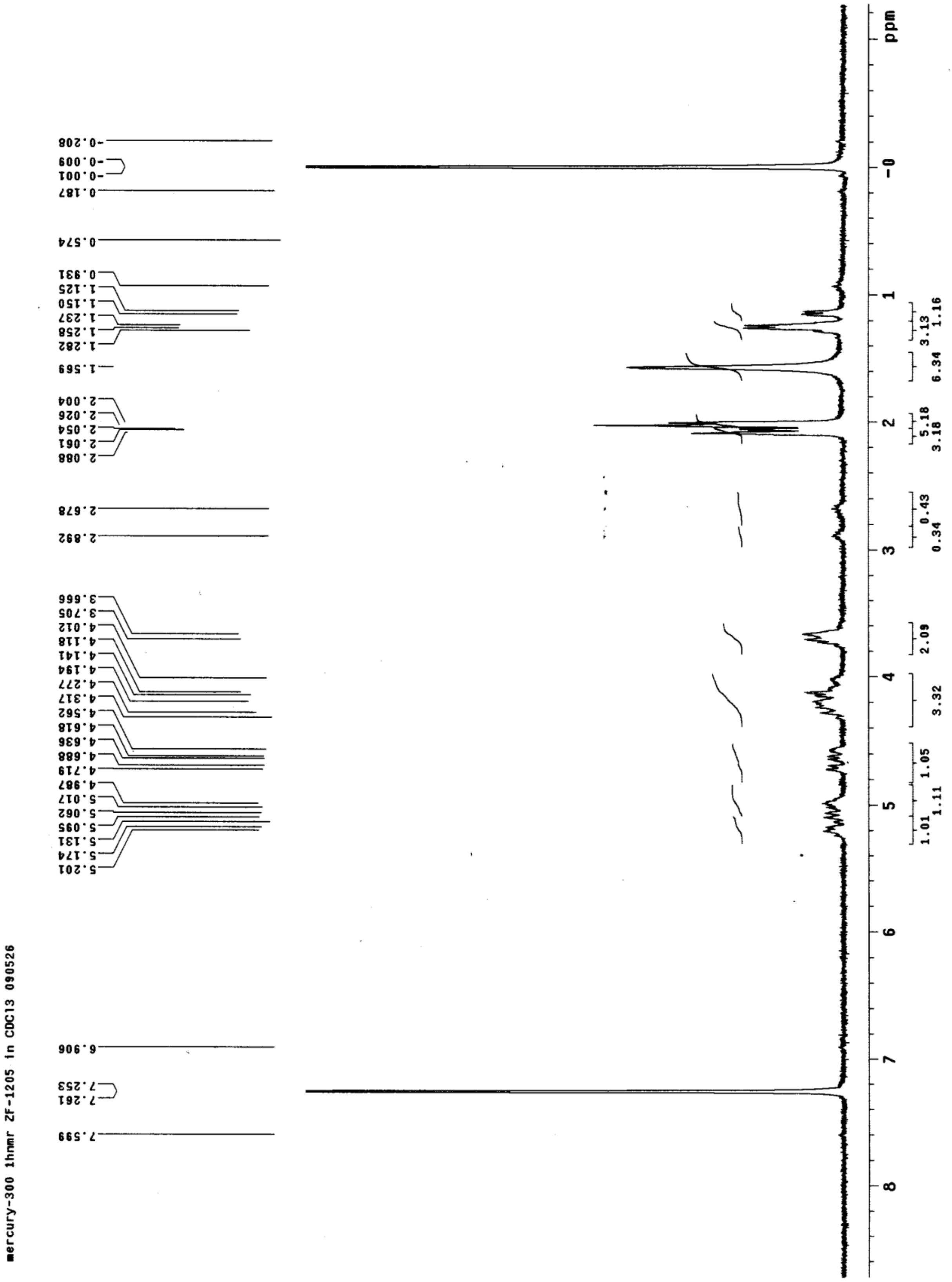

Figure 22. HRMS of compound **4b**.

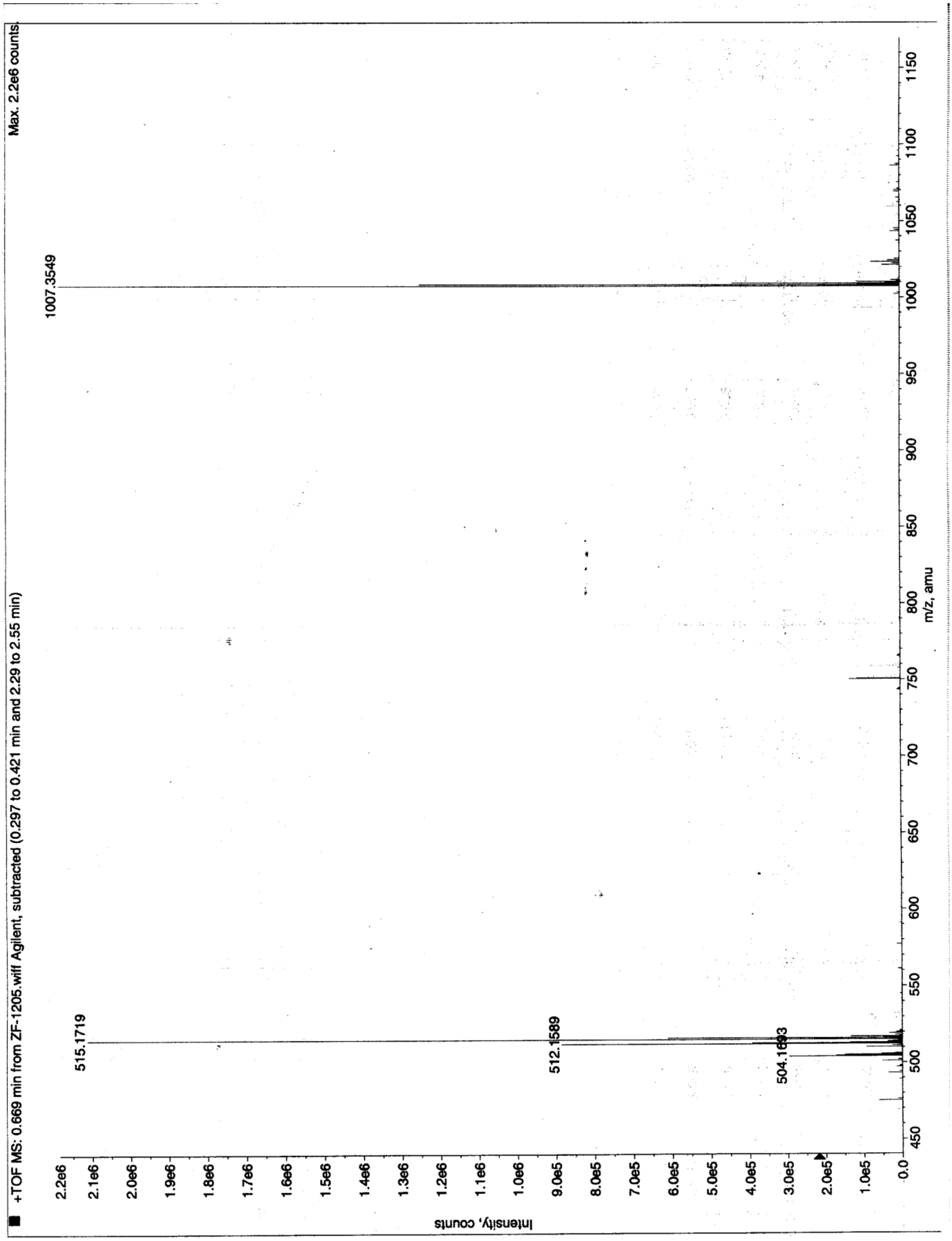

Figure 23. ^{1}H-NMR of compound **4c**.

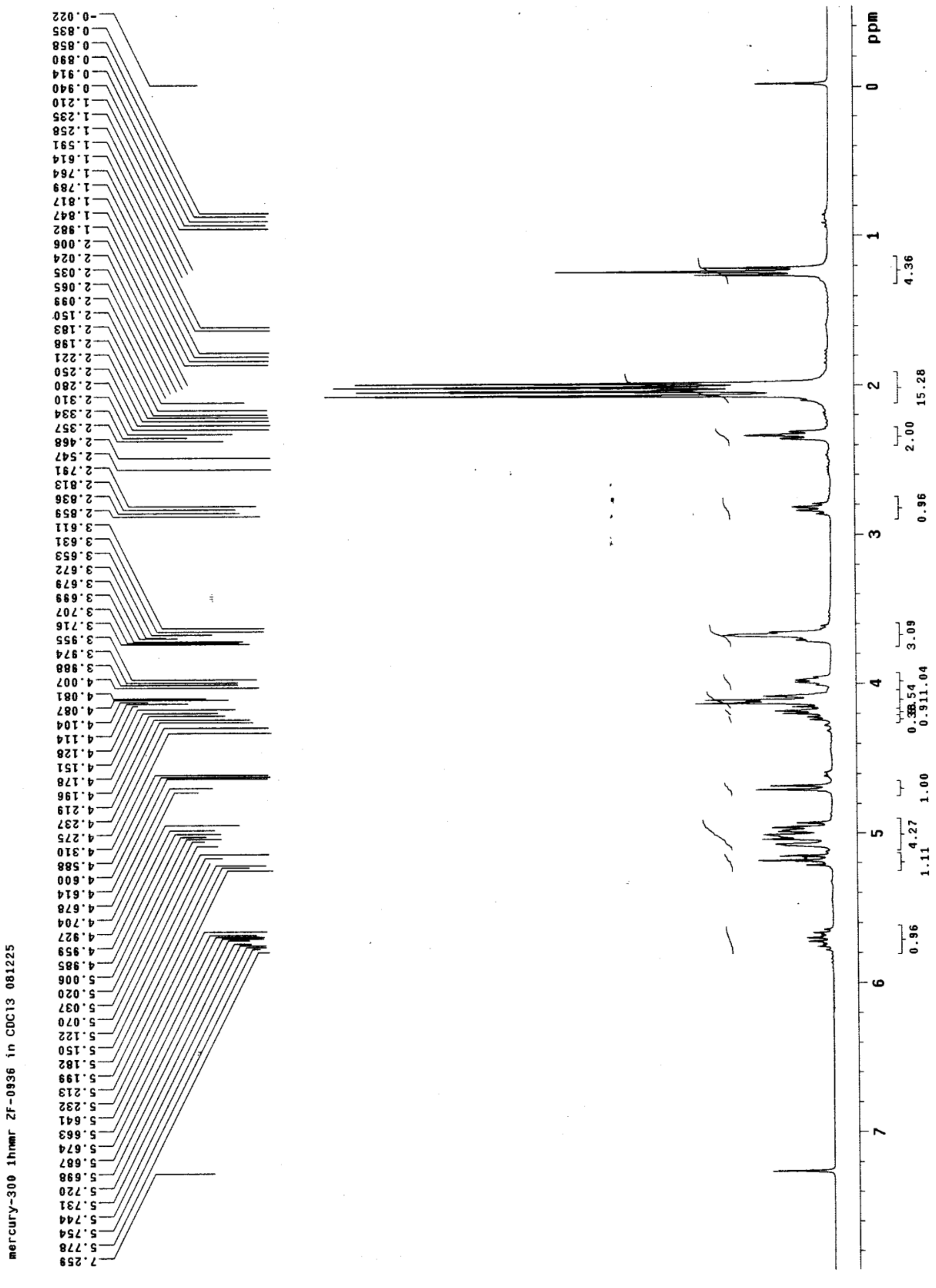

Figure 24. HRMS of compound **4c**.

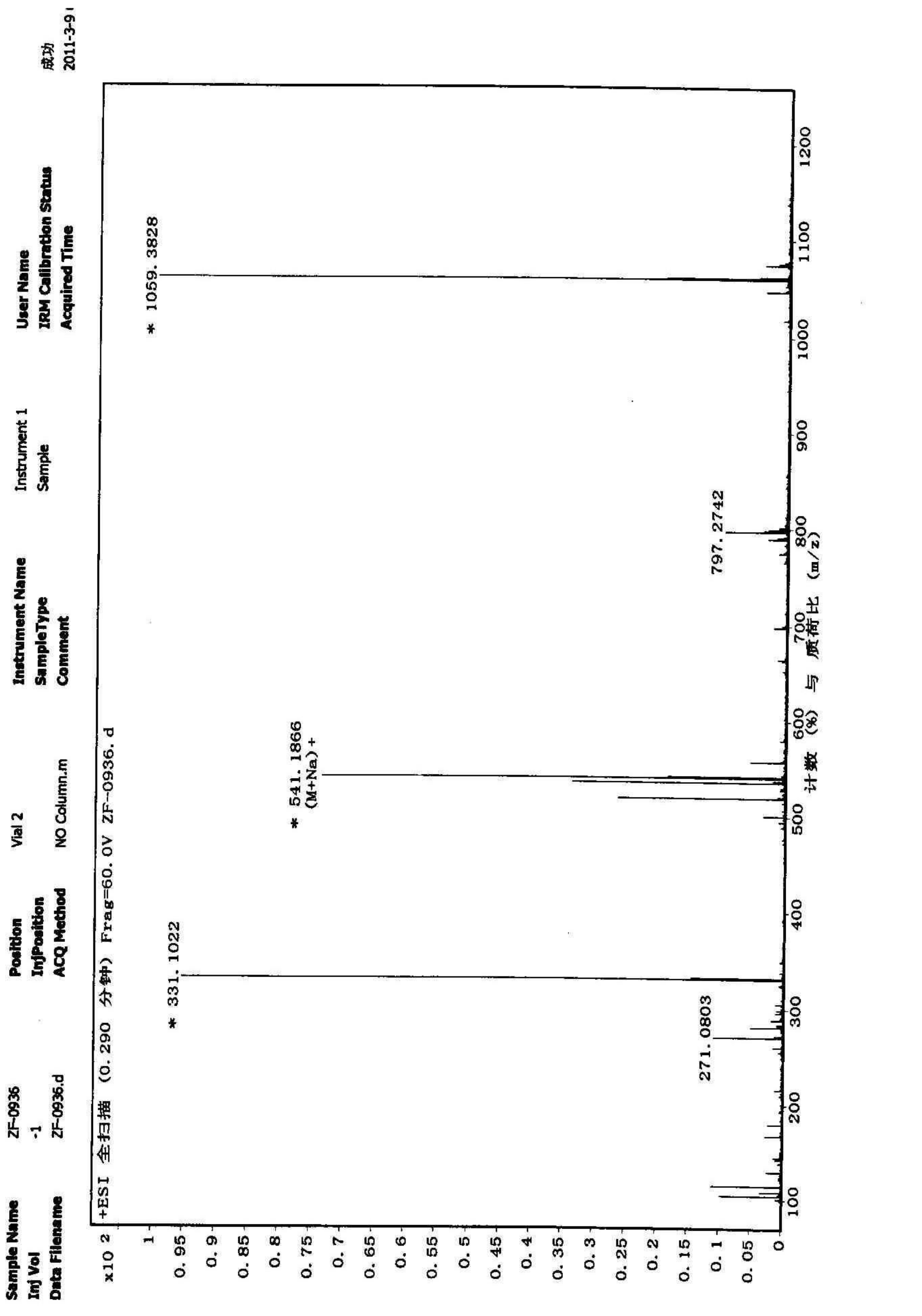

Figure 25. ^{1}H-NMR of compound **4d**.

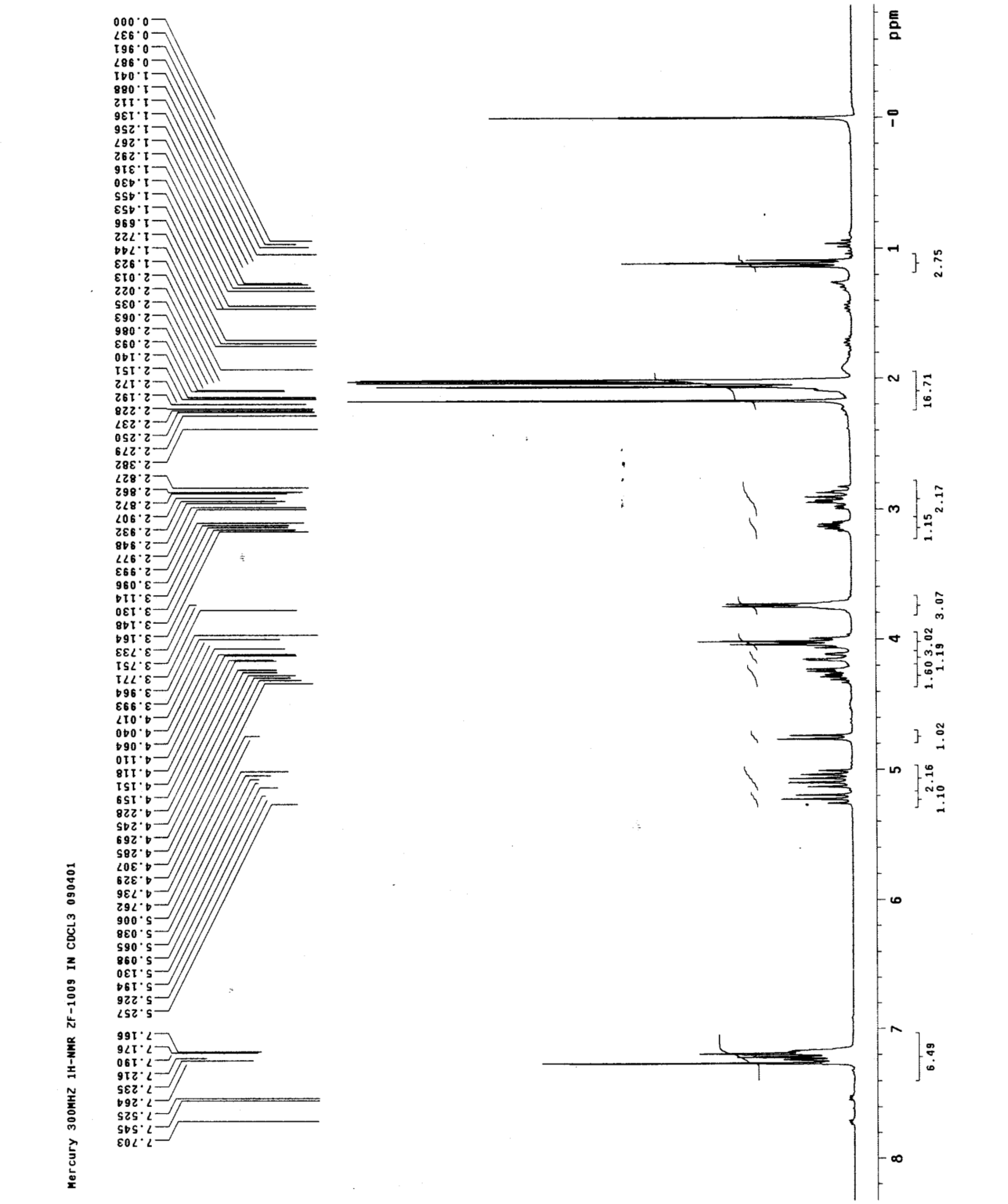

Figure 26. HRMS of compound **4d**.

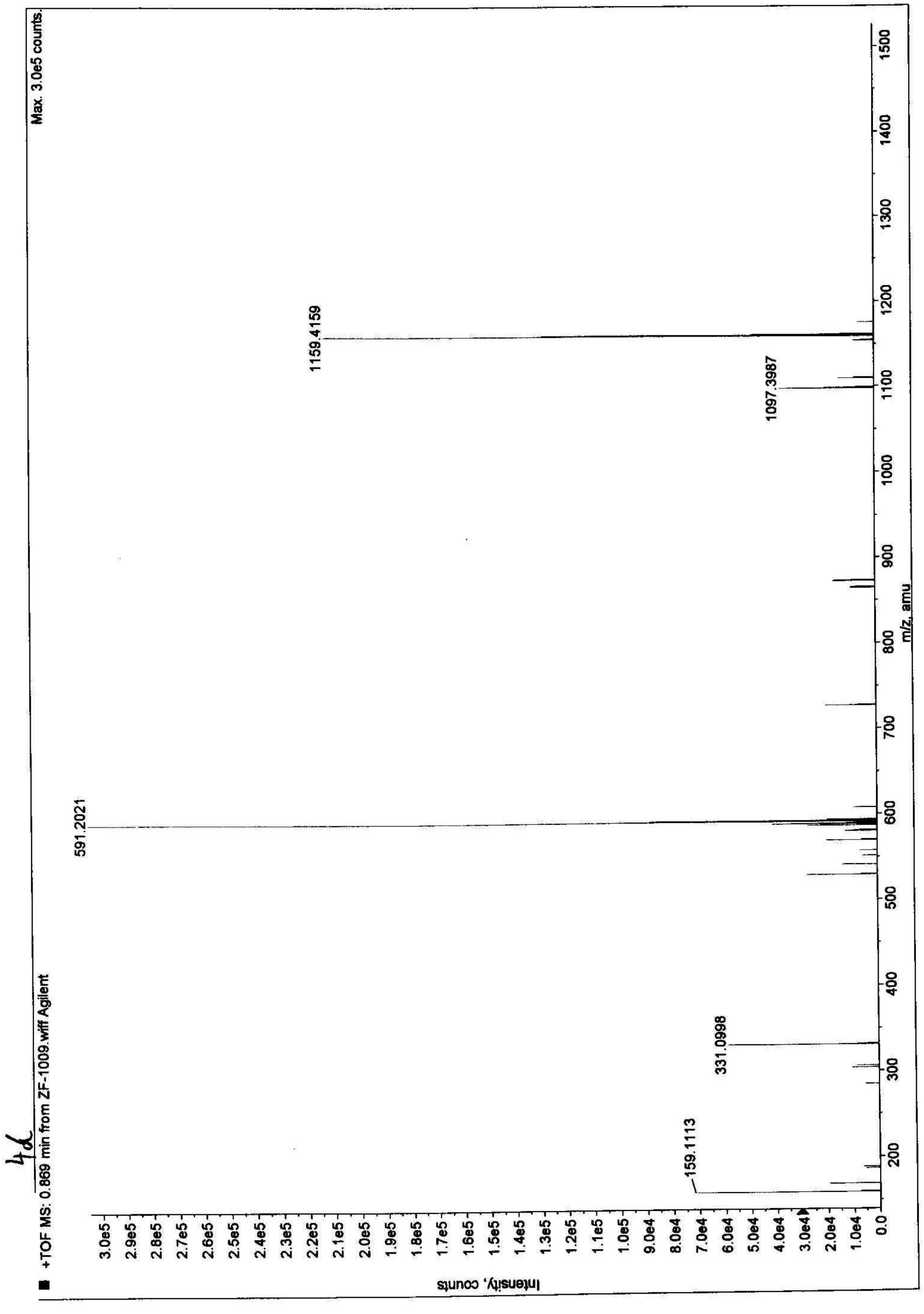

Figure 27. ^{1}H-NMR of compound **4e**.

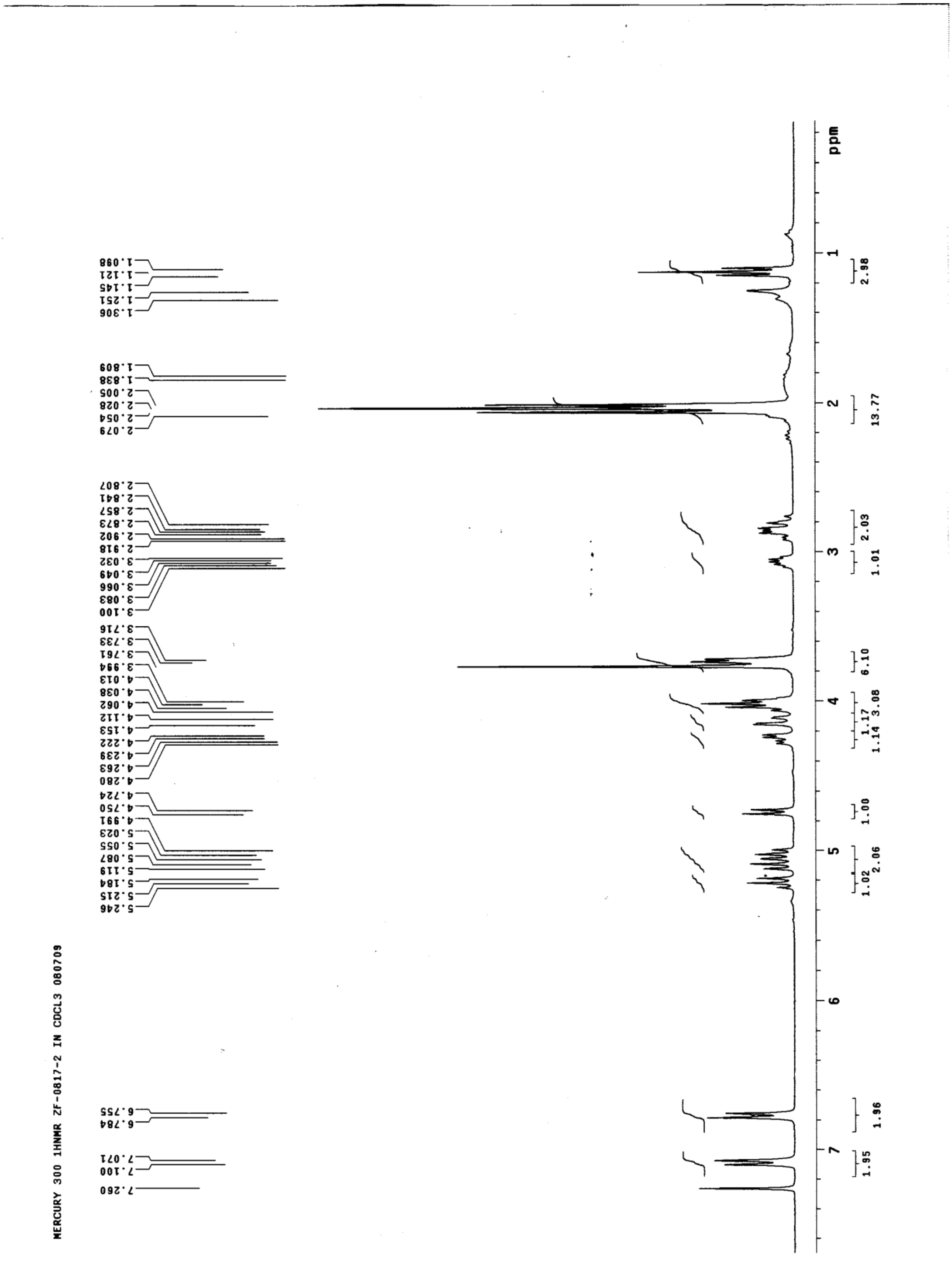

Figure 28. HRMS of compound **4e**.

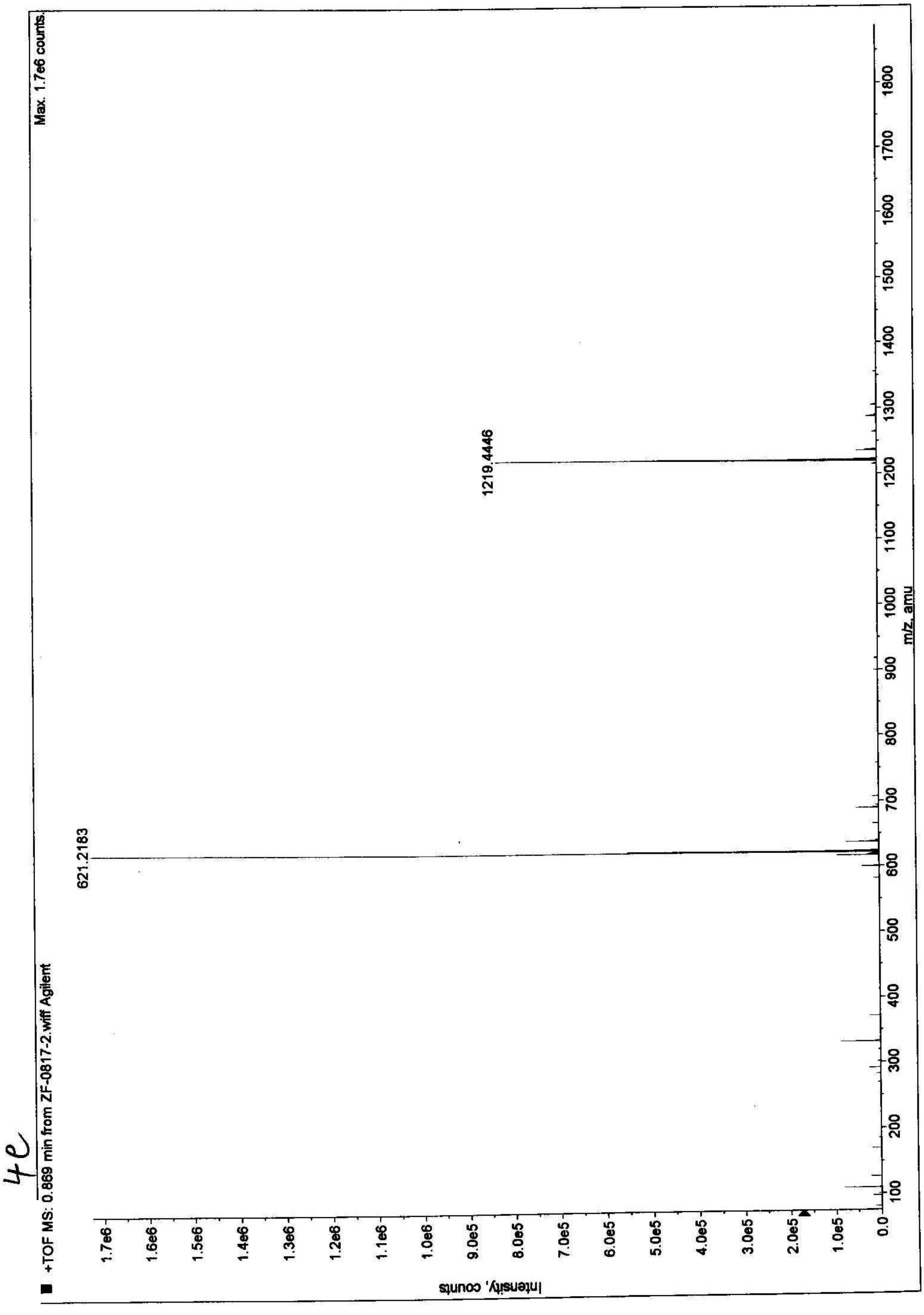

Figure 29. ^{1}H-NMR of compound **5a**.

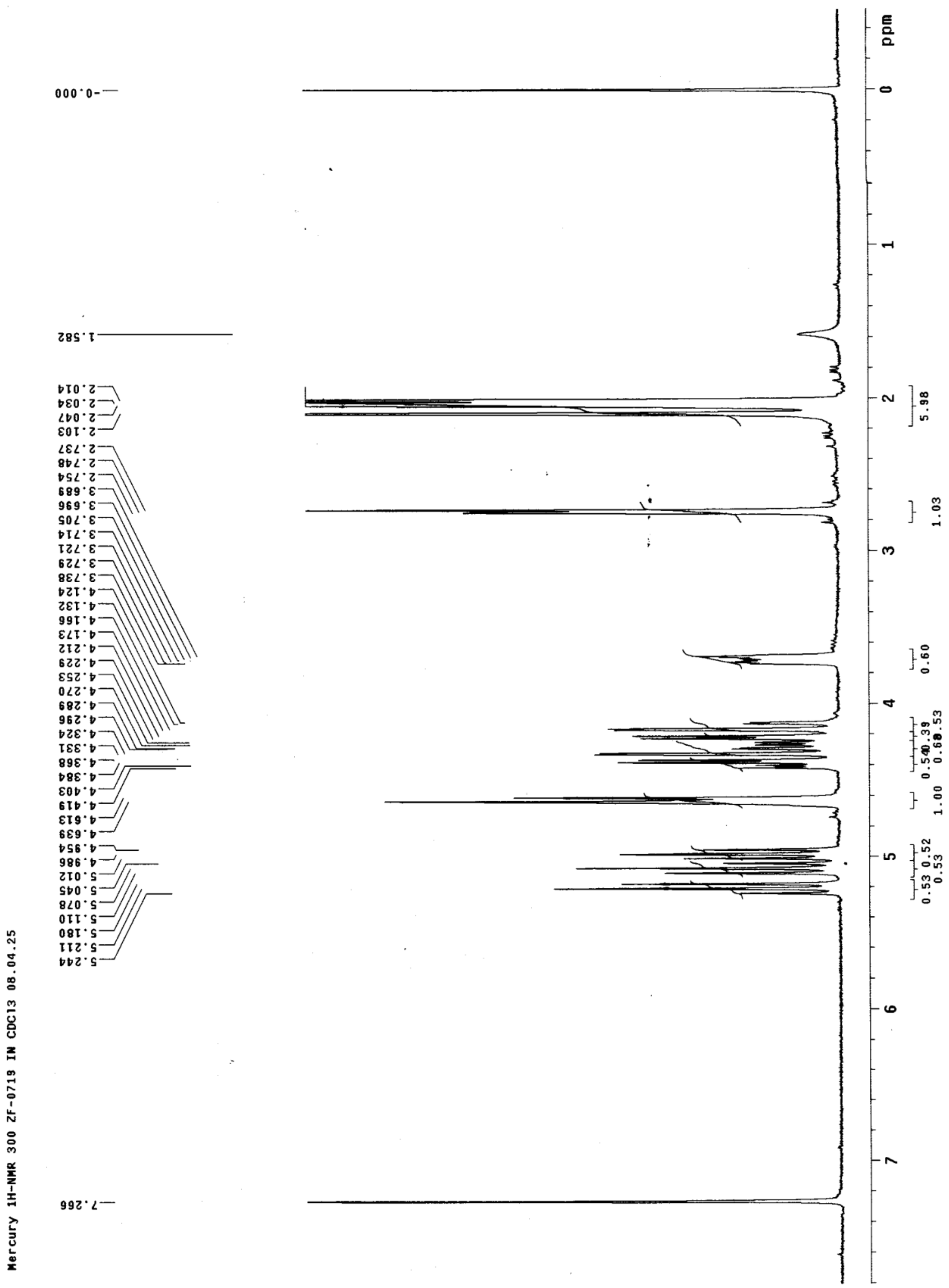

Figure 30. HRMS of compound **5a**.

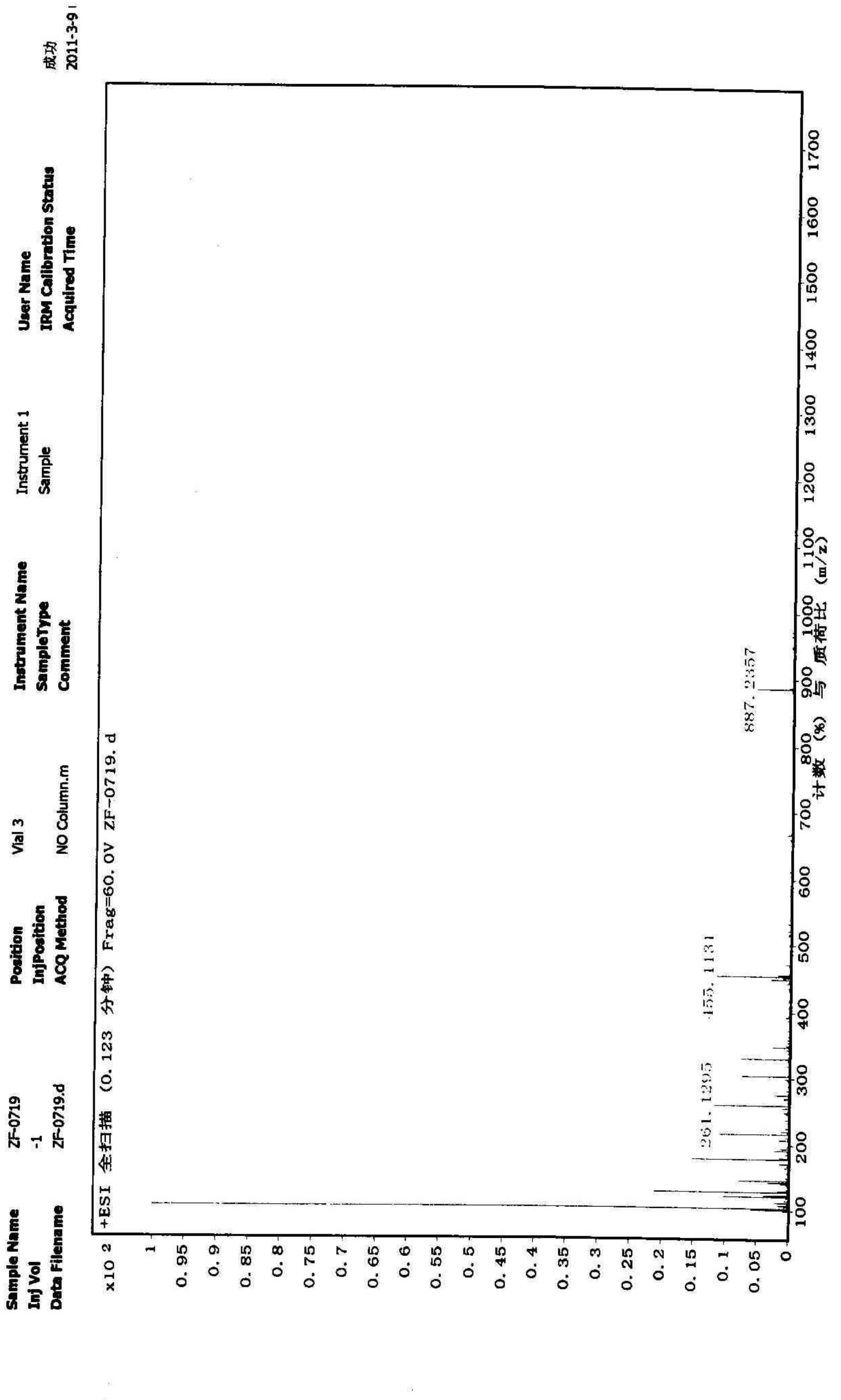

Figure 31. ^{1}H-NMR of compound **5b**.

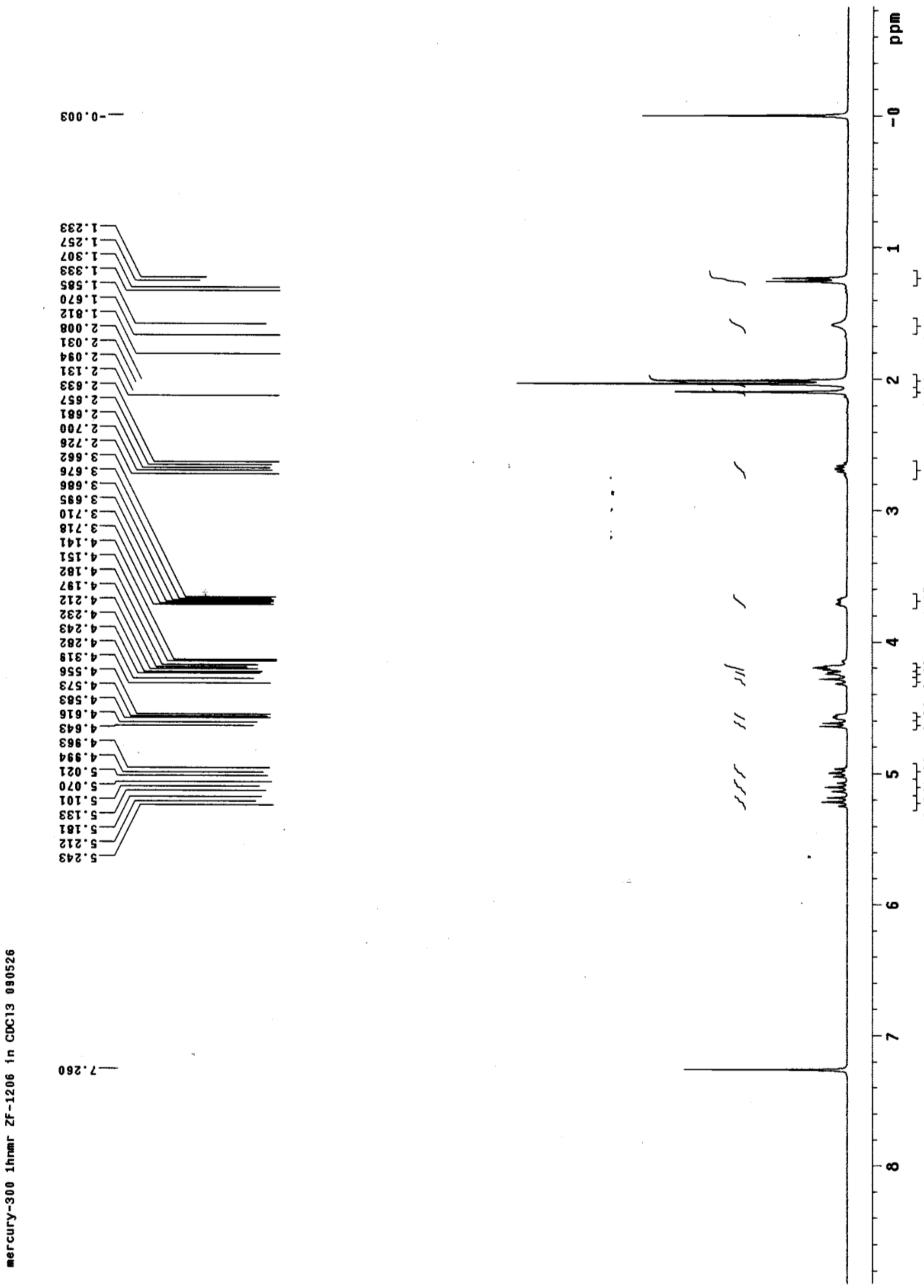

Figure 32. ^{13}C-NMR of compound **5b**.

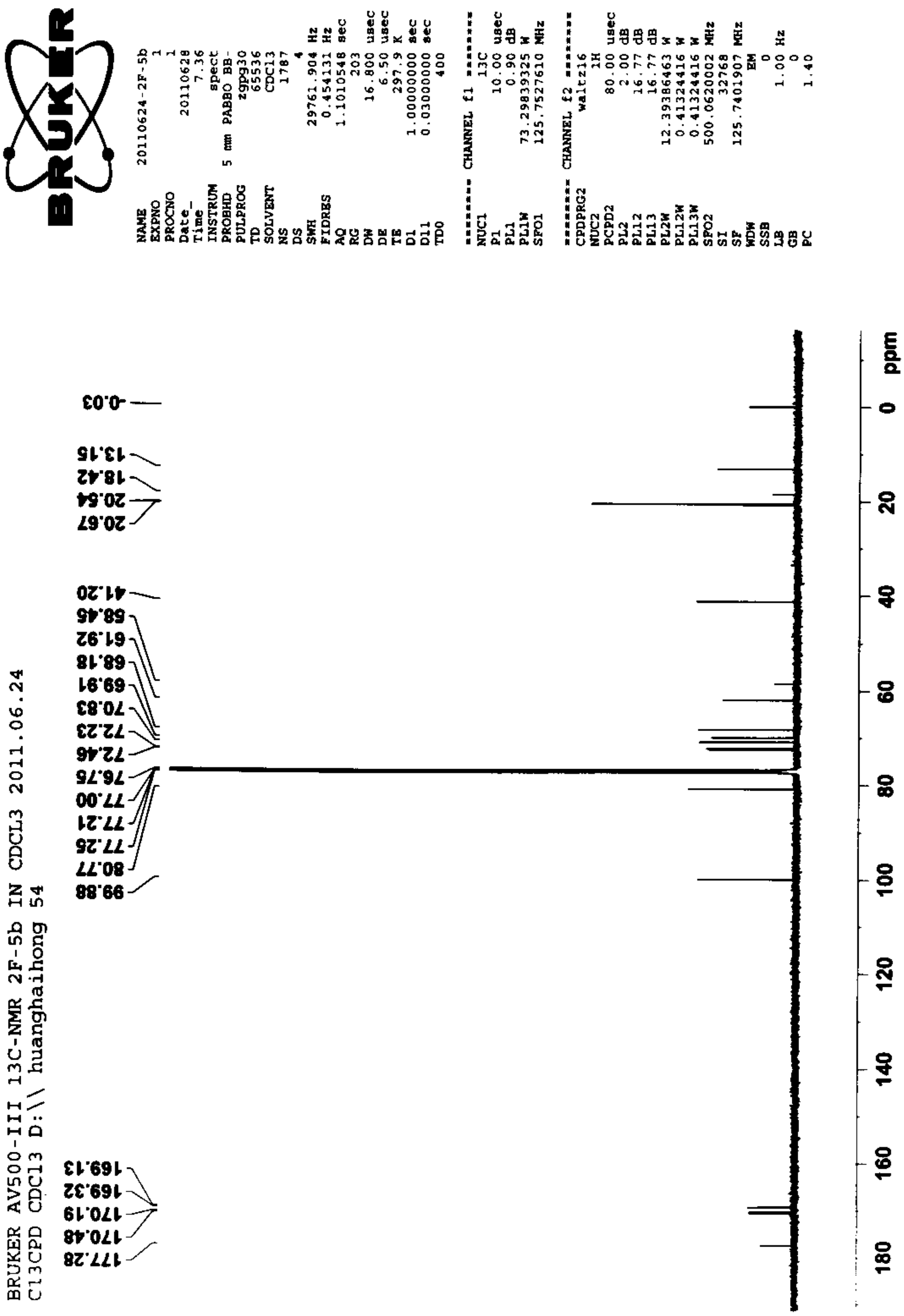

Figure 33. HRMS of compound **5b**.

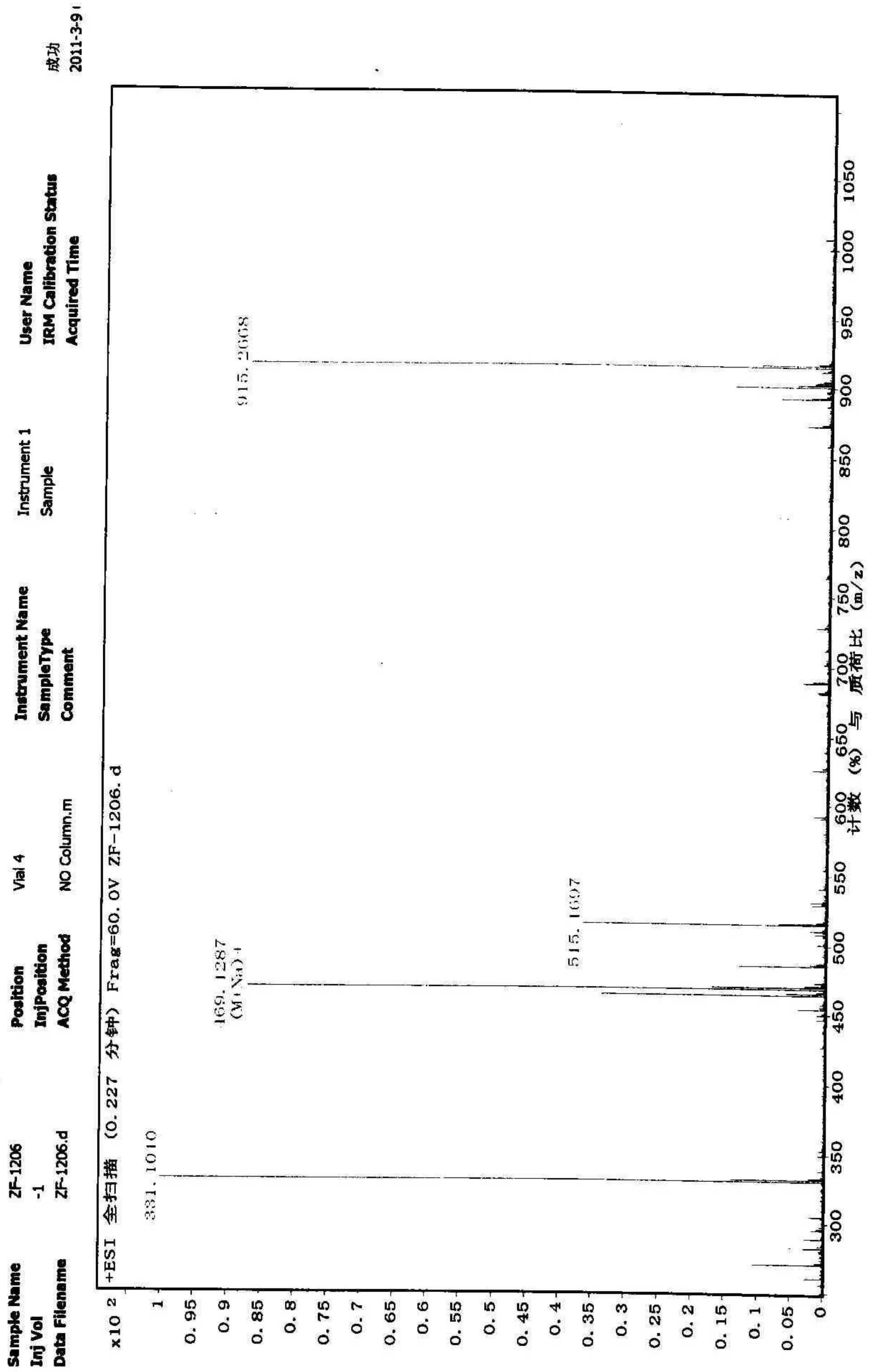

Figure 34. ^{1}H-NMR of compound **5c**.

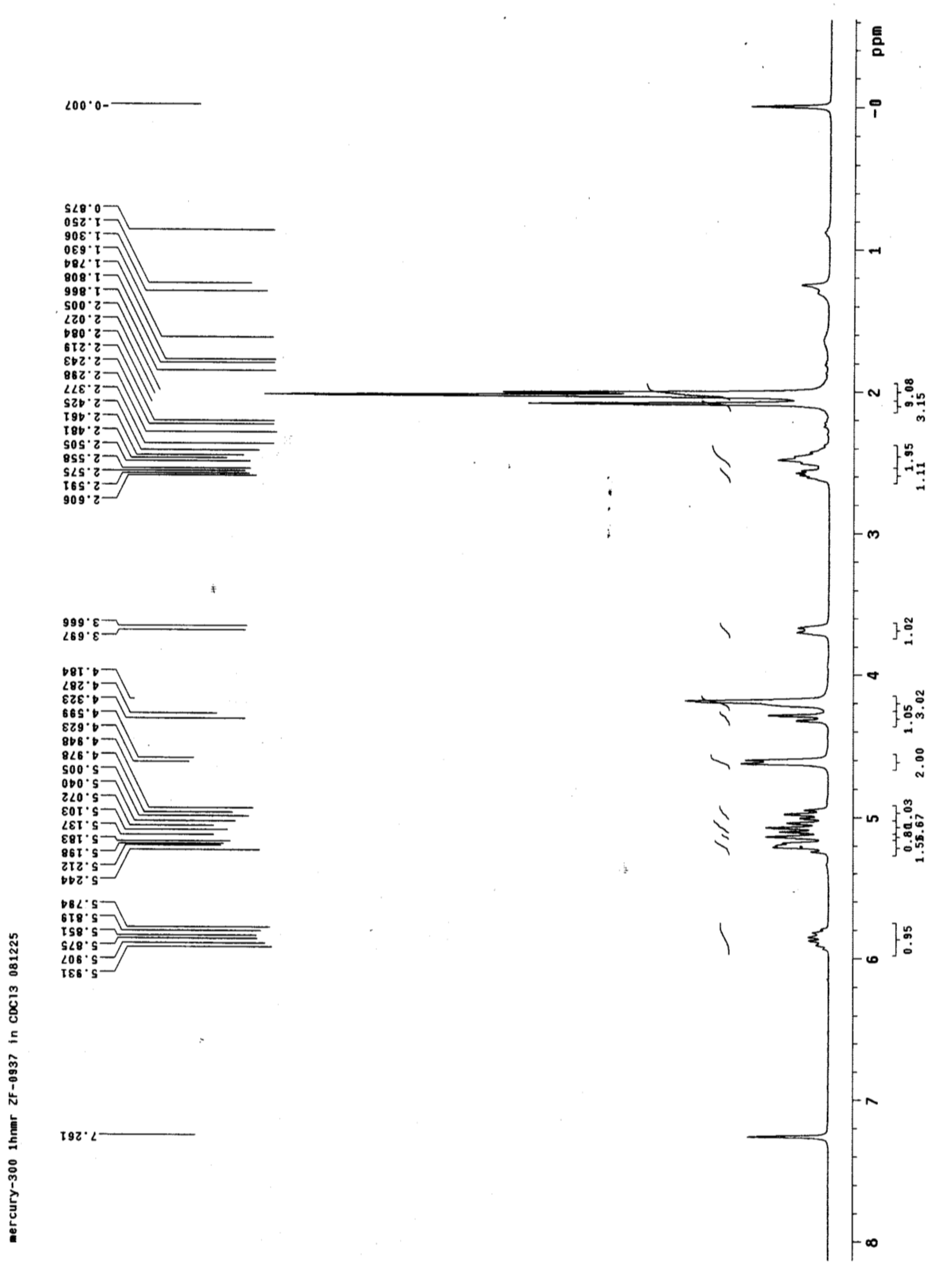

Figure 35. ^{13}C-NMR of compound **5c**.

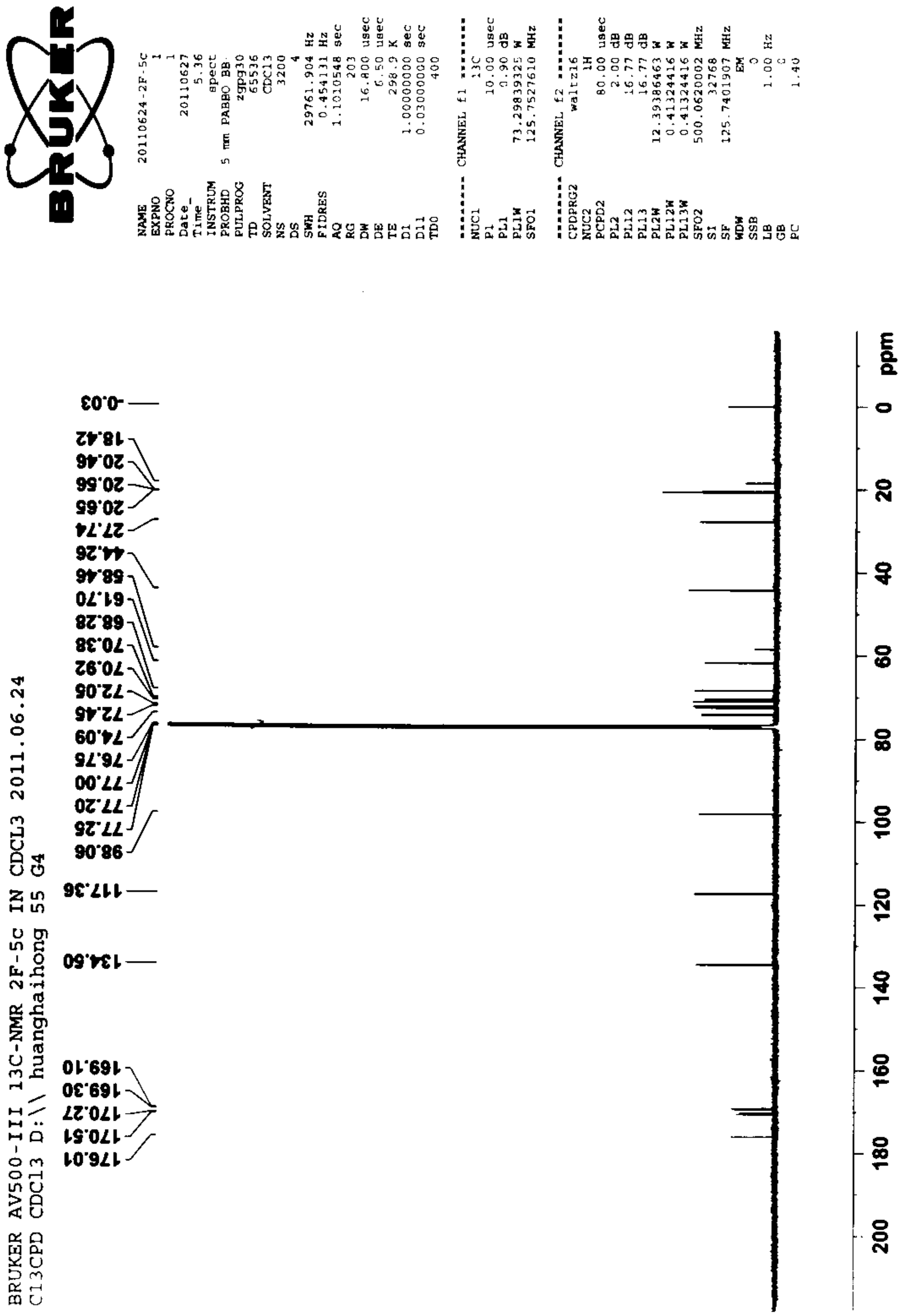

Figure 36. HRMS of compound **5c**.

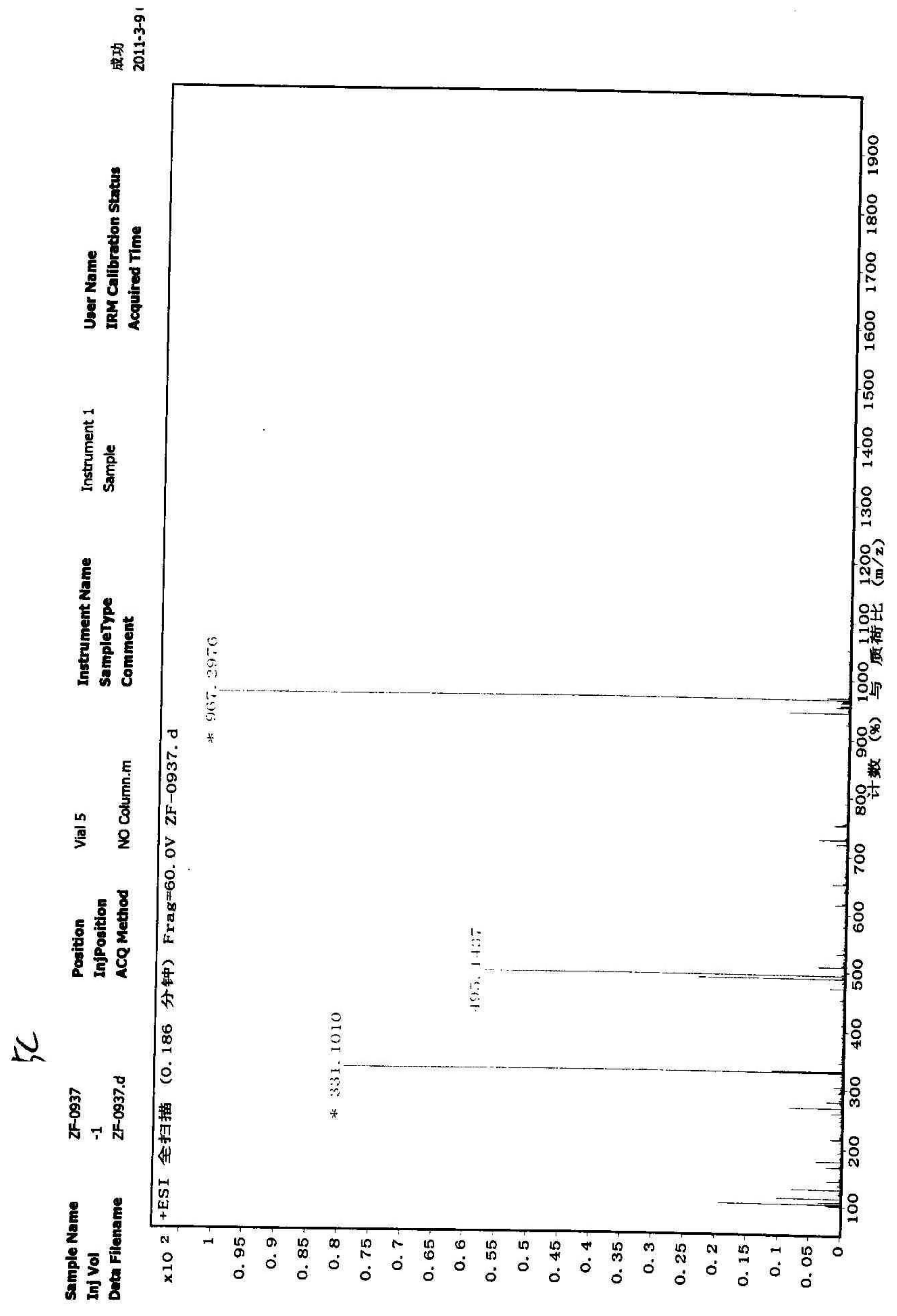

Figure 37. ^{1}H-NMR of compound **5d**.

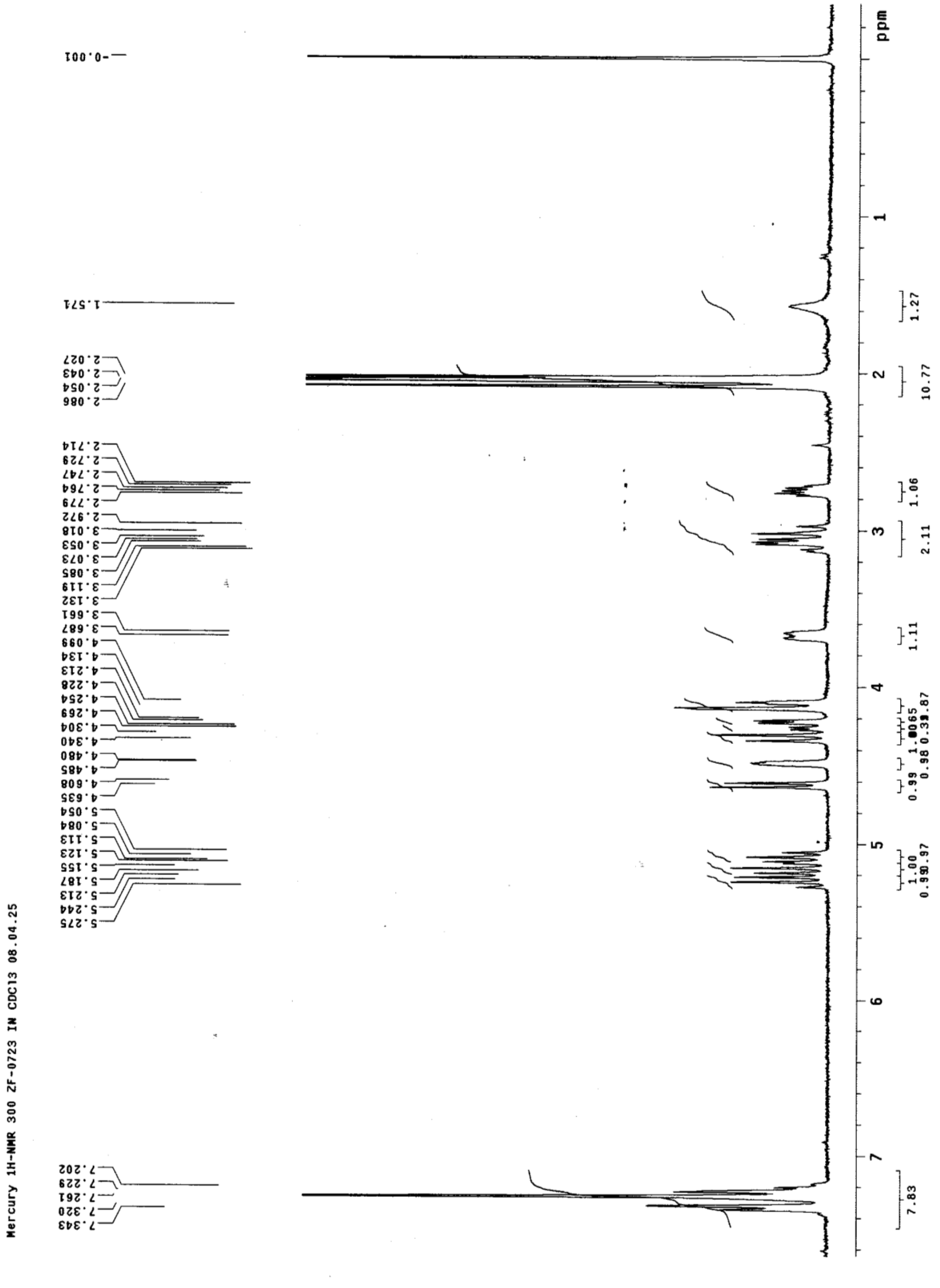

Figure 38. NOE of compound **5d**.

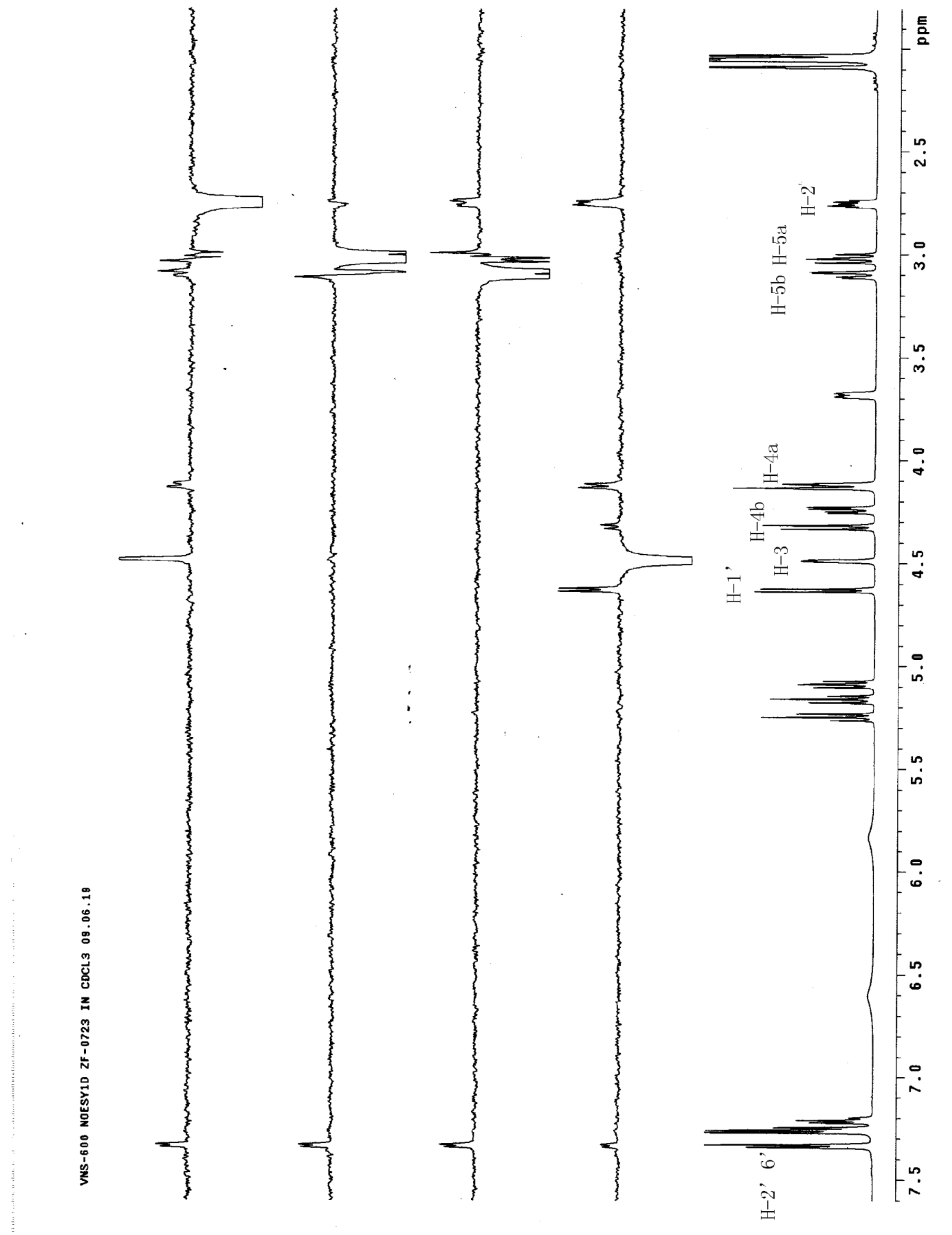

Figure 39. ^{13}C-NMR of compound **5d**.

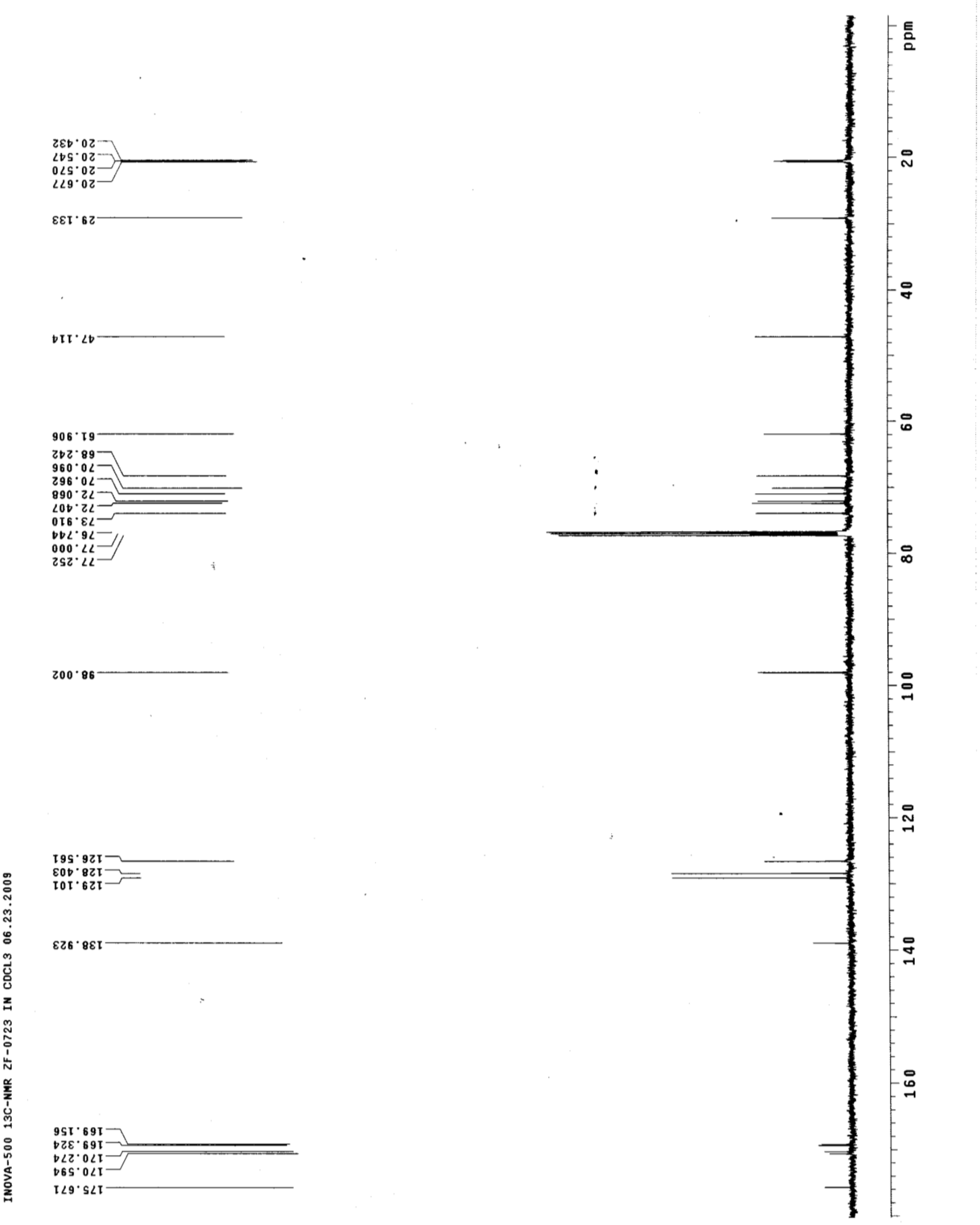

Figure 40. HRMS of compound **5d**.

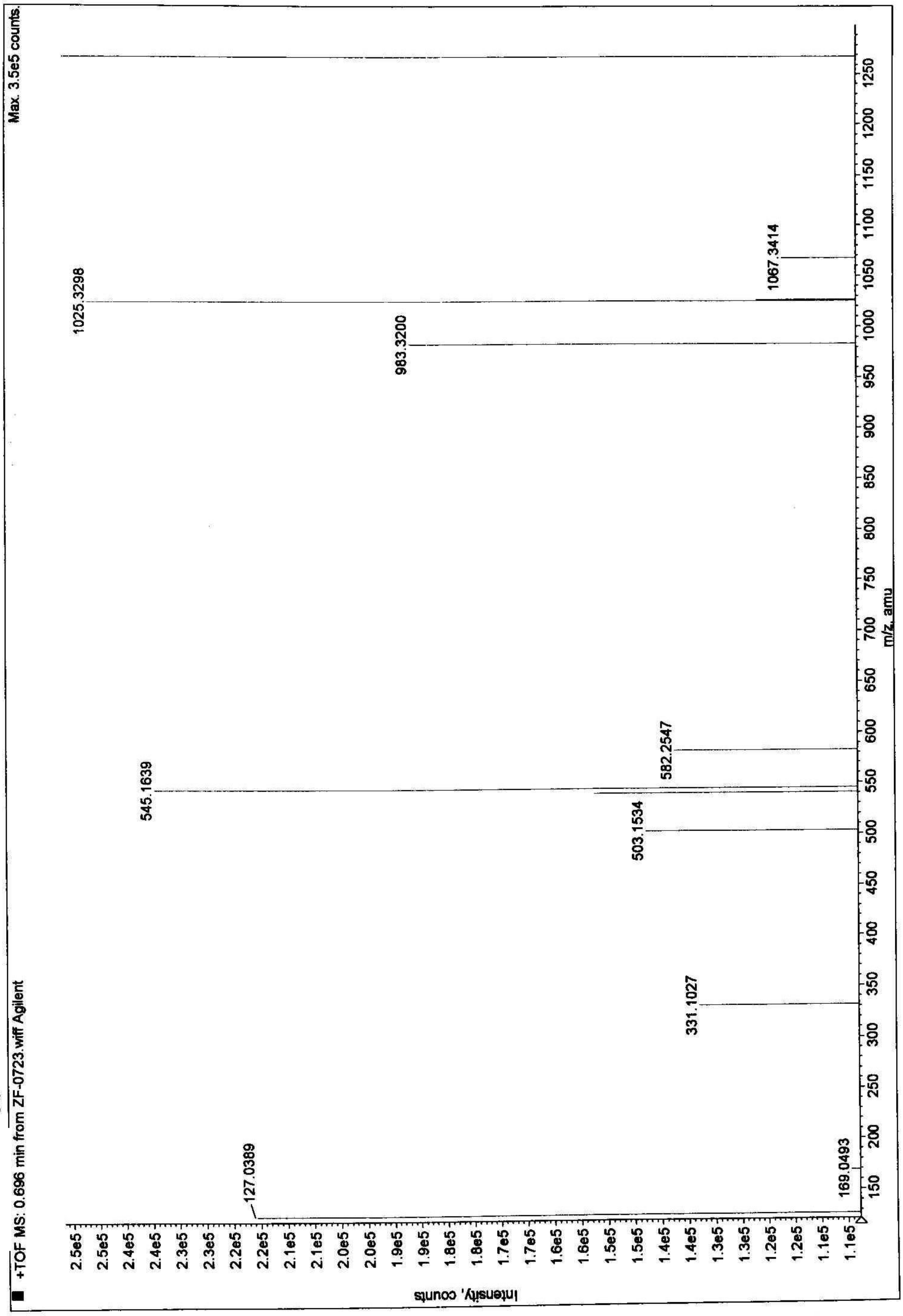

Figure 41. ^{1}H-NMR of compound **5e**.

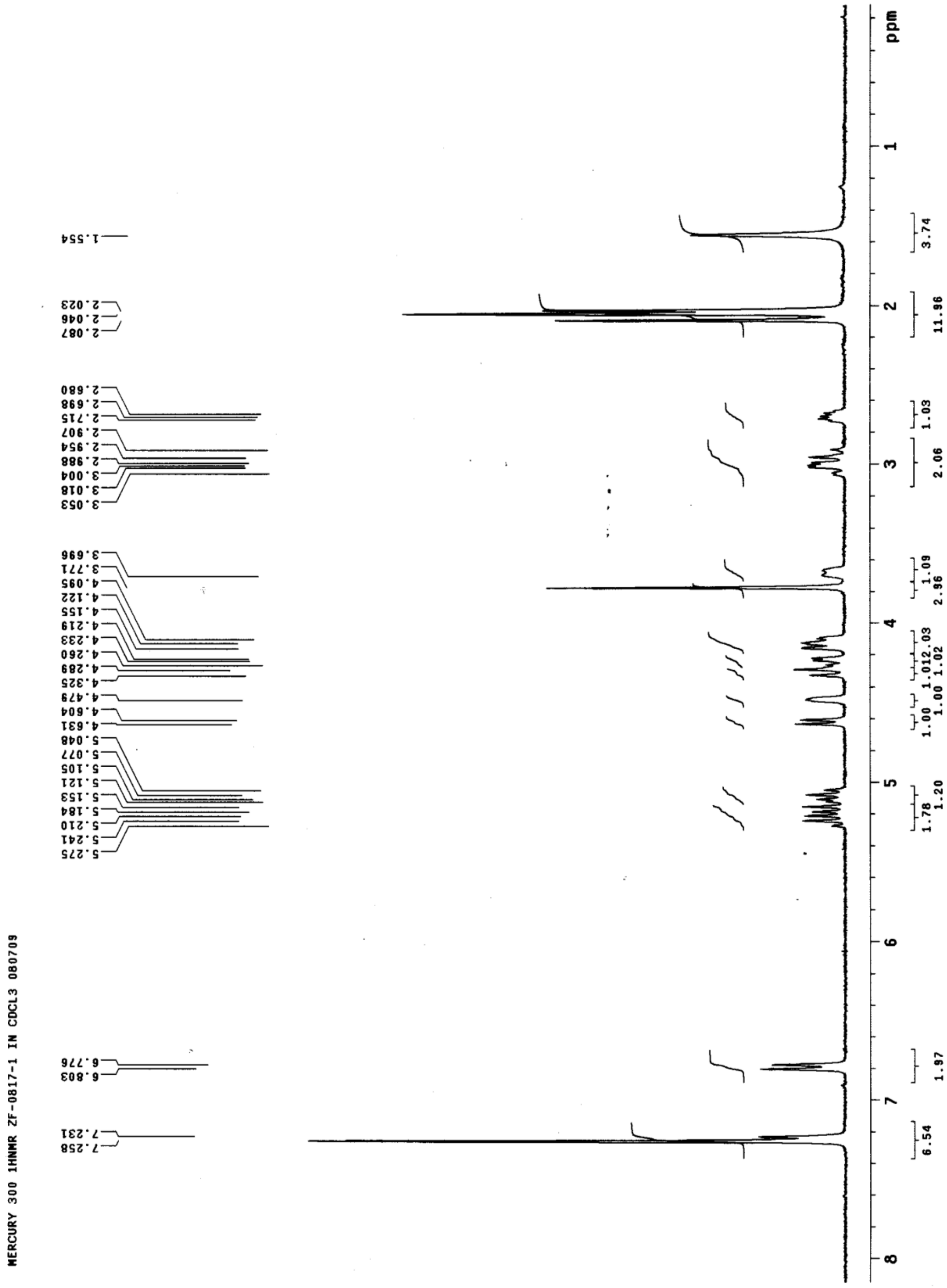

Figure 42. ^{13}C-NMR of compound **5e**.

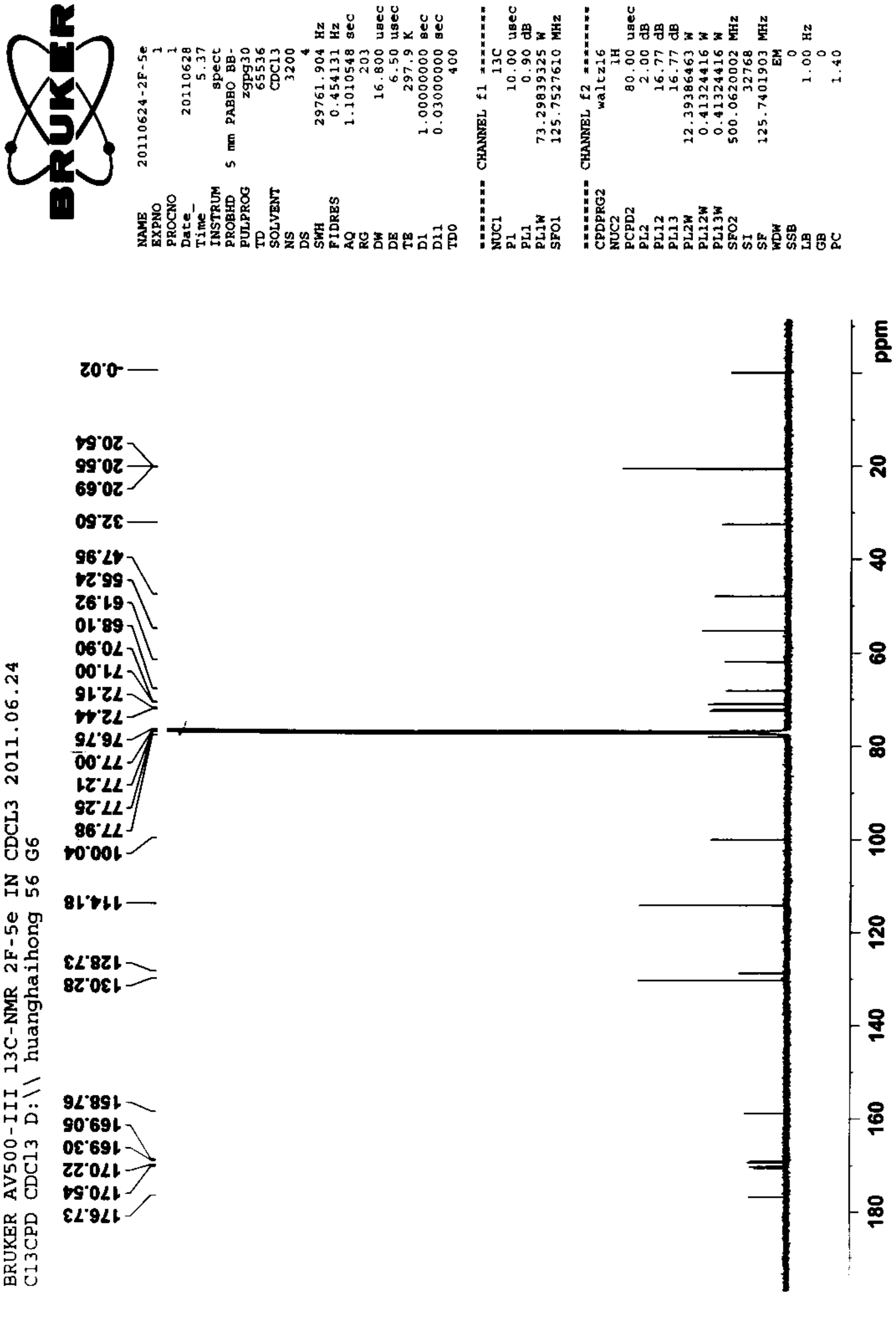

Figure 43. HRMS of compound **5e**.

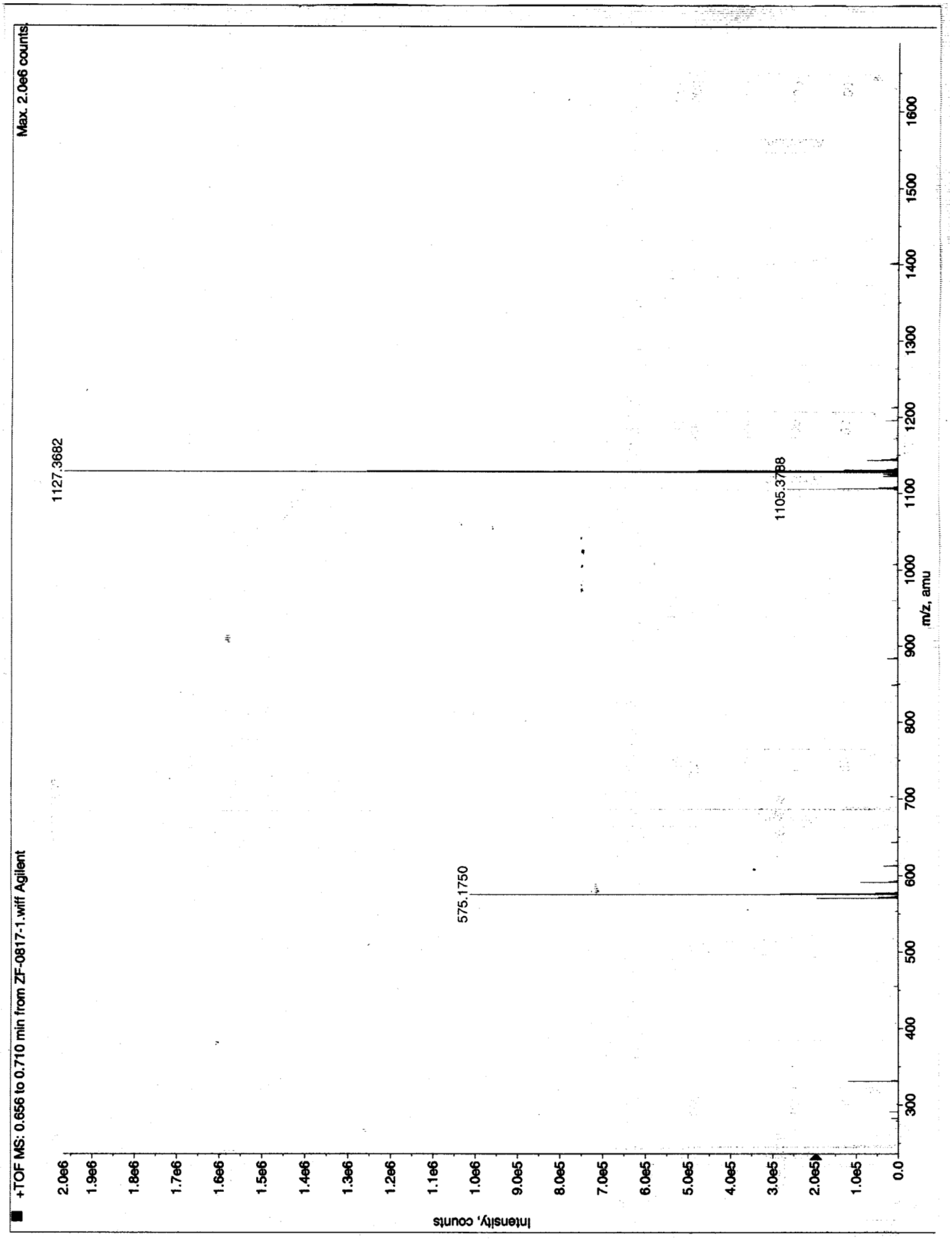

Figure 44. ^{1}H-NMR of compound **6a**.

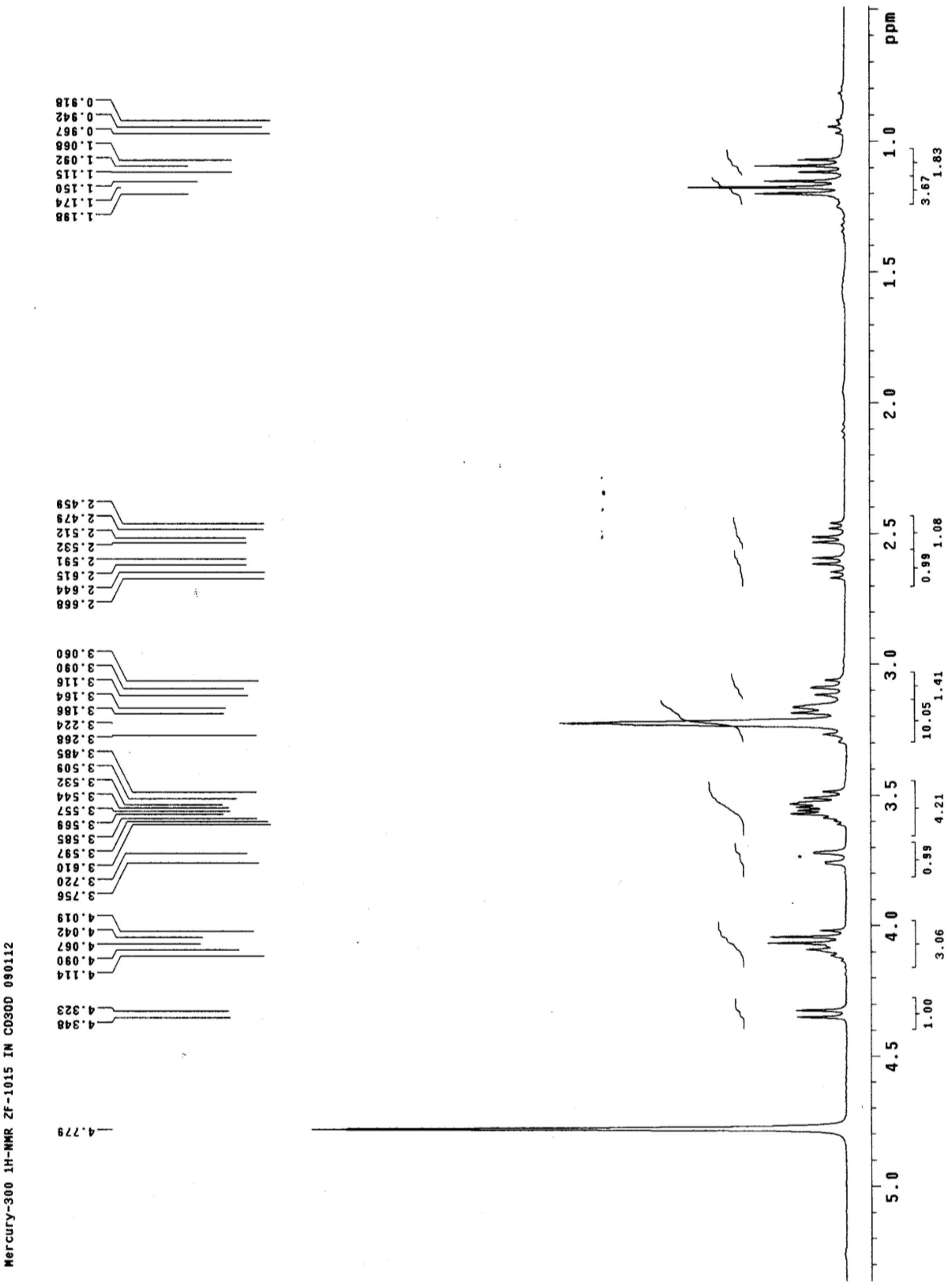

Figure 45. HRMS of compound **6a**.

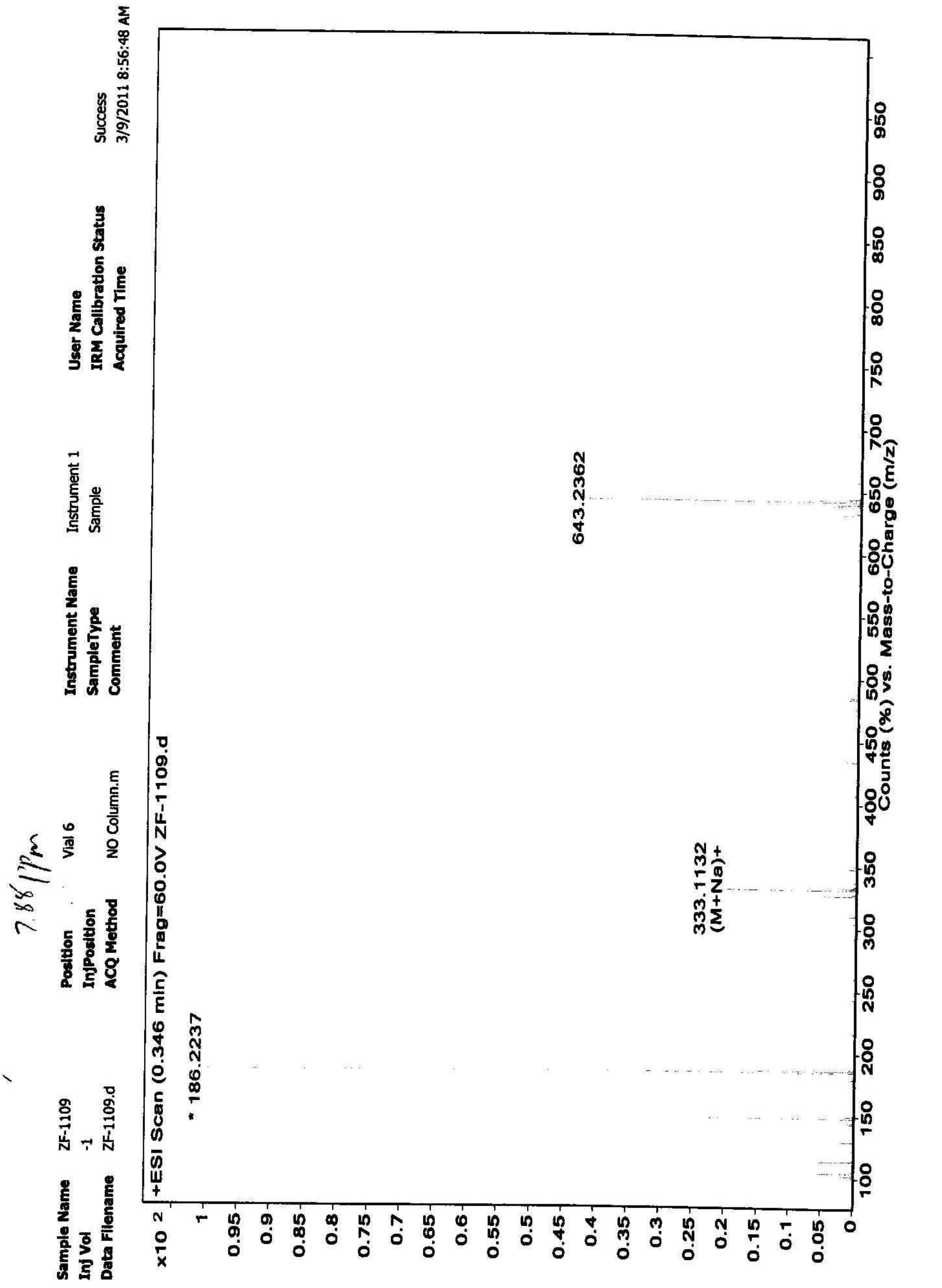

Figure 46. ^{1}H-NMR of compound **6b**.

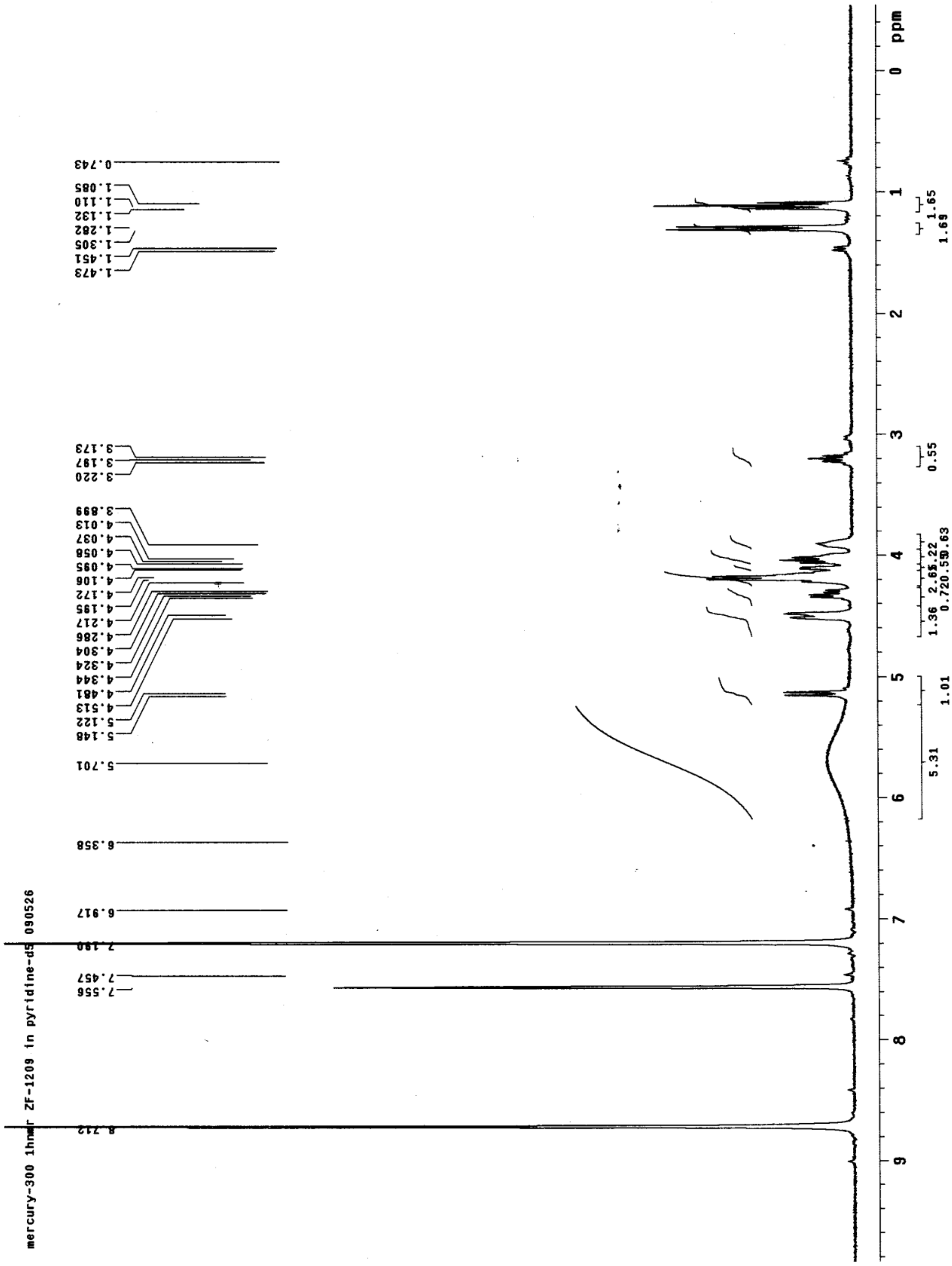

Figure 47. HRMS of compound **6b**.

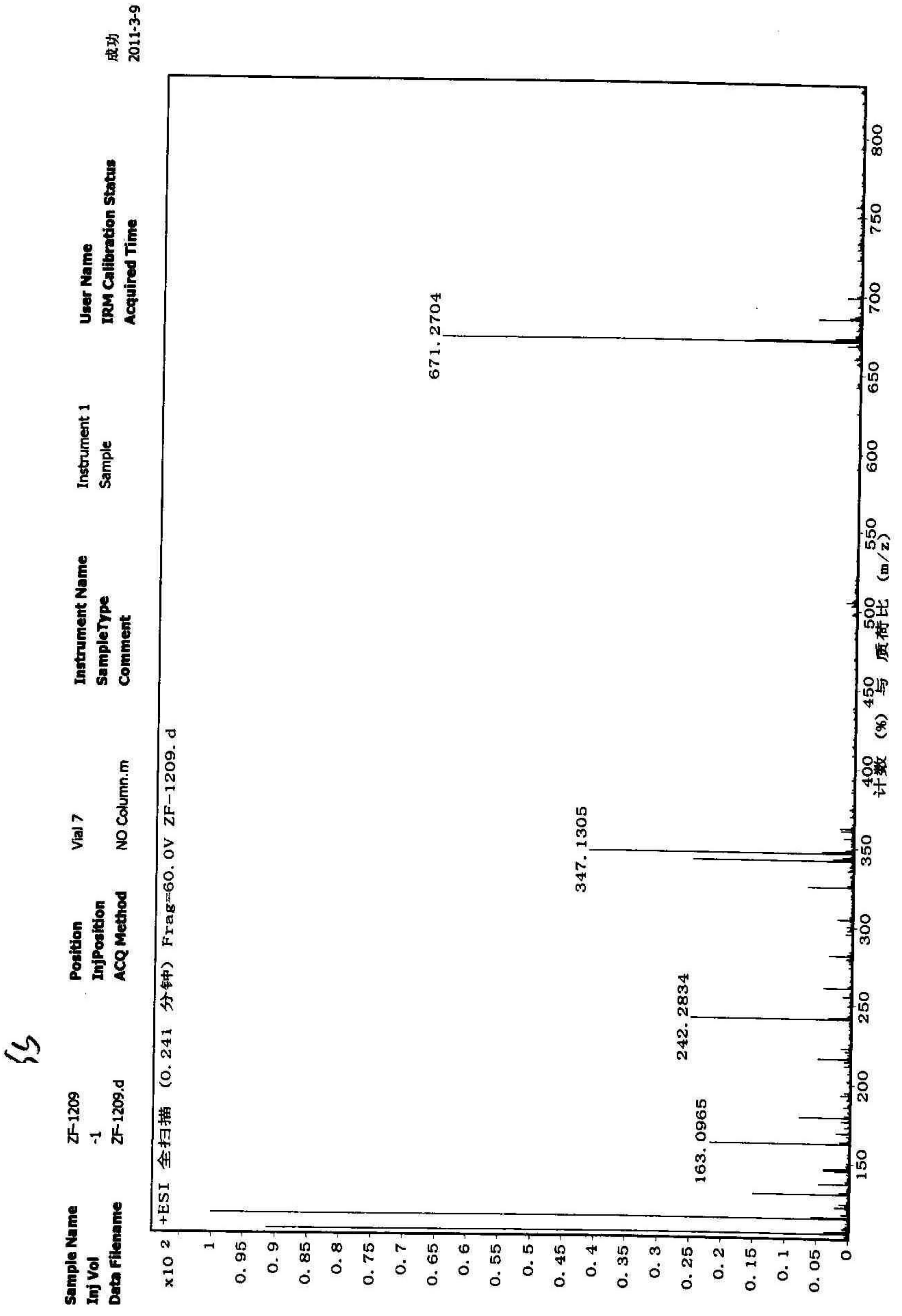

Figure 48. ^{1}H-NMR of compound **6c**.

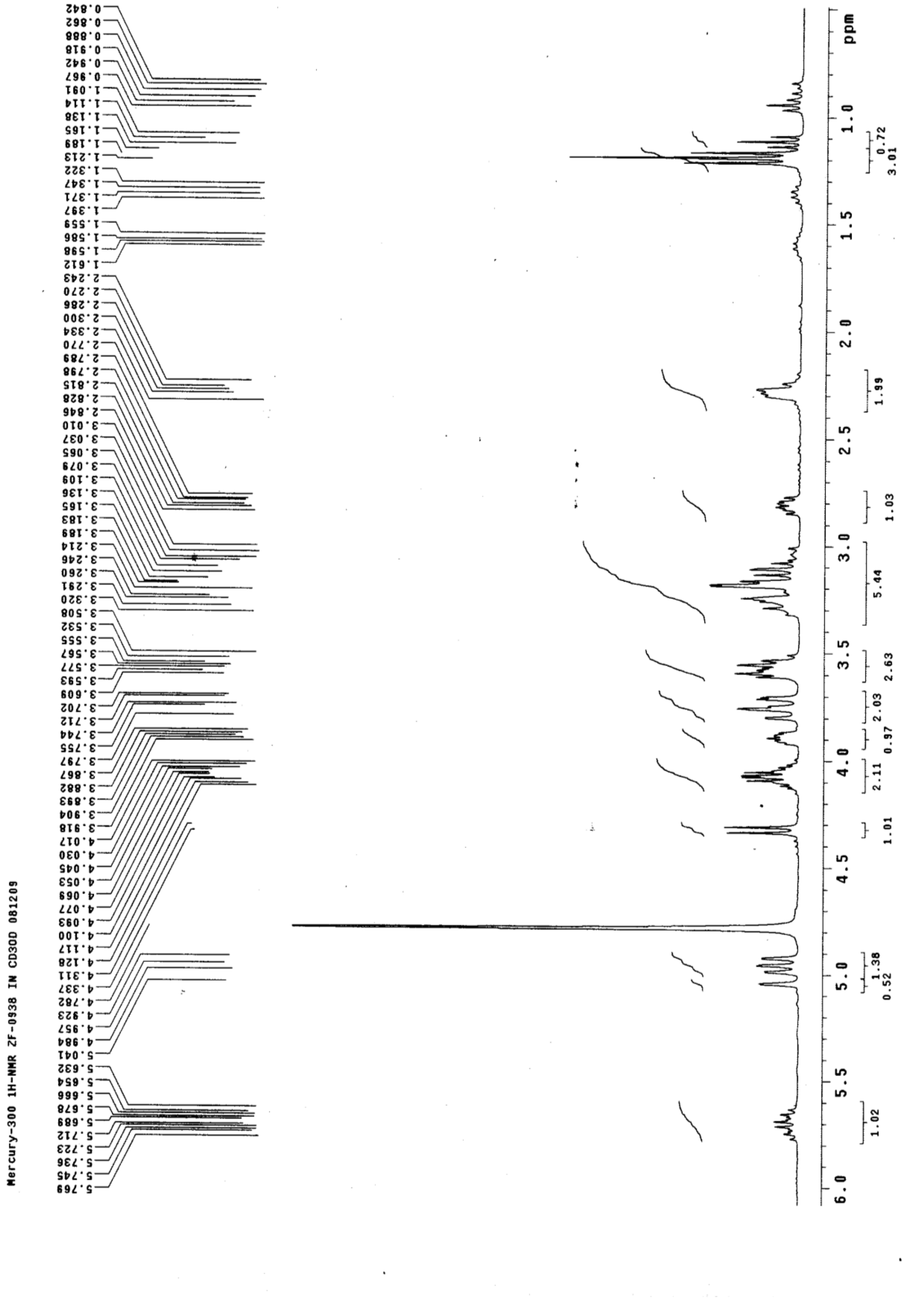

Figure 49. HRMS of compound **6c**.

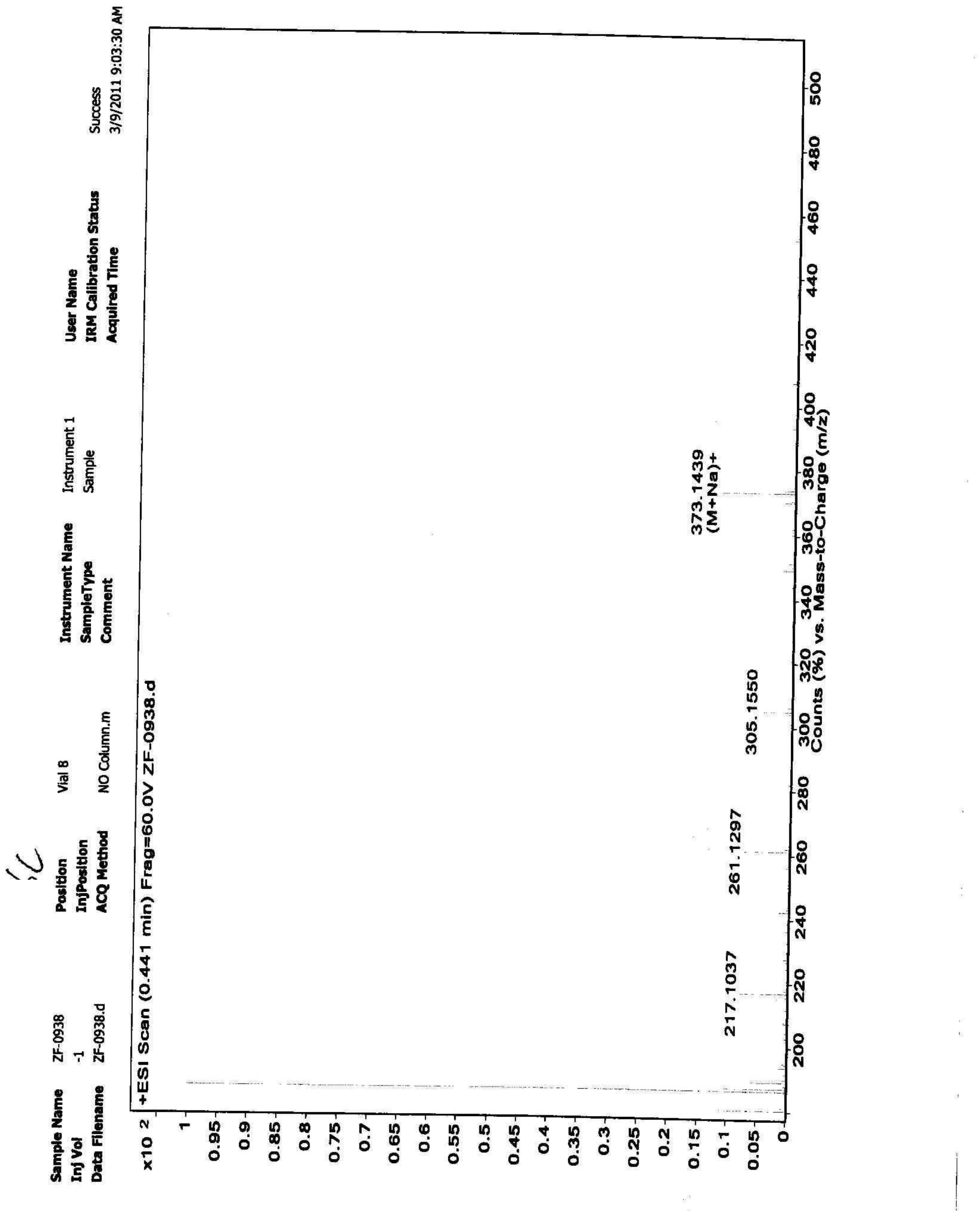

Figure 50. ^{1}H-NMR of compound **6d**.

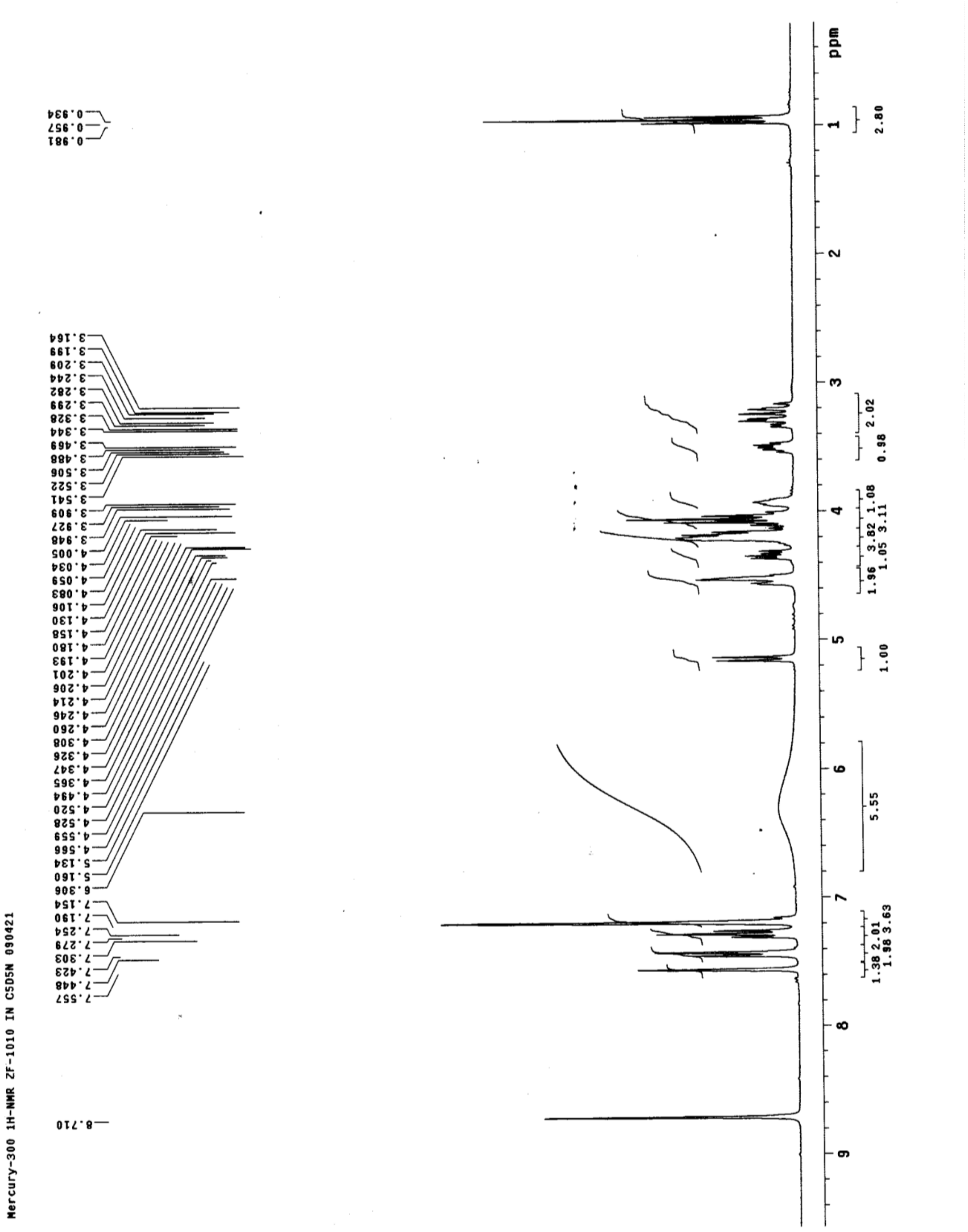

Figure 51. HRMS of compound **6d**.

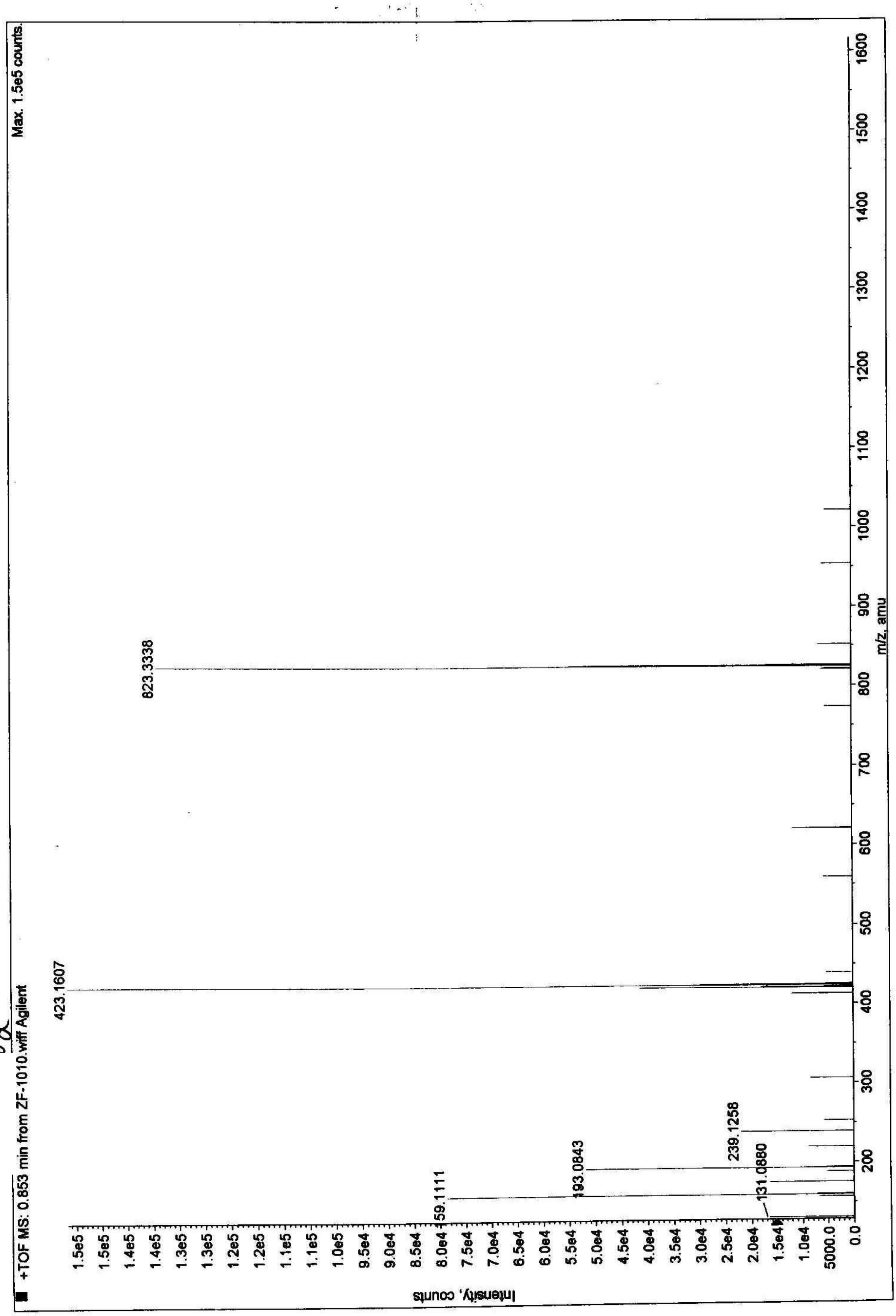

Figure 52. ^{1}H-NMR of compound **6e**.

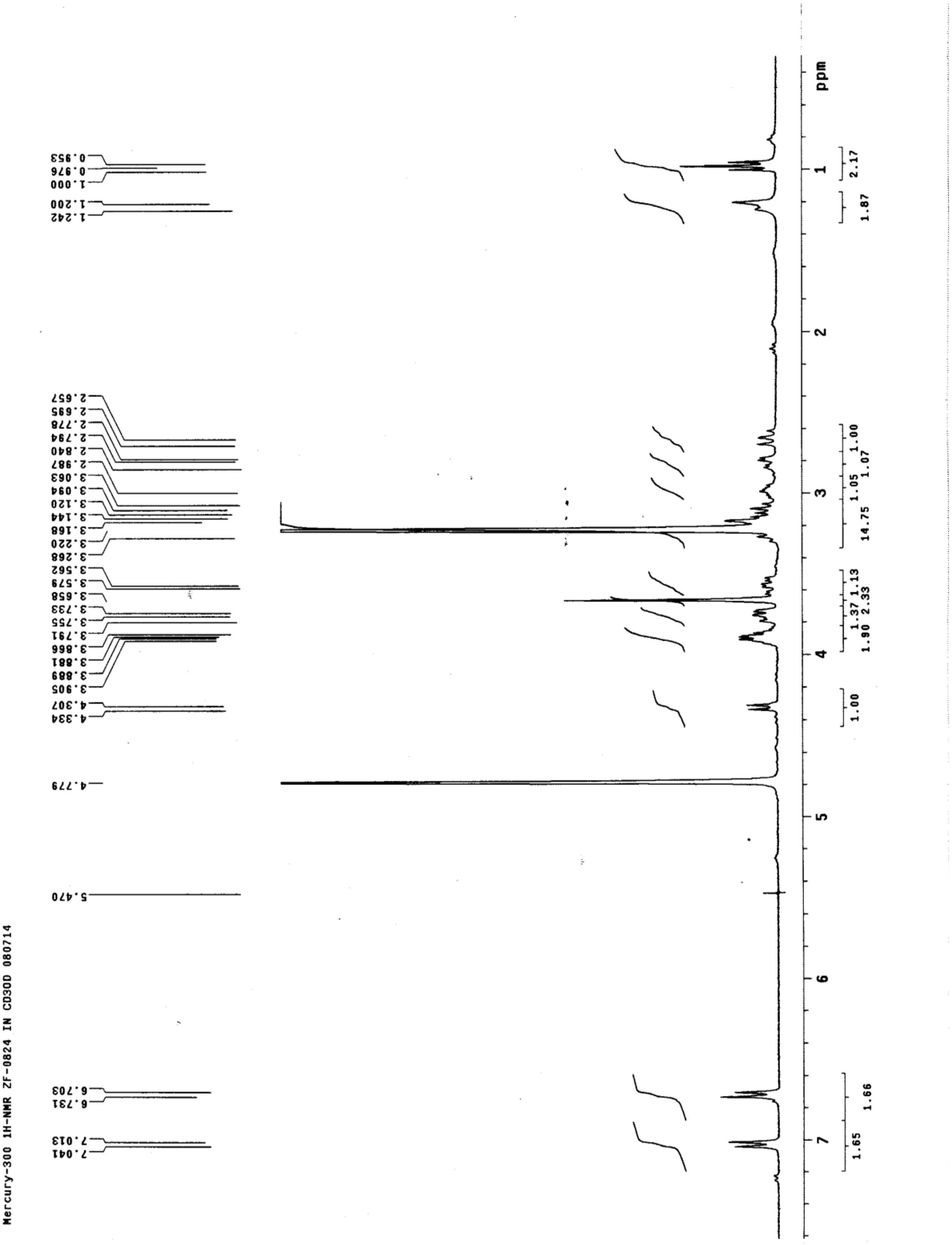

Figure 53. HRMS of compound **6e**.

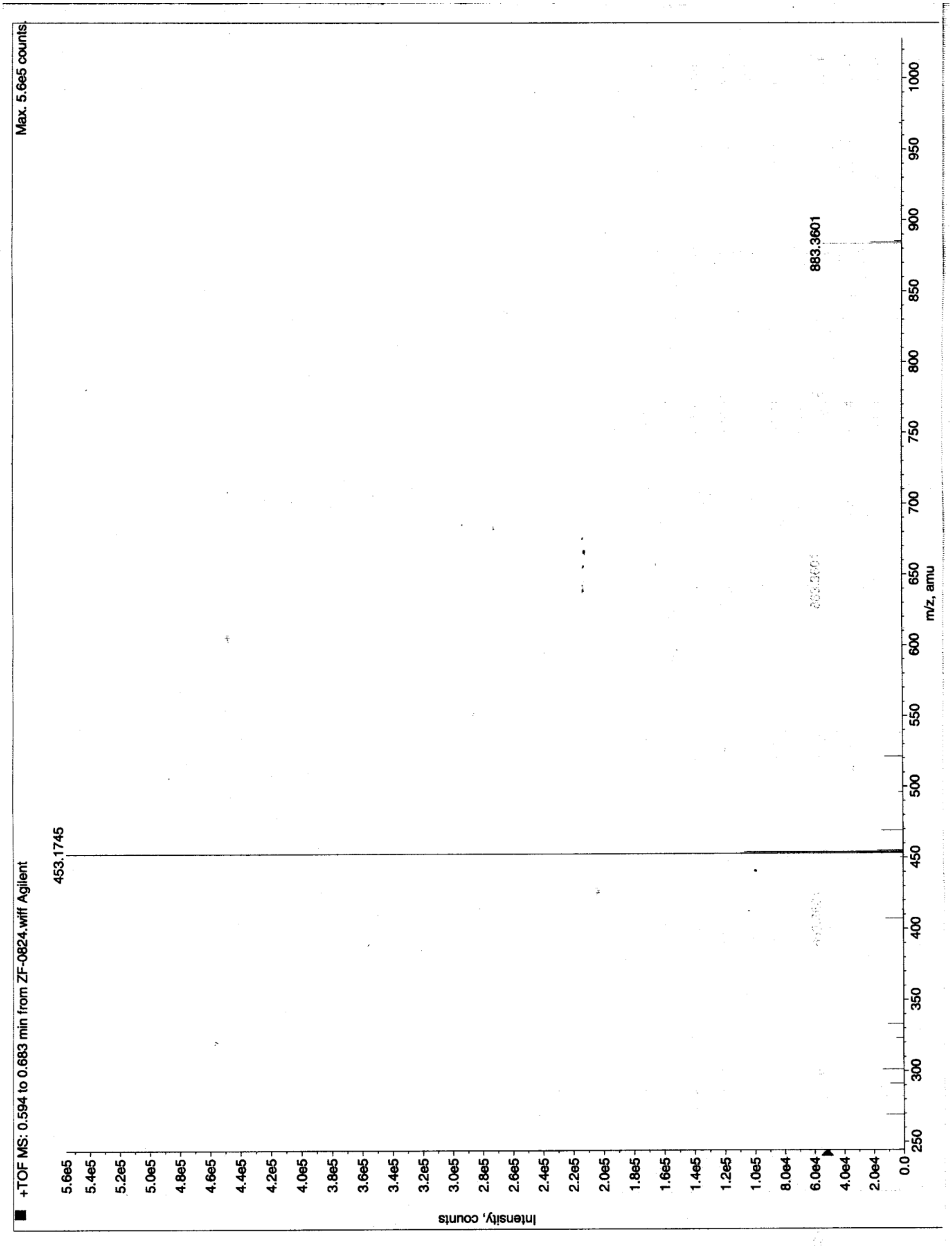

Figure 54. ^{1}H-NMR of compound **7a**.

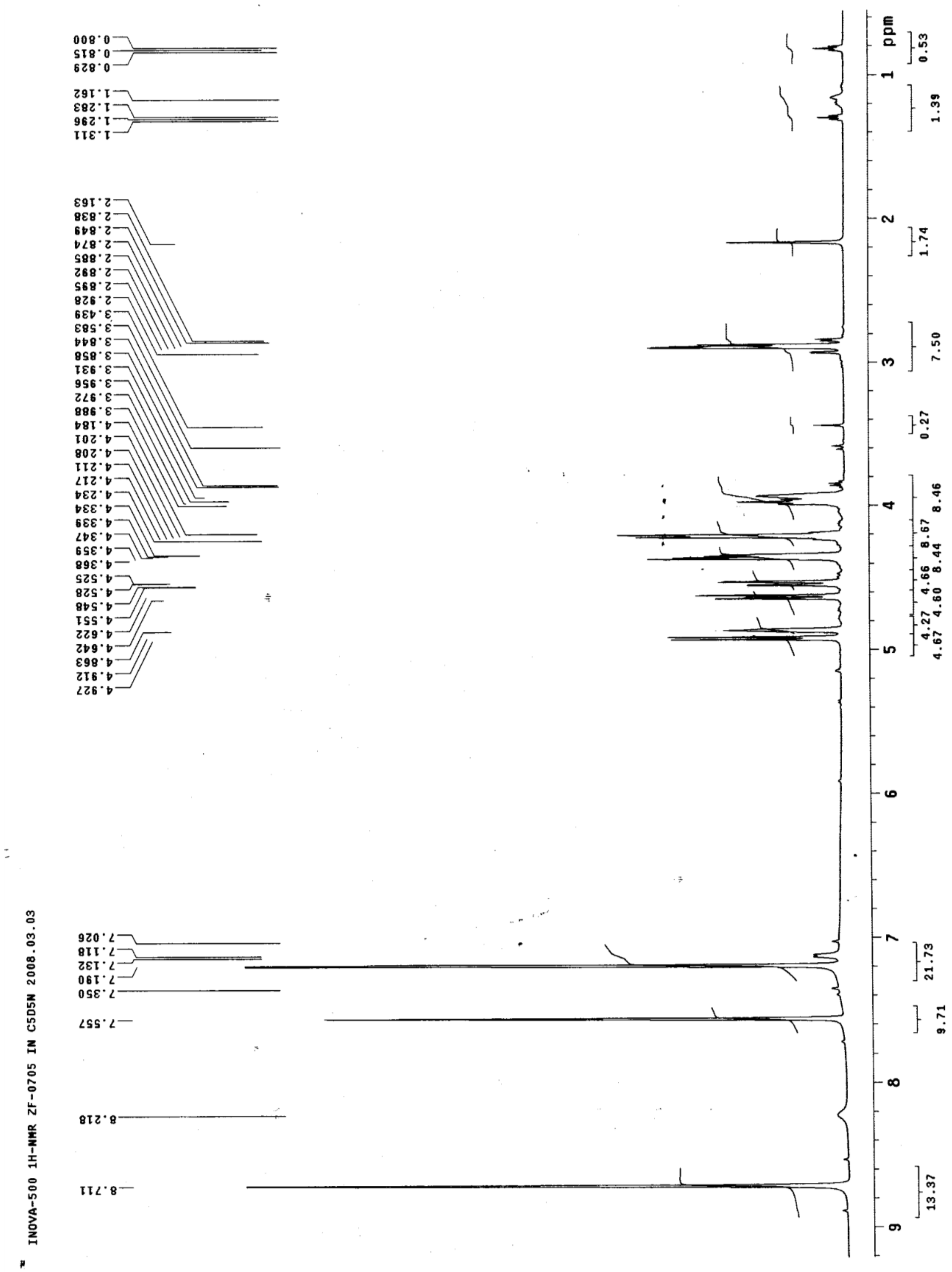

Figure 55. ^{13}C-NMR of compound **7a**.

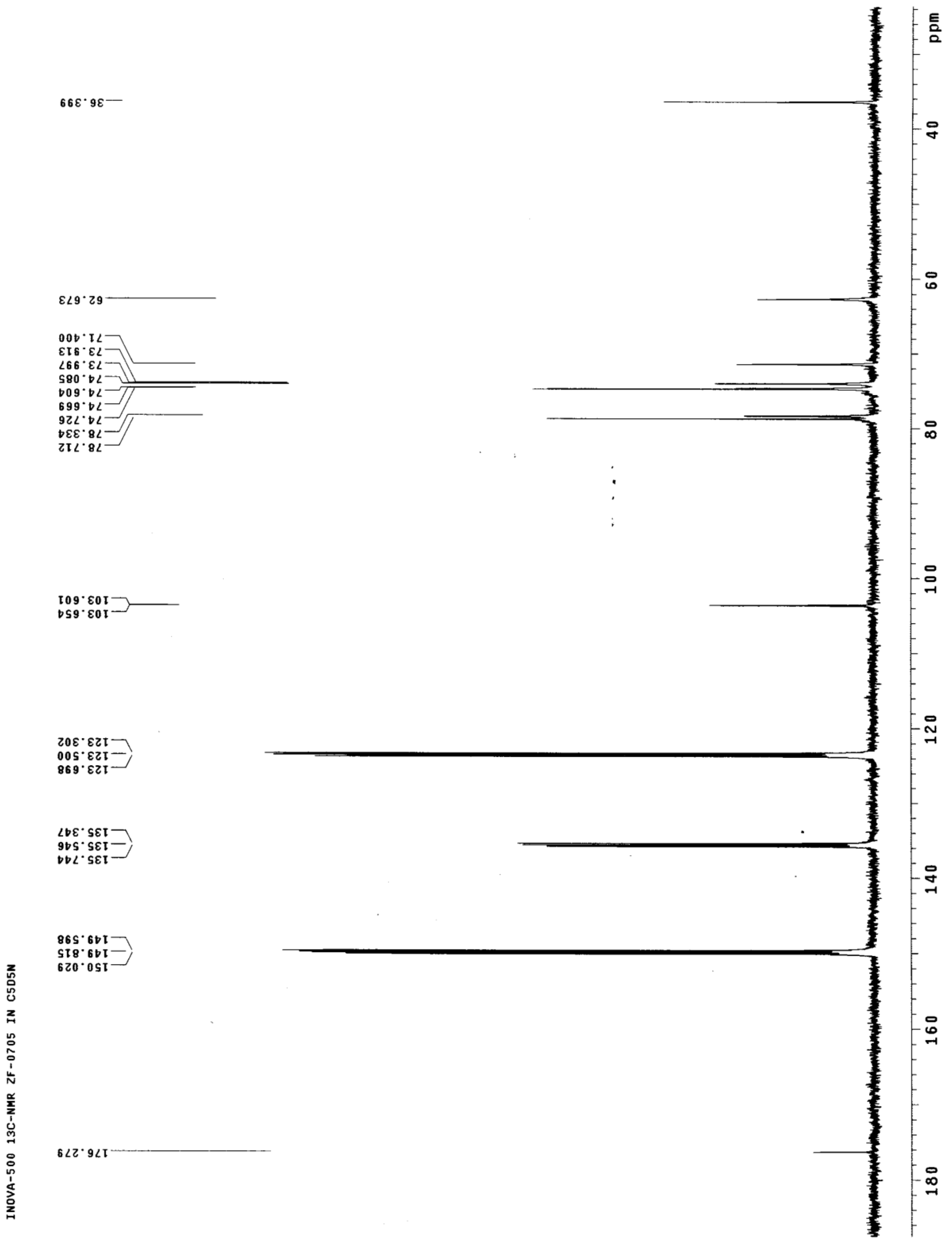

Figure 56. HRMS of compound **7a**.

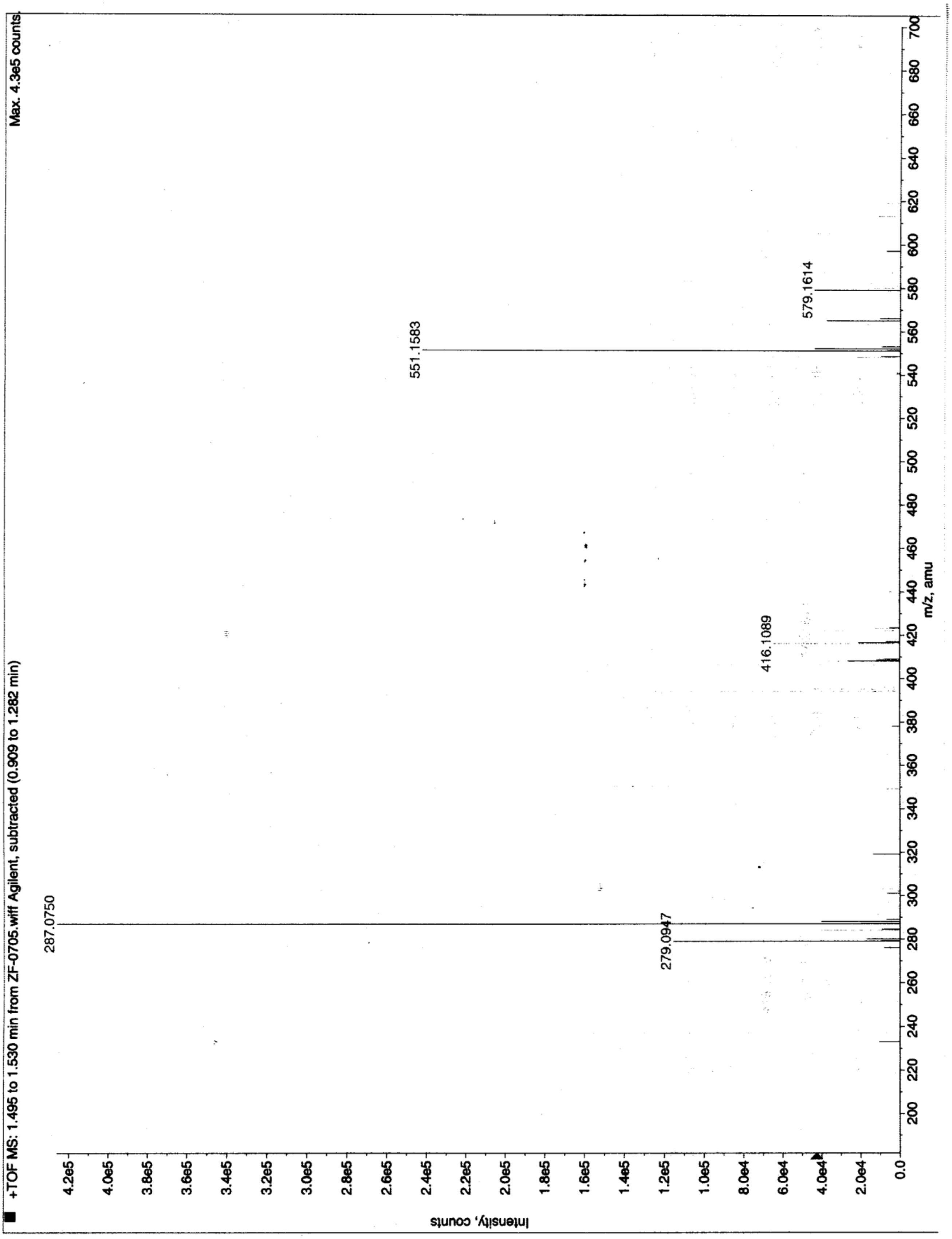

Figure 57. ^{1}H-NMR of compound **7b**.

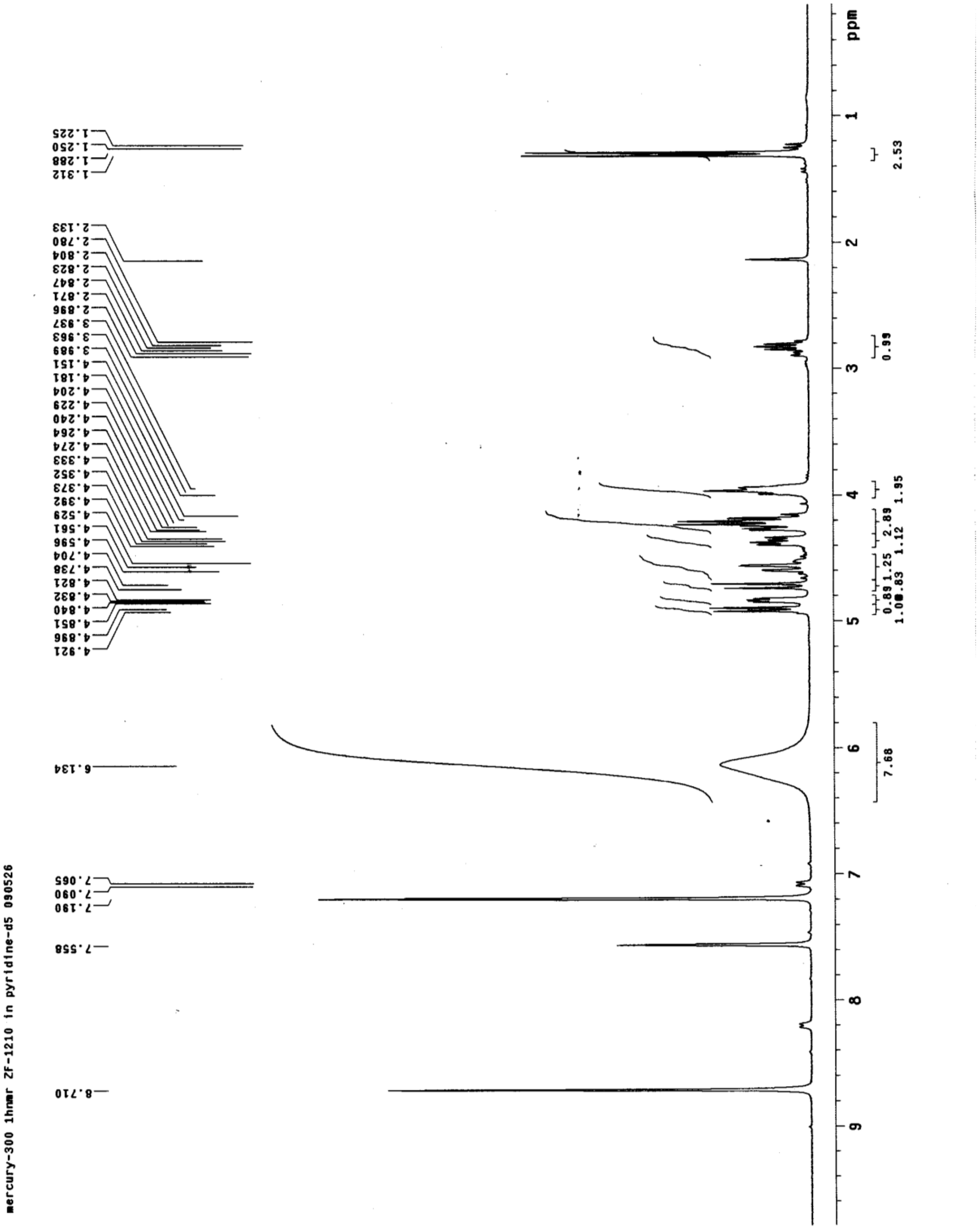

Figure 58. ^{13}C-NMR of compound **7b**.

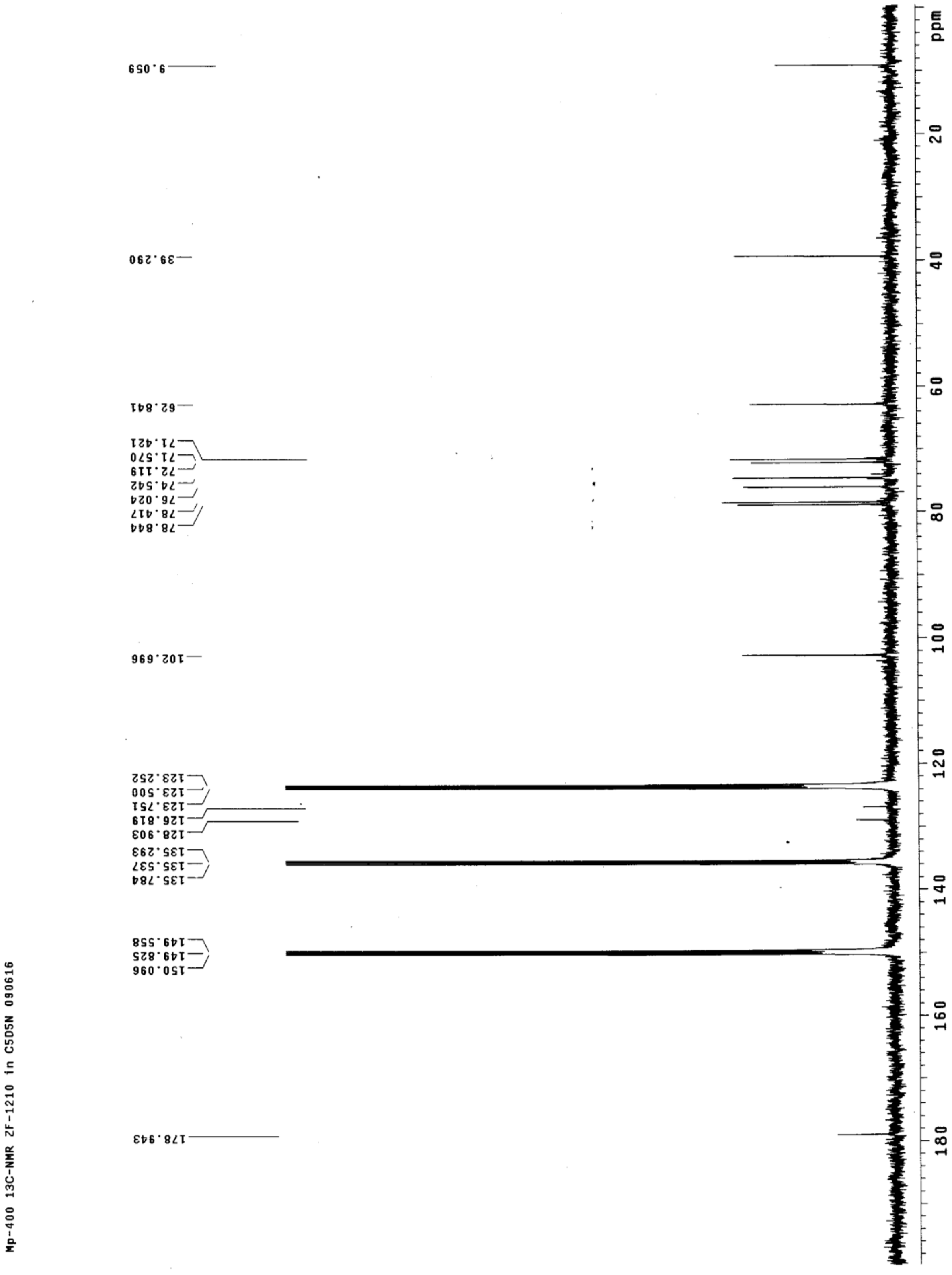

Figure 59. HRMS of compound **7b**.

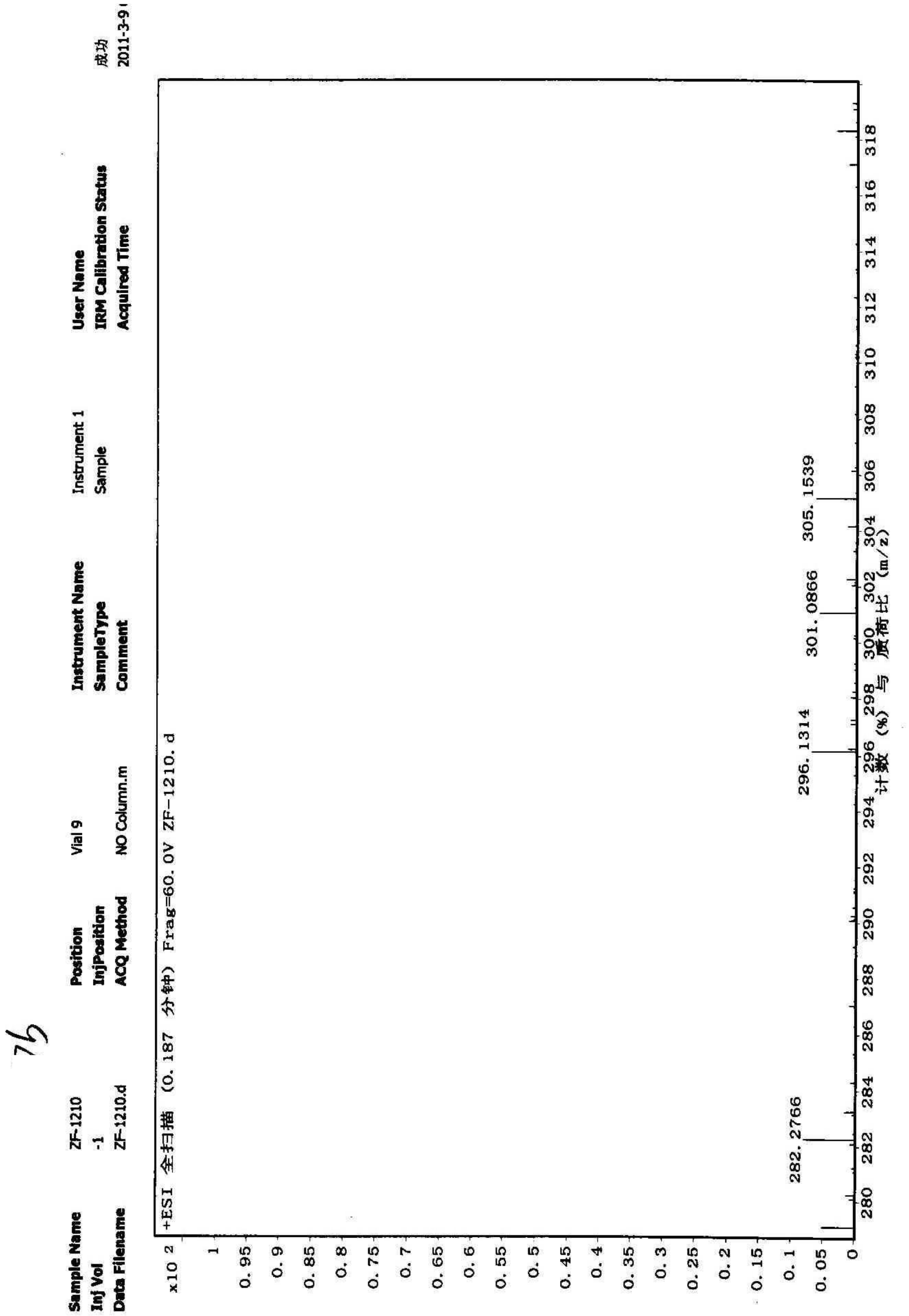

Figure 60. ^{1}H-NMR of compound **7c**.

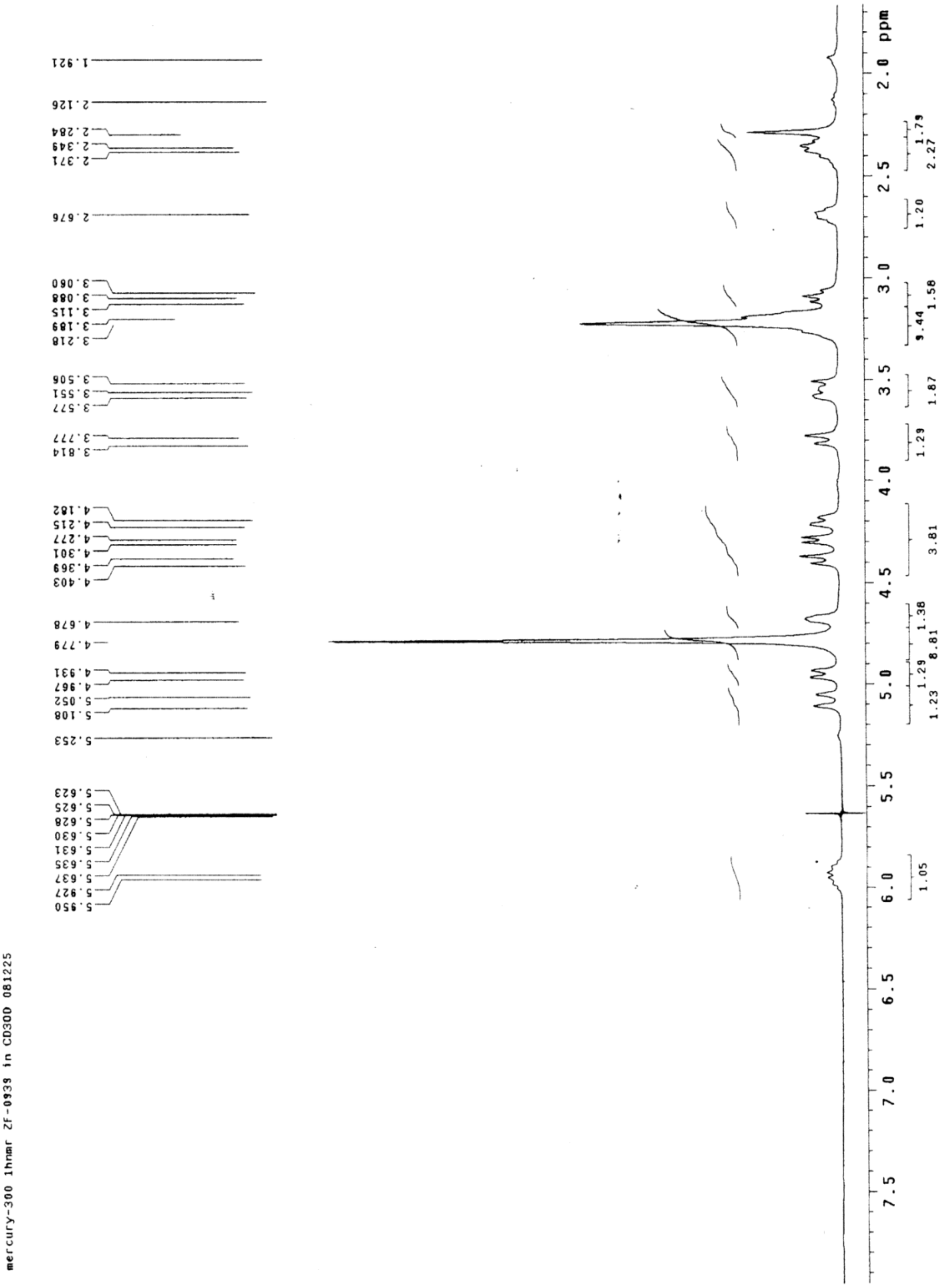

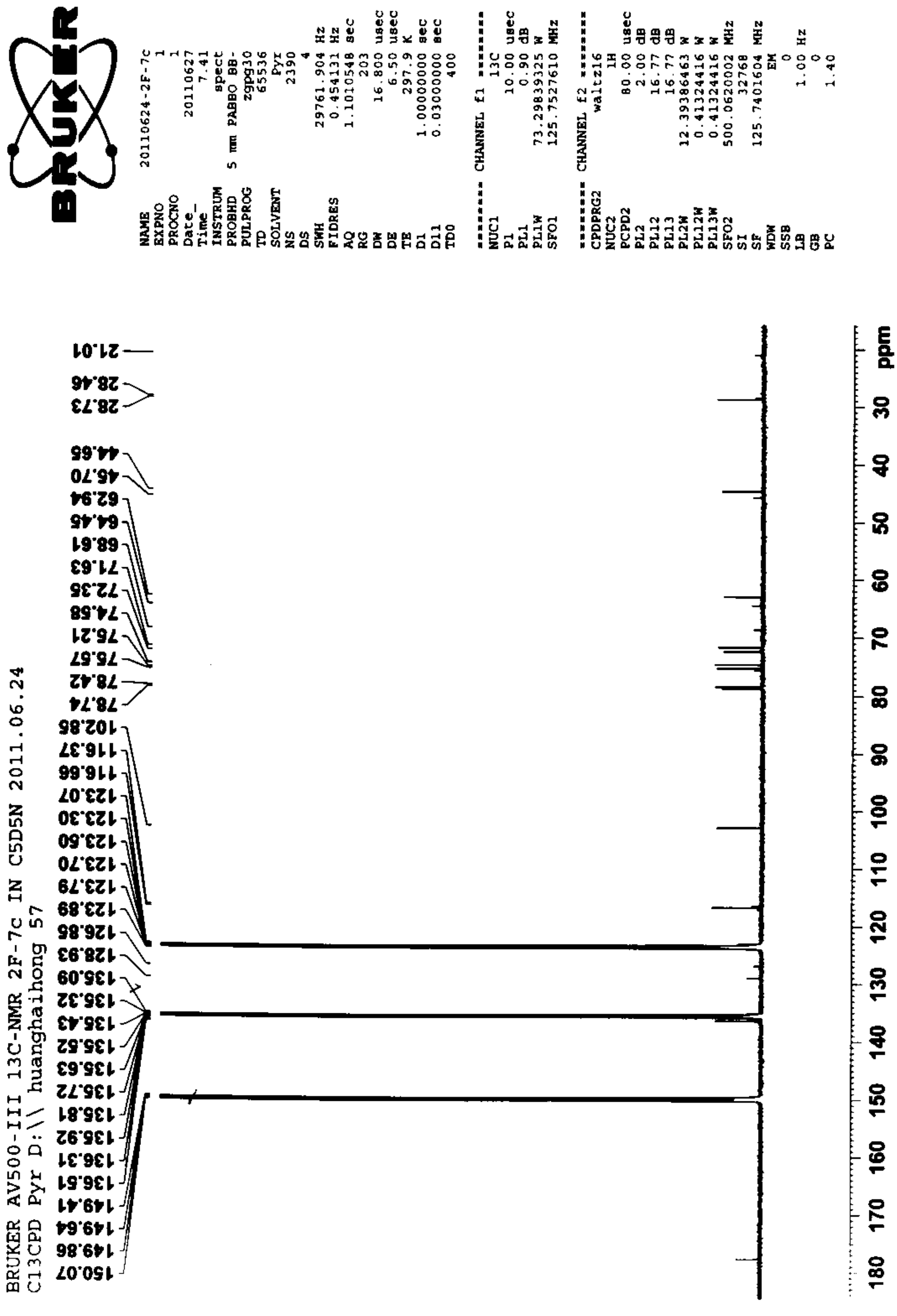

Figure 61. ^{13}C-NMR of compound **7c**.

Figure 62. HRMS of compound **7c**.

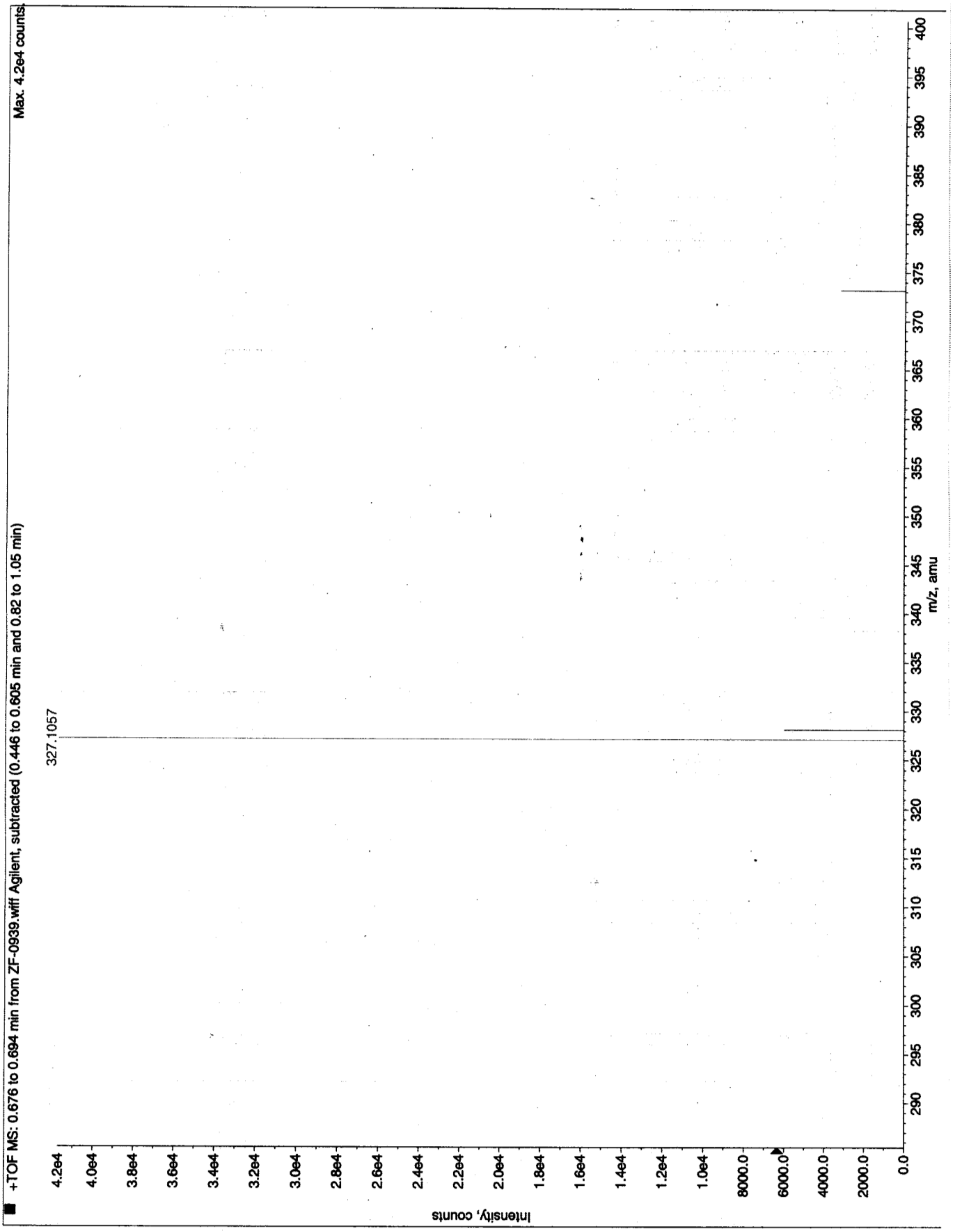

Figure 63. ^{1}H-NMR of compound **7d**.

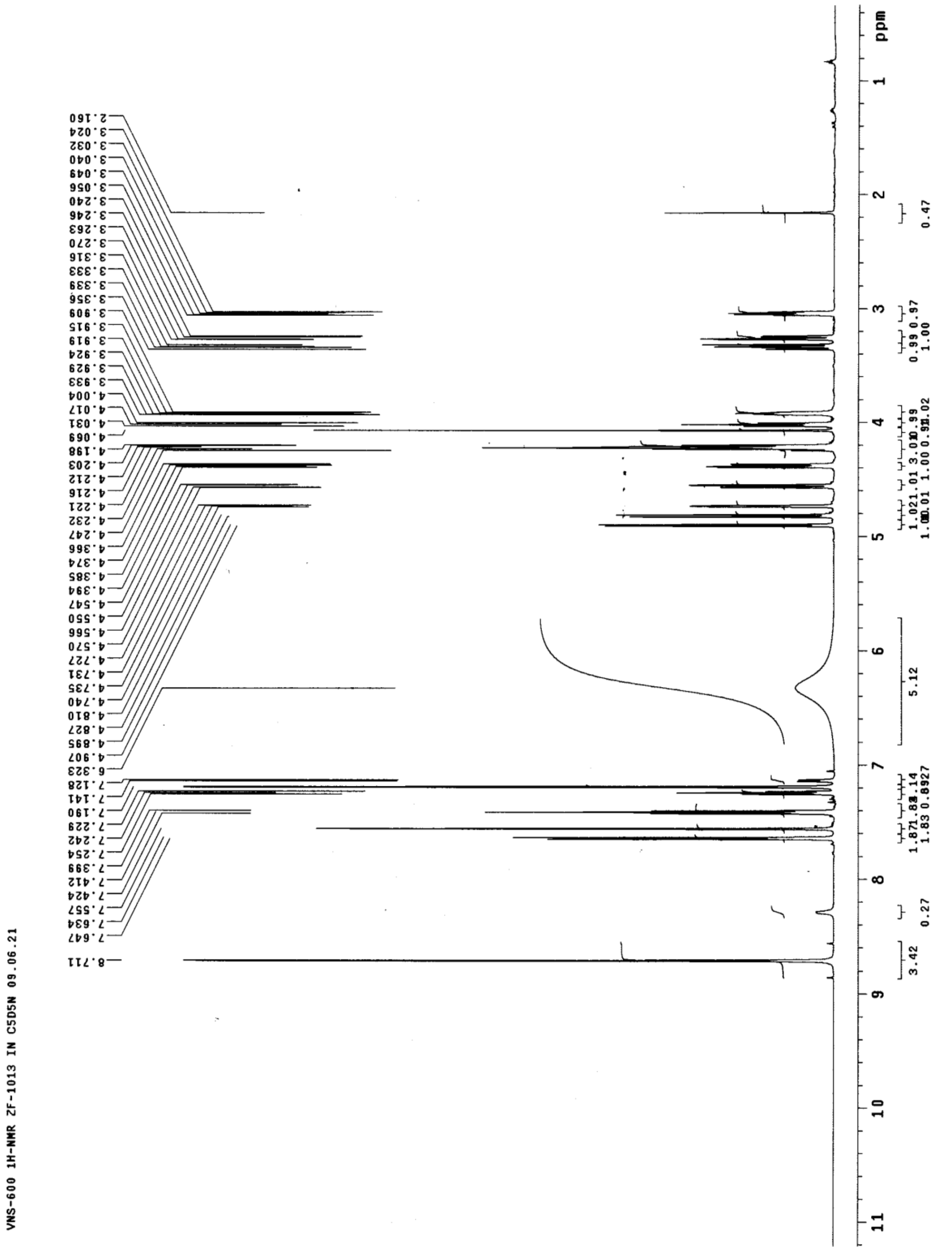

Figure 64. NOE of compound **7d**.

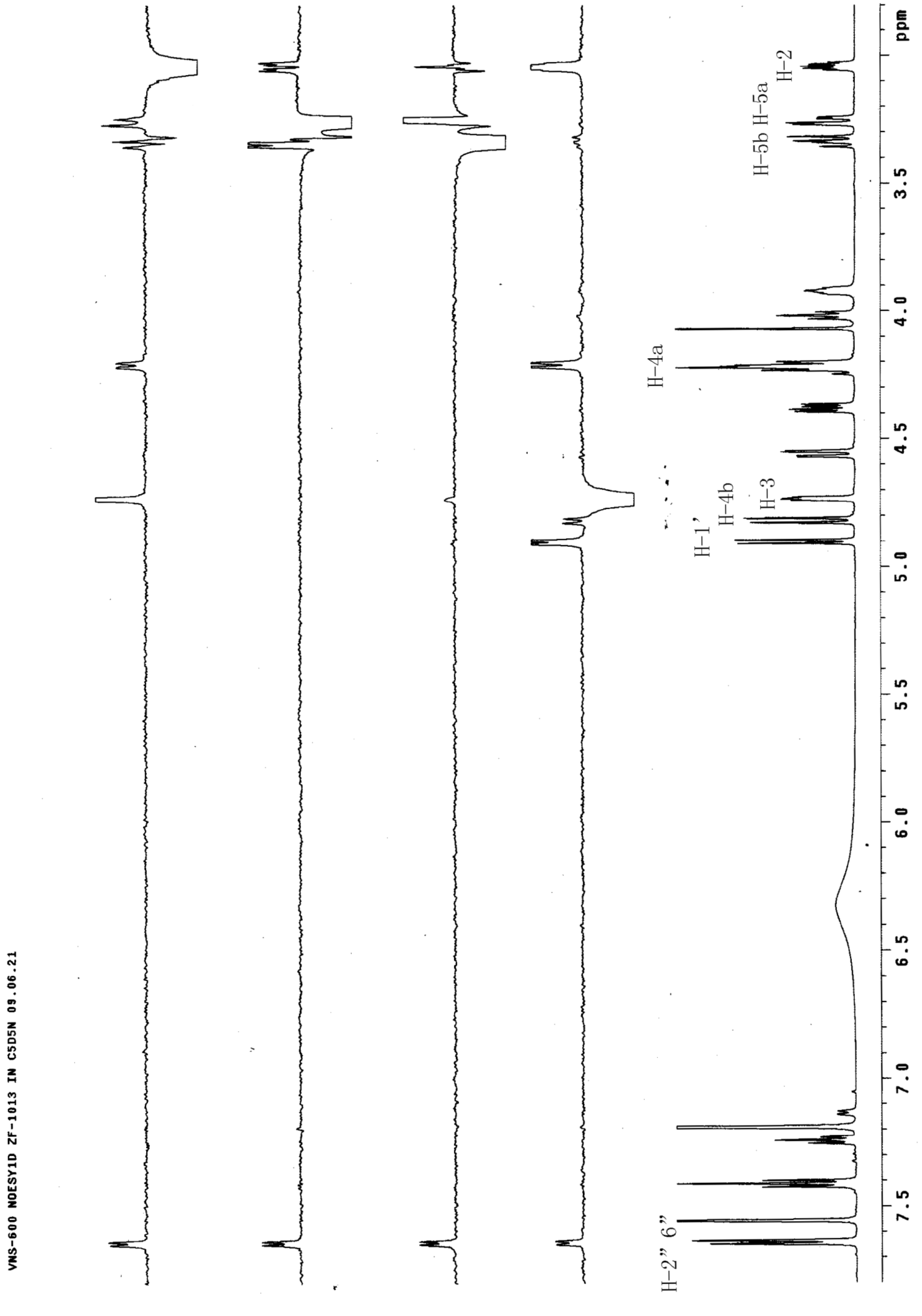

Figure 65. ^{13}C-NMR of compound **7d**.

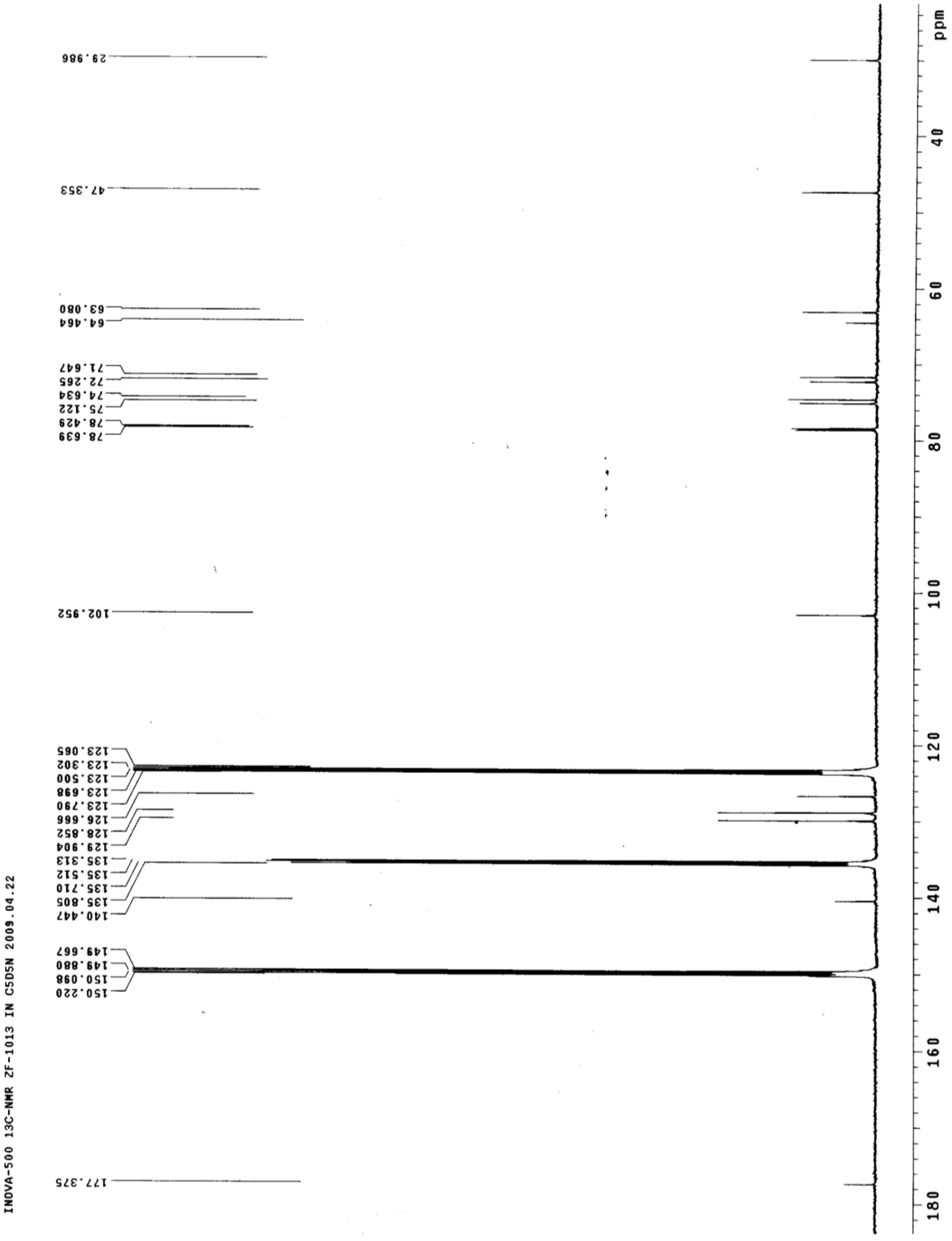

Figure 66. HRMS of compound **7d**.

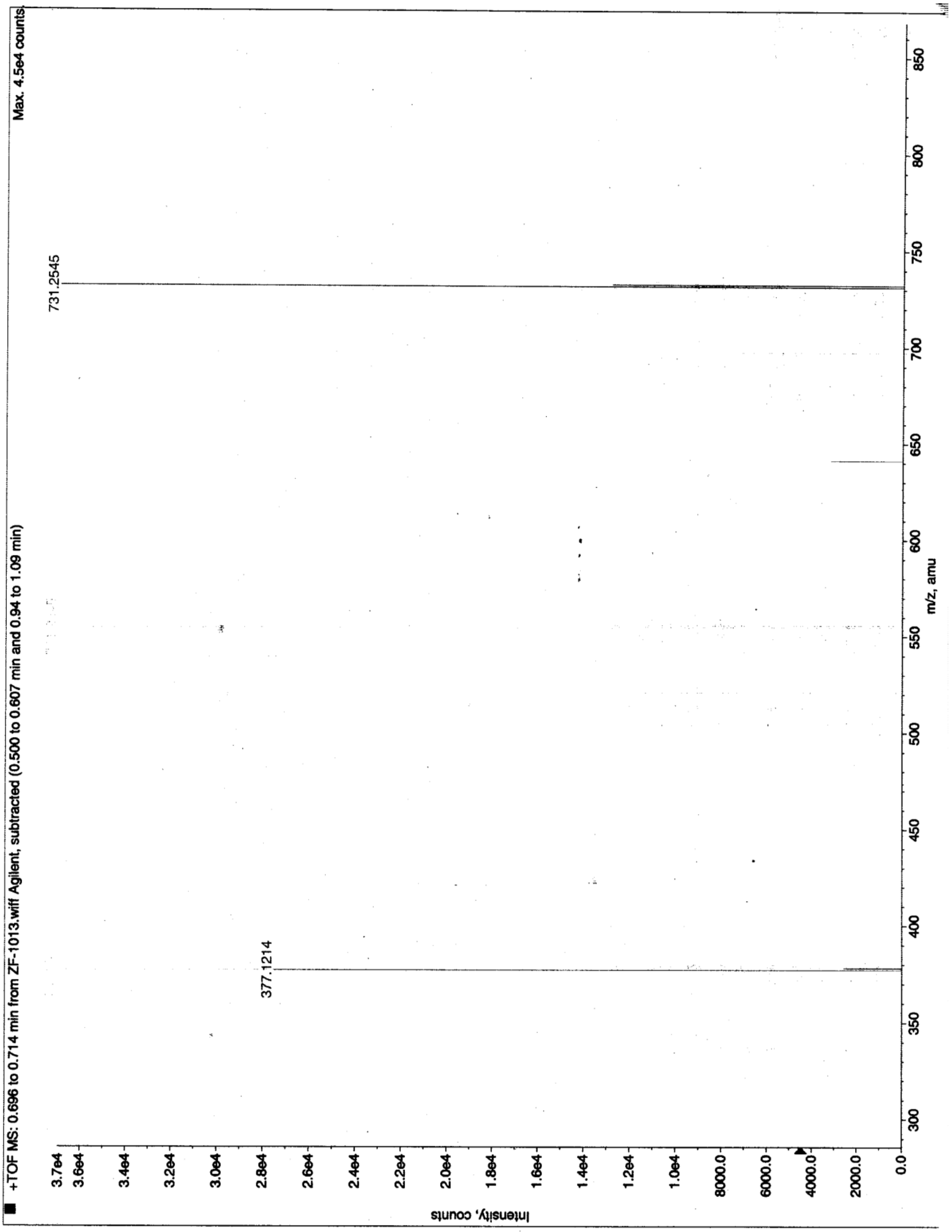

Figure 67. ^{1}H-NMR of compound **7e**.

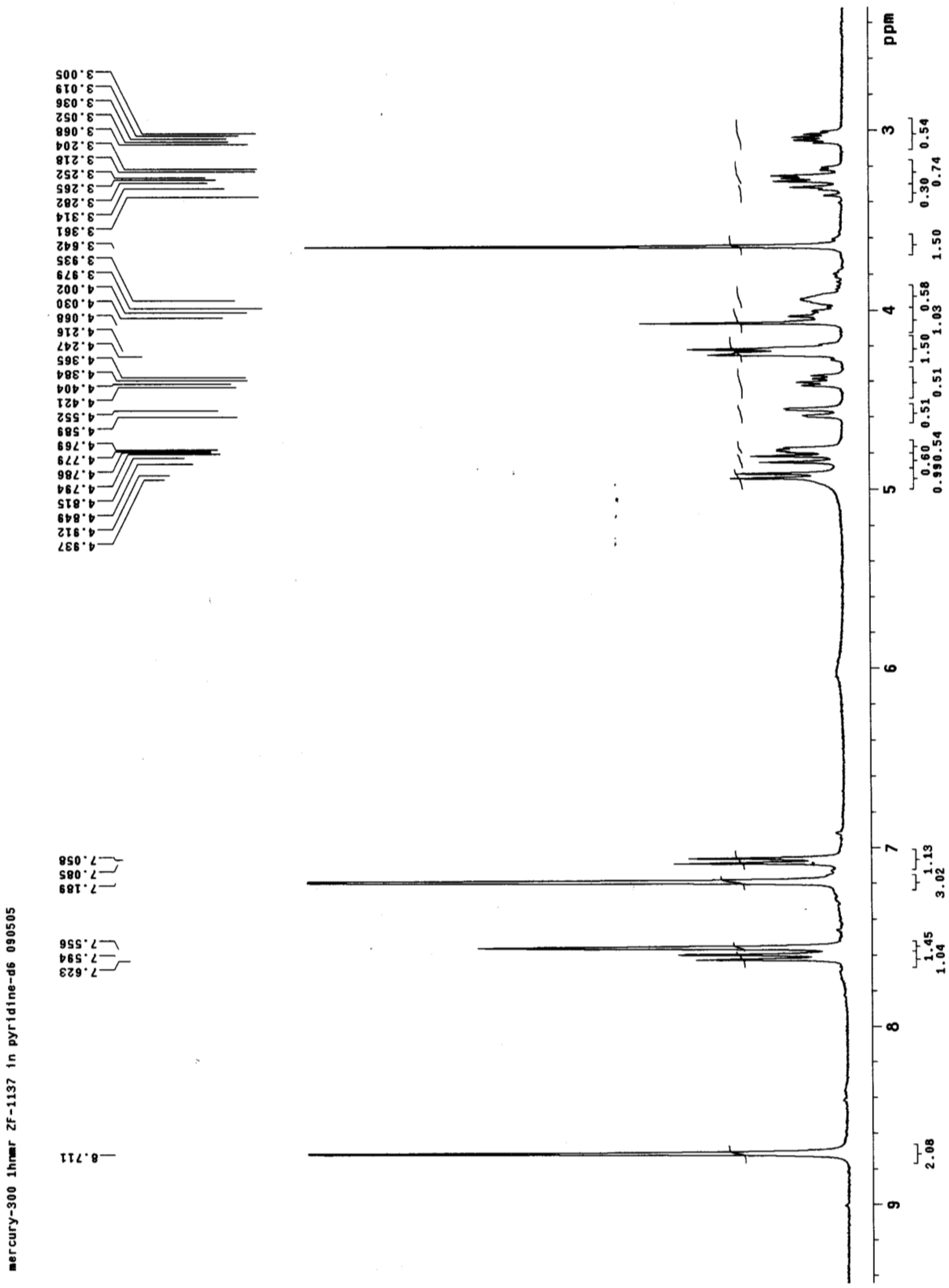

Figure 68. ^{13}C-NMR of compound **7e**.

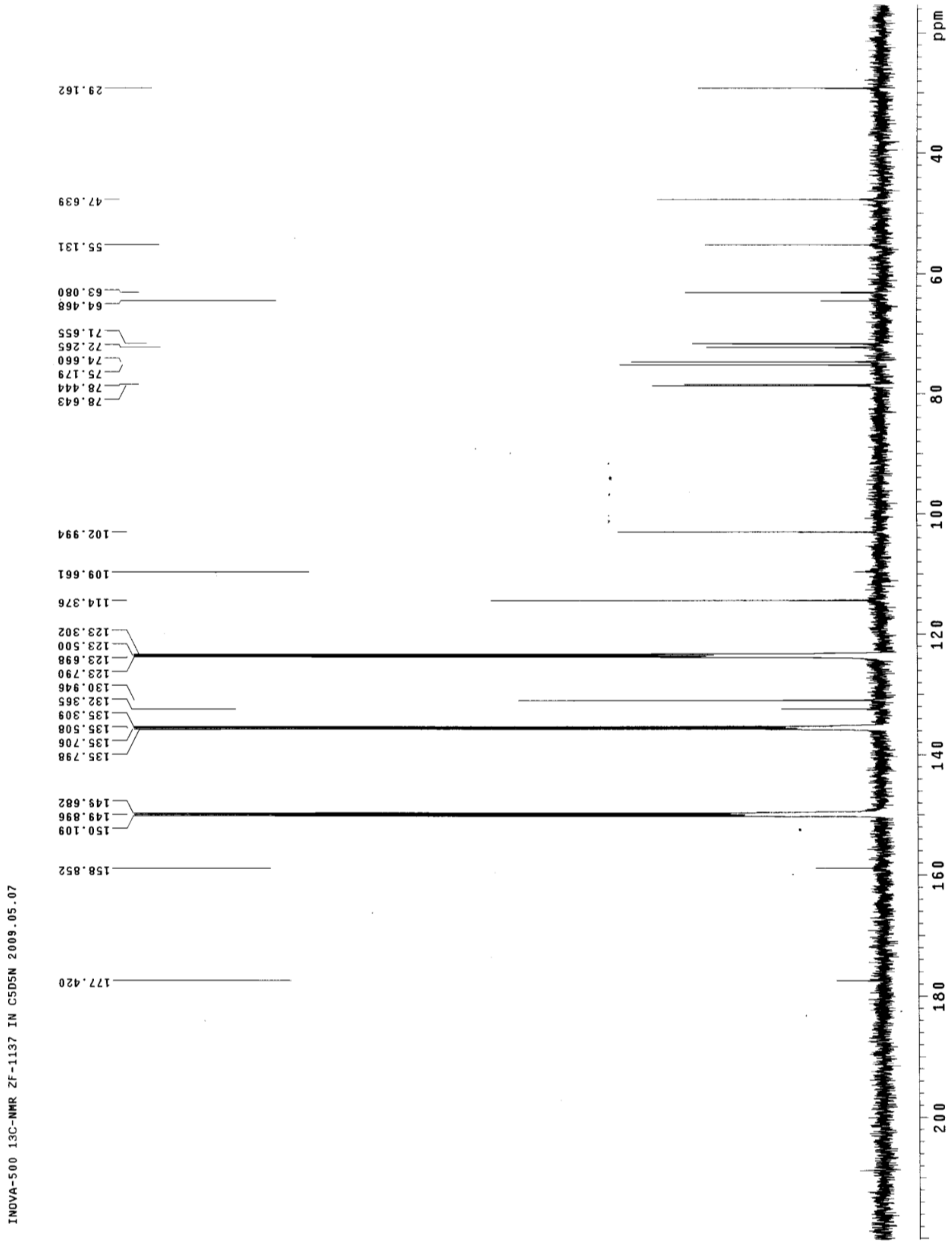

Figure 69. HRMS of compound **7e**.

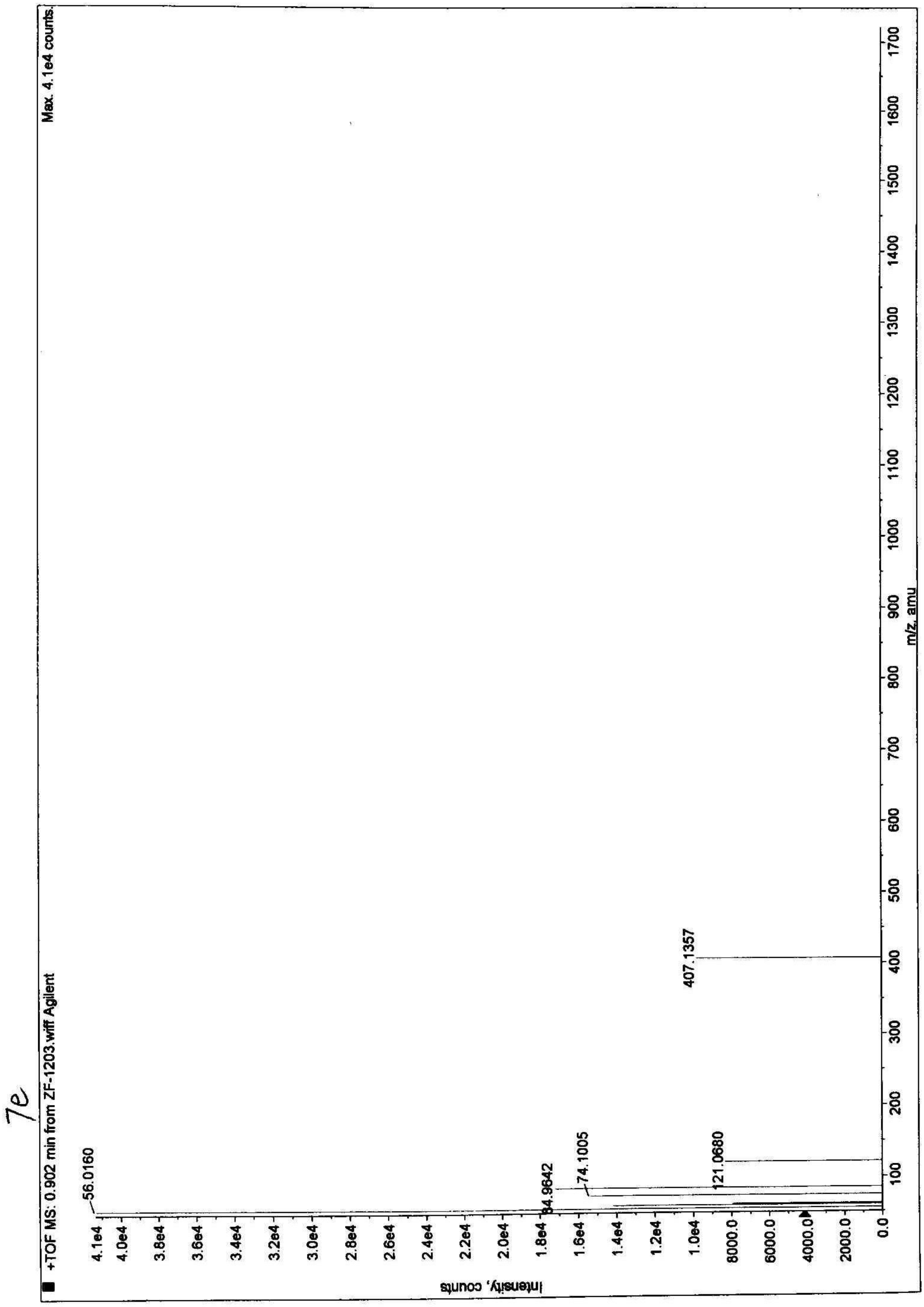

Supplement: Supplementary file 1 [file molecules-18-01933-s001.pdf]
